# On Prototropy and Bond Length Alternation in Neutral and Ionized Pyrimidine Bases and Their Model Azines in Vacuo

**DOI:** 10.3390/molecules28217282

**Published:** 2023-10-26

**Authors:** Ewa Daniela Raczyńska

**Affiliations:** Department of Chemistry, Warsaw University of Life Sciences (SGGW), ul. Nowoursynowska 159c, 02-776 Warszawa, Poland; ewa_raczynska@sggw.edu.pl; Tel.: +48-22-59-37623

**Keywords:** tautomeric aromatic azines, pyrimidine nucleic acid bases, complete tautomeric mixtures, internal effects, tautomeric preferences, bond length alternation, consequences of positive and negative ionization, quantum-chemical data in vacuo

## Abstract

In this review, the complete tautomeric equilibria are derived for disubstituted pyrimidine nucleic acid bases starting from phenol, aniline, and their model compounds—monosubstituted aromatic azines. The differences in tautomeric preferences for isolated (gaseous) neutral pyrimidine bases and their model compounds are discussed in light of different functional groups, their positions within the six-membered ring, electronic effects, and intramolecular interactions. For the discussion of tautomeric preferences and for the analysis of internal effects, recent quantum-chemical results are taken into account and compared to some experimental ones. For each possible tautomer-rotamer of the title compounds, the bond length alternation, measured by means of the harmonic oscillator model of electron delocalization (HOMED) index, is examined. Significant HOMED similarities exist for mono- and disubstituted derivatives. The lack of parallelism between the geometric (HOMED) and energetic (Δ*G*) parameters for all possible isomers clearly shows that aromaticity is not the main factor that dictates tautomeric preferences for pyrimidine bases, particularly for uracil and thymine. The effects of one-electron loss (positive ionization) and one-electron gain (negative ionization) on prototropy and bond length alternation are also reviewed for pyrimidine bases and their models.

## 1. Introduction

Many organic π-electron heterosystems, including natural products, display a particular case of the constitutional isomerism of functional groups called prototropy. This structural phenomenon has been clearly explained, more than eighty years ago, by Pauling [1], who not only showed the fundamental relation between prototropy and resonance, but also indicated the important difference between tautomeric and resonance structures. According to his explanation, prototropic conversions are reversible processes that can run intra- or intermolecularly. During tautomerization, labile proton(s) can move between two or more conjugated functional groups together with the delocalization of π-electrons, leading to the mixture of two or more constitutional isomers, called tautomers. 

Prototropic tautomers always differ by the positions of labile proton(s) and π-electrons [1,2]. The number of possible tautomeric forms is an internal property of the tautomeric molecule. It is a consequence of the number of labile protons and the number of conjugated tautomeric sites. Although the most favored tautomer is very often selected to determine the name and formula of the tautomeric compound, we cannot identify it only with one Lewis structure. Each tautomeric derivative can be described by means of two (or more) structures (tautomers) being in equilibrium, whereas electron delocalization in each tautomer can be described by the corresponding resonance hybrid. For a single tautomer, the number of possible resonance structures results from the position of labile protons and double bonds. A different situation takes place for the relative stabilities of individual tautomers. They strongly depend on various internal and external factors that affect tautomeric preferences. Among the internal factors, the polarity, resonance stability (aromaticity), acidity–basicity of conjugated tautomeric sites, stability of functional groups, and substituents effects, as well as intramolecular interactions, play a particular role. For the external factors, usually, the solvent, pH, excess electron(s), other molecules, ions, radicals, oxidizing or reducing agents, ultraviolet (UV), and γ- and X-ray are considered. 

The Pauling explanation of the prototropy phenomenon [1] has been employed in the IUPAC definition of prototropic tautomerism (IUPAC—International Union on Pure and Applied Chemistry) [3]. Only proton-transfers accompanied by the migration of double bonds refer to prototropic conversions in the tautomeric molecule. In other words, prototropic rearrangements always run in relation with electron delocalization [1,2,3]. The labile proton(s) can move from proton-donor site(s) to proton-acceptor site(s) separated by different conjugated spacers according to 1,3-, 1,5-, 1,7-, 1,9-proton shift, etc. Other intramolecular transfer(s) of H^+^ or H^●^ leading to a separation of positive and negative charges or to a separation of paired electrons cannot be considered as prototropy, and, consequently, zwitterions or polyvalent radicals formed in these processes cannot be classified as prototropic tautomers. 

Prototropic conversions in aromatic heterocompounds, including nucleic acid bases, have been reviewed by Katritzky (died in 2014) and his co-workers in the 1960–2010 period (see, for example, refs. [4,5]). They compiled experimental and computational results mainly for favored tautomers (percentage contents > 1%), and considered most minor (<1%) and all rare tautomers (<0.01%) as negligible in tautomeric mixtures. This kind of treatment of tautomeric systems has led to some discrepancies in the literature, particularly for ionized, protonated, and deprotonated forms, for which prototropy has been usually neglected. Experimental and/or theoretical investigations have been carried out for tautomers that are favored in neutral isomeric mixtures. In the case of pyrimidine nucleic acid bases, the canonical forms or their major tautomers (two or three structures) have been the most frequently considered. These kinds of investigations for the selected isomers are usually partial. Only in the last twenty years have the complete tautomeric equilibria been taken into account for nucleic acid bases, and stabilities of all possible tautomers–rotamers in their various oxidation states (neutral, oxidized, and reduced) analyzed.

In this review, we concentrate our attention mainly on computational results published for the complete tautomeric mixtures of pyrimidine nucleic acid bases and their model compounds. We also discuss some recent experimental results for the favored isomers. We start our review by the principles of tautomeric conversions occurring in nucleic acid bases. We recall the simplest aliphatic and aromatic derivatives possessing the same tautomeric functions as the title compounds. We briefly summarize the application of quantum-chemical methods to structural investigations of nucleic acid bases in the gas phase that models a non-polar environment. Next, we consider all possible isomeric phenomena such as prototropy and, also, the conformational and configurational isomerism of exo groups to write the complete isomeric mixtures for pyrimidine bases and their model compounds. To shed some light on the differences in the isomeric preferences for pyrimidine bases, we pay particular attention to the internal effects possible in vacuo. Taking into account the definition of prototropy, according to which proton-transfer is always related with π-electron delocalization, the bond length alternation could be quantitatively examined for isomeric structures optimized at the same level of theory for both mono-substituted aromatic derivatives and more complex disubstituted pyrimidine bases. In this way, the most important linear trends for geometric and energetic parameters could be selected. Finally, we discuss the consequences of one-electron loss (positive ionization) and one-electron gain (negative ionization) on tautomeric conversions and electron delocalization in the title derivatives.

## 2. Principles of Prototropic Equilibria

Four types of prototropic conversions {keto-enol, imine-enamine, imine-amine (amidine), and/or amide-iminol} can be distinguished for pyrimidine bases, uracil (**U**), thymine (**T**), cytosine(**C**), isocytosine {**iC**—structural part of guanine (**G**)}, and 4-aminopyrimidine {**4APM**—structural part of adenine (**A**)}, as well as for bicyclic purine bases, **G** and **A**, and for their metabolites such as hypoxanthine (**HX**), xanthine (**X**), and uric acid (**UA**). These equilibria are summarized in Table 1 for selected tautomeric moieties. For all of them, the labile proton can move between the conjugated sites according to the analogous scheme of reversible inter- or intramolecular rearrangement accompanied by the migration of the corresponding π-electrons [1,2,3].

Keto-enol tautomerism occurs in derivatives containing the C=O group and at least one H atom at the neighboring ^α^C-sp^3^ or other C-sp^3^ atom separated from C=O by a conjugated spacer {e.g., –(CH=CH)_i_–, *i* = 1, 2, 3, etc.}. In prototropic conversion, H is transferred as a proton from the conjugated C-sp^3^ to O-carbonyl, and vice versa, leading to two tautomeric forms, called keto and enol tautomers being in equilibrium [1,2,3,6]. For the majority of the neutral aliphatic carbonyl compounds, the labile proton prefers C-sp^3^, while the labile π-electrons favor O-carbonyl [7]. This means that the keto isomer predominates in carbonyl compounds more frequently than the enol form. The enol tautomer requires an extra stabilization, e.g., intramolecular H-bond formation and electron conjugation that occur in the enol isomers of β-ketoaldehydes, β-diketones, β-ketoacids, β-ketoesters, and β-ketoamides. The extra intramolecular interactions decrease considerably the enol-form energy in comparison to the keto one that the enol tautomer is favored in the gas phase and non-polar environments [6,8]. Intermolecular interactions with polar solvents (e.g., water) destruct intramolecular H-bonding and diminish the enol-isomer amount in favor of the keto one [9]. 

Analogous isomeric phenomena, such as imine-enamine, imine-amine (amidine), and amide-iminol conversions, occur for other neutral simple tautomeric systems containing heteroatom(s) in the conjugated tautomeric parts [2]. The labile proton can move from one to the other conjugated site, i.e., from C to N, from N to N, or from N to O, respectively, and vice versa. In parallel to proton-transfer, the migration of π-electrons takes place. Compounds containing one tautomeric part without any conjugated spacer are called triad conjugated systems. They display particular amphoteric properties. The sp^2^ atom (with labile π-electrons) is a protonation site (base center) and the sp^3^ atom (with labile proton) is a deprotonation site (acid center). However, there is a principal difference between tautomeric systems and classic amphoteric compounds such as amino acids. Intra- or intermolecular proton-transfer (PT) in amino acid leads to the zwitterionic form, whereas that in a tautomeric system leads to the other isomer (tautomer) without the positive- and negative-charge separation. In the case of simple aliphatic tautomeric triad systems, the more electronegative atom (less basic) prefers the labile π-electrons and forms the double bond with the neighboring atom [2,7,10,11]. The other conjugated atom (more basic) favors the labile proton. Generally, the acid–base properties of the conjugated sites dictate the tautomeric preferences [2,4,5].

In the case of heterocompounds possessing one labile proton and three, four, five, or more conjugated sites, tautomeric conversions become more complex. The labile proton can be transferred according to a 1,3-, 1,5-, 1,7-, 1,9-, or 1,*n*-proton shift, leading to the tautomeric mixture consisting of more than two tautomers [2]. The same is true for tautomeric compounds containing more than one labile proton and more than one pair of conjugated sites. The number of possible tautomers, exactly defined by the number of labile protons and by the number of conjugated sites, is also larger than two. Nevertheless, the principles of proton-transfer and π-electron delocalization are always analogous to those for the triad heterosystems [1,2,3]. Prototropic preferences depend on the acid–base properties of the conjugated sites. Generally, the least basic (least acidic) tautomer predominates in the tautomeric mixture [2,4]. Some exceptions can be found for prototropic derivatives displaying intramolecular interactions between functional tautomeric groups or intermolecular interactions of these groups with other molecules, ions, or radicals [2,4,6,9]. For these kinds of systems, the general rule on tautomeric preferences for neutral isolated compounds can change, because the acid–base properties of tautomeric sites can be different for neutral, ionic, radical, and associated species. 

**Figure 1:**
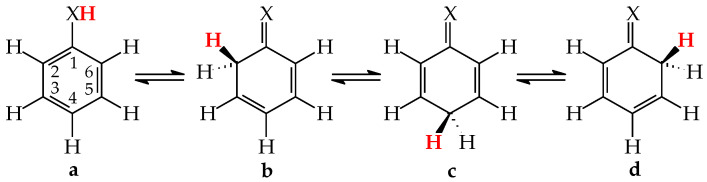
Prototropic equilibria in phenol (X = O) and aniline (X = NH). The labile proton is indicated in bold red color.

Tautomeric aromatic derivatives, for which aromaticity can play a more important role than the general rule of acid-base properties, are very exciting derivatives [4]. For example, mono-hydroxy arenes exhibit keto-enol tautomerism; however, the enol forms most frequently predominate in the tautomeric mixtures [2,12]. The presence of the endo C-sp^3^ atom in the keto forms destructs electron delocalization in the ring, increases the energy of the keto forms, and reduces their amounts in the isomeric mixtures. In other words, the lower-percentage content of the keto forms is a consequence of the higher stability (aromaticity) of the enol isomer. Unsubstituted phenol (C_6_H_5_OH in Figure 1 for X = O), a parent system of mono-hydroxy arenes, is a classic derivative displaying this trend. 

For phenol, the energy of aromatic stabilization is higher than that of prototropy [13], and, consequently, the aromaticity of the ring is a pivotal factor that dictates the higher amount of the enol isomer (hydroxybenzene, **a**) than the keto ones (cyclohexa-2,4- and -2,5-dienones, **b**–**d**; note that **b** has an identical constitution to **d**). The experimental tautomeric equilibrium constants for enolization in an aqueous solution for cyclohexa-2,4- and -2,5-dienones (generated by flash photolysis) [14] are almost the same as those estimated theoretically in the gas phase by various quantum-chemical methods [14,15,16,17,18]. Owing to the insignificant amounts of the keto isomers (<10^−10^%), cyclohexadienones are usually not considered in the structural and acid-base chemistry of neutral phenol. However, in organic chemistry, the keto forms of phenols are frequently used as intermediate structures to explain the mechanism and product(s) formation of various organic reactions such as oxidative metabolism, electrophilic substitution, ionization processes, and the Kolbe-Schmitt and Reimer-Tiemann reactions [12,17,18,19]. 

More complex keto-enol conversions occur for mono-hydroxy arenes containing two or more condensed rings [16,20]. For example, 9-anthrol is only slightly less stable than its keto isomer, 9-anthrone. In the isomeric mixture, the keto form coexists with the enol tautomer. An extra stability of 9-anthrone originates from an aromatic character of two marginal rings. A slight amount of the keto form (<1%) can also be found in 9-phenanthrol. However, the keto isomers of 1- and 2-naphthols can be neglected in the isomeric mixtures. The same is true for those of 1- and 2-anthrols, as well as of 1-, 2-, 3-, and 4- phenanthrols. Nevertheless, the percentage contents of some of them are considerably higher (>10^−6^%) than those of the keto forms of unsubstituted phenol (<10^−10^%). Mono-hydroxy azulenes (azulenols), constitutional isomers of naphthols, are also interesting arene derivatives. Contrary to 1- and 2-naphthols, at least one keto form containing the labile proton at the C atom of the five-membered ring significantly contributes to the isomeric mixtures of azulenols [21]. For example, the keto and enol isomers of 2- and 5-hydroxyazulenes coexist in almost equal amounts in their gaseous isomeric mixtures. The percentage contents of the keto forms in other azulenols (1-, 4-, and 6-hydroxyazulenes) are not larger than 1%. The high amounts of the keto tautomers are strongly related with the polarity of the azulene system. Other factors, such as the acidity-basicity of endo CH/CH_2_, also seem to play an important role in the tautomeric composition of azulenols. Owing to the azulene-system polarity, larger amounts of the keto-isomers are found in the gas phase (non-polar environment) than in the aqueous solution (polar medium). 

The six-membered aromatic ring is also present in the mono-amino arene—aniline (C_6_H_5_NH_2_). Like C_6_H_5_OH, it contains one labile proton and four conjugated sites (Figure 1 for X = NH). However, amino benzene displays the other type of prototropy (enamine-imine tautomerism) compared to phenol [22]. The labile proton can move according to the 1,3-, 1,5, or 1,7-proton shift from the exo NH_2_ group to the endo C-sp^2^ atom being at the 2-, 4-, or 6-position vis-à-vis NH_2_. Four tautomers are thus possible for aniline (**a**–**d**, where **b** and **d** possess an identical constitution). The aromatic enamino form **a** dictates the tautomeric preference in aniline. The transfer of the labile proton to the ring C-sp^2^ and its transformation into C-sp^3^ strongly changes the delocalization of π-electrons in the imino tautomers **b**–**d**, reduces the stability of the ring, and increases the energies of **b**–**d**, like for the keto forms of phenol. From a physicochemical point of view, the imino tautomers can be neglected in the tautomeric mixture. They are exceptionally rare isomers of aniline (<10^−15^% [22]). However, the imine forms are often used as intermediate structures to understand the mechanism of various chemical reactions and to confirm the product formation [19,22,23,24,25,26]. The resonance energy (aromatic stability > 30 kcal mol^−1^ [27]), being considerably higher than the energy of prototropy in aniline, can only explain the change of isomeric preference when going from simple aliphatic to aromatic enamine-imine tautomeric systems. 

Biomolecules, such as nucleic acid bases, their metabolites, and model compounds, consist of aromatic ring(s) with the exo NH_2_ and/or OH groups and endo N atoms. They exhibit various types of prototropic equilibria (given in Table 1) that originate mainly from the presence of the exo groups, conjugated with the corresponding endo atoms (N and C) [2,4,5]. In the case of pyrimidine bases (**U**, **T**, **C**, and **iC**) containing one six-membered ring with two endo N atoms and two exo groups (NH_2_ and/or OH), two labile protons can move between the conjugated sites according to the analogous scheme of proton-transfers as that given in Figure 1 [28]. For bicyclic purine bases (**A** and **G**) and their metabolites (**HX**, **X**, and **UA**) containing the six-membered pyrimidine fragment structurally fused with the five-membered imidazole ring, the imidazole part contains additional labile proton(s) and additional conjugated sites that can also participate in prototropy [29,30,31,32,33,34]. Consequently, the tautomeric equilibria for purine derivatives are more complex than those for pyrimidine bases. For adenine and its model compounds (imidazole, **4APM**, and purine), the complete prototropic conversions have been already reviewed [35]. 

## 3. Quantum-Chemical Methods Applied to Tautomeric Nucleobases in Vacuo

About seventy years ago, prototropy in nucleic acid bases has been reminded and a hypothesis of rare tautomers formulated as one of the principal reasons for mutations, replication, and degradation processes in nucleic acids [36]. Since that time, the phenomenon of prototropy has been intensively examined and discussed for nucleic acid bases and their model heterocompounds, and some theories on spontaneous point mutations in nucleic acids formulated [37,38,39]. This phenomenon permanently attracts the attention of researchers from the many disciplines of life sciences, chemistry, biochemistry, molecular biology, biotechnology, veterinary, medicine, pharmacology, radiology, etc.

Computational documents on prototropy in heterocompounds have already been reviewed in the 1970s by Katritzky and his co-workers [4]. With the development of quantum-chemical methods and computational techniques, the level of computations for isolated molecules increased from semi-empirical (e.g., MNDO, AM1, and PM3), to high DFT and ab initio levels (e.g., HF, MP*n*, CCSD(T), QCISD(T), and G*n*). In the literature, we can find thousands of theoretical works in this field, reported by chemists from different countries, e.g., articles of Person, Kwiatkowski, Hillier, Fraga, Catalán, de Paz, Adamowicz, Leś, Luque, Orozco, Leszczyński, Katritzky, Fabian, Hobza, Šponer, Tureček, their co-workers, and many others. Most of them have already been reviewed (see in refs. [2,4,5,40,41,42]). Although quantum-chemical computations offer the possibility of investigating all possible tautomers, including, also, those experimentally inaccessible, to our knowledge, the complete tautomeric mixtures for heterocyclic biomolecules have not been investigated in the 1960–2000 period. This is probably because chemists investigated earlier tautomeric compounds by experimental techniques and found the major and some minor tautomers. Only these forms and, additionally, some other minor isomers have been considered in quantum-chemical calculations.

The structural complexity of the tautomeric biomolecule does not mean that all possible isomers cannot be determined. Taking the principles of prototropy into account, the structures of all possible tautomers can be written, and, next, their stability studied by quantum-chemical methods. Fortunately, the intensive development of quantum-chemical methods and computational techniques in the last twenty years considerably reduced the limits of their applications and time of calculations. Detailed investigations on the complete tautomeric mixtures of nucleic acid bases, their model compounds, and metabolites could be carried out [28,29,30,31,32,33,34,35]. An interesting theoretical procedure (TauTGen—tautomer generator program) for searching the favored tautomers (isomers) has been proposed in 2007 by Harańczyk and Gutowski [43]. According to this procedure, we can generate a library of any type of isomeric forms (tautomers–rotamers as well as zwitterions and polyvalent radicals) for tautomeric compounds, and, next, find the low-energy tautomeric structures by means of quantum-chemical calculations. This program can be applied for neutral, as well as for charged, tautomeric species. Presently, quantum-chemical methods can be used not only for structural (isomeric) analyses, but also for investigations of various physicochemical properties and reaction mechanisms, even for more complex molecules than nucleic acid bases (see, for example, refs. [44,45,46,47]). 

A different situation occurs for experimental techniques applied to isomeric systems in the gas phase, solution, and solid state, e.g., ultraviolet (UV), infrared (IR), microwave (MW), nuclear magnetic resonance (NMR), mass spectrometry (MS), ion cyclotron resonance (ICR), photoelectron spectroscopy (PES), X-ray, etc. [2,4,5]. These methods usually have their own limits. We can even find in the literature different numbers of favored tautomers detected for the same tautomeric molecule by different experimental methods. These discrepancies can be explained as follows: Tautomeric conversions are very fast and reversible processes and it is difficult to isolate and to investigate single tautomers by experiments [4,5]. Prototropic equilibria are also very sensitive to experimental conditions. [2,4,5,18,21,22,28,29,30,31,32,33,34,35]. A slight change of environment and even the method of vaporization can affect the composition of tautomeric mixtures and tautomeric preferences. Moreover, isomeric forms can be analyzed experimentally only in the case when they are stable enough during measurements, their amounts are sufficiently high, and their signals are distinguishable from the background. Owing to these limits, mainly major and some minor tautomers can be experimentally detected. Most minor isomers (<0.1%) cannot be identified, and rare isomers (<0.01%) are usually undetectable. In spite of these inconveniences, experiments always play a pivotal role in organic chemistry and biochemistry. However, the applications of quantum-chemical methods come now before those of experimental ones, particularly in structural and proton-transfer chemistry in the gas phase [16,17,18,21,22,27,28,29,30,31,32,33,34,35,43,44,45,46,47].

In 2004, while preparing a review article on tautomeric equilibria and analyzing numerous tautomeric systems [2], we found no literature data for rare tautomers of nucleic acid bases, as well as for their model compounds, i.e., for CH forms analogous to those for phenol and aniline—structures **b**–**d** in Figure 1. For this reason, we decided to consider all possible tautomeric forms (major, minor, and rare isomers) in our computational studies for prototropic heteroaromatic compounds [28,29,30,31,32,33,34,35,48,49]. For our investigations, we chose one level of theory, the application of which could not only provide complete information on all possible isomeric forms and on all possible isomeric rearrangements, but also could give the possibility of deriving some quantitative conclusions on the structural and energetic parameters, as well as on some specific internal effects that dictate the isomeric-stability orders and tautomeric preferences. 

Among various quantum-chemical methods applied in proton-transfer chemistry, we selected the density functional theory (DFT) method [50] with the three-parameter hybrid exchange functional of Becke [51] and the correlation functional of Lee, Yang, and Parr (B3LYP) [52], and larger basis sets with the diffuse and polarization functions [53] {DFT(B3LYP)/6-311+G(d,p)}. The DFT level of theory is sufficient for quantitatively describing the proton-transfer reactions (including prototropy) for heterocompounds [18,21,22,28,29,30,31,32,34,35,48,49]. In particular cases, we also used the Gaussian-*n* (G*n*) theories [54,55,56], which need more computational time than the DFT methods. For the majority of the investigated tautomeric heterocompounds, the G*n* theories lead to results analogous to the selected DFT methods [22,48,49,57]. 

Prototropic equilibria between tautomers can be quantitatively described by thermochemical quantities such as relative energies (Δ*E*), relative enthalpies (Δ*H*), relative Gibbs energies (Δ*G* = Δ*H* − *T*Δ*S*), and/or equilibrium constants {*K* = exp(−Δ*G*/*RT*)}, all calculated at 298.15 K. The mole fractions *x*_i_ of individual isomers can be found on the basis of the estimated relative Gibbs energies (*x*_i_ ≈ [exp(−Δ*G*_i_/*RT*)]/{Σ_1_^n^[exp(−Δ*G*_i_/*RT*)]}) or equilibrium constants {*x*_i_ = *K*_i_/(Σ_1_^n^*K*_i_)}. In the Supplementary Materials, we summarized the Δ*G*s for the neutral, positively, and negatively ionized isomers of phenol and mono-hydroxy azines (Table S1), aniline and mono-amino azines (Table S2), as well as pyrimidine bases (Table S3), all estimated at the same DFT level of theory and taken from refs. [18,22,28,49,57,58,59,60,61,62,63,64,65]. The DFT results for protonated and deprotonated tautomers–rotamers of cytosine and isocytosine have just been reported for the complete tautomeric mixtures [66]. 

## 4. Geometry-Based HOMED Index

Organic derivatives possessing π-electrons are usually more or less delocalized [1,2]. Electron delocalization depends on the number and positions of π-electrons and also on the number and positions of heteroatoms in the molecule. It also depends on the structure of the organic compound, which can be cyclic or acyclic. For tautomeric heterocompounds, π-electrons can be delocalized in individual isomers by a mixture of σ-π hyperconjugation, n-π conjugation, π-π conjugation, and/or aromaticity. For example, full electron delocalization occurs for aromatic isomers (or for their fragments) containing π-electrons in the ring(s), the number of which is in accord with the Hückel rule. Medium electron delocalization can take place for acyclic π-π conjugated homocompounds and n-π conjugated heterocompounds. It also occurs for some heterocycles, for which the n-electrons of the endo heteroatom(s) are conjugated with the π-electrons of the ring(s). When σ-π hyperconjugation is mixed with π-π and/or n-π conjugation, weak electron delocalization is possible for cyclic and acyclic derivatives. 

Among various quantitative descriptors of electron delocalization proposed in the literature to verify the aromaticity of π-electron cyclic systems (structural, electronic, energetic, and magnetic parameters; see in ref. [2]), the geometry-based HOMED index [67,68], recently extended to cyclic and acyclic heterocompounds, properly determines any type of electron delocalization, σ-π hyperconjugation, n-π and π-π conjugation, aromaticity, and their mixtures. The HOMED procedure is based on the original harmonic oscillator model of aromaticity (HOMA) idea published in the 1970s by Kruszewski and Krygowski [69,70]. According to this procedure, the computed structures, particularly their bond lengths, can be used in the HOMED estimation. 

The HOMED index can be calculated for the entire molecule as well as for its fragment using an equation analogous to that proposed earlier for the HOMA index [71]: HOMED (or HOMA) = 1 − {α(CC)Σ[*R*_o_(CC) − *R*_i_(CC)]^2^ + α(CN)Σ[*R*_o_(CN) − *R*_i_(CN)]^2^ + α(CO)Σ[*R*_o_(CO) − *R*_i_(CO)]^2^}/*n*. In this equation, α(CC), α(CN), and α(CO) are the normalization constants, different for CC, CN, and CO bonds; *R*_o_(CC), *R*_o_(CN), and *R*_o_(CO) are the optimum bond lengths, also different for the full delocalized system containing only CC, CN, or CO bonds; *R*_i_(CC), *R*_i_(CN), and *R*_i_(CO) are the calculated bond lengths in the investigated system; and *n* is the number of bonds taken into account in the HOMED (or HOMA) estimation. 

For the entire molecule or its fragment containing an even number of bonds (*n* = 2*i*), i.e., *i* single and *i* double bonds, the normalization α constants can be determined according to the equation proposed for the HOMA index [67,68,71]: α = 2[(*R*_o_ − *R*_s_)^2^ + (*R*_o_ − *R*_d_)^2^]^−1^, where *R*_s_ and *R*_d_ are the single and double bond lengths in compounds selected for the reference molecules. For the systems possessing an odd number of bonds (*n* = 2*i* + 1), i.e., *i* + 1 single bonds and *i* double bonds, the normalization α constants can be estimated by using the modified equation [67,68]: α = (2*i* + 1)[(*i* + 1)(*R*_o_ − *R*_s_)^2^ + *i*(*R*_o_ − *R*_d_)^2^]^−1^. 

The HOMED parameters *R*_s_, *R*_d_, and *R*_o_ can be calculated for the reference molecules at the same level of theory as *R*_i_ for the investigated derivatives. Their values (in Å) calculated at the DFT(B3LYP)/6-311+G(d,p) level and applied in the HOMED procedure are as follows: 1.5300 (H_3_C–CH_3_), 1.3288 (H_2_C=CH_2_), and 1.3943 (benzene) for CC bonds; 1.4658 (H_3_C–NH_2_), 1.2670 (H_2_C=NH), and 1.3342 (1,3,5-triazine) for CN bonds; and 1.4238 (H_3_C–OH), 1.2017 (H_2_C=O), and 1.2811 (protonated carbonic acid) for CO bonds [67,68]. On the basis of these *R* values, the normalization α constants equal to 88.09, 91.60, and 75.0 for CC, CN, and CO bonds, respectively, can be used for systems with an even number of bonds, whereas, for systems with an odd number of bonds, the following α constants can be applied: 80.90, 84.52, and 69.74 for CC, CN, and CO bonds, respectively [67,68]. Note that, in the original HOMA procedure, one α constant has been employed for all types of bonds, CC, CX, and XY [69,70]. Its value (98.89) is not very different from those used in the HOMED procedure.

The HOMED descriptor is equal to unity for the benzene-structure calculated at the DFT(B3LYP)/6-311+G(d,p) level. It is equal to zero for the structure of the hypothetical cyclohexa-1,3,5-triene with localized single and double bond lengths equal to those in the DFT-calculated structures of H_3_C–CH_3_ and H_2_C=CH_2_ [67,68]. This normalization of the geometry-based index is analogous to that for the original HOMA index, which is close to unity for benzene, and equal to zero for the hypothetical cyclohexa-1,3,5-triene with localized single and double bond lengths equal to those in the experimental structures of H_3_C–CH_3_ and H_2_C=CH_2_ [69,70]. 

Quite a different situation takes place for the HOMA index reformulated in 1993 by Krygowski [71]. The normalization α constants strongly differ from those of the original HOMA and modified HOMED indices, because the reference molecules of different instances of electron delocalization power have been selected for single and double bonds. For example, the experimental structures of moderately delocalized 1,3-butadiene (H_2_C=CH–CH=CH_2_) and monomeric formic acid (HO–CH=O) have been applied for the reference single and double CC and CO bonds, whereas those of slightly delocalized H_3_C–NH_2_ and H_2_C=NH have been employed for the reference single and double CN bonds, respectively. Although the reformulated HOMA index is close to unity for benzene like the original HOMA and modified HOMED indices, it is not equal to zero for the cyclohexa-1,3,5-triene with single and double bond lengths equal to those in H_3_C–CH_3_ and H_2_C=CH_2_, respectively, but to those in medium delocalized H_2_C=CH–CH=CH_2_. The use of a different measure for CC, CN and CO bond lengths in the reformulated HOMA procedure leads to completely different normalization α constants, exceptionally high for CC (255.7), medium for CO (157.38), and small for CN (93.52). These parameters applied to the reformulated HOMA index reveal significant differences between the reformulated and original HOMA scales, and also between the reformulated HOMA and modified HOMED scales. These differences have been discussed in detail in our previous works [35,65,67,68]. For our application to the title compounds, we selected the following most important difference between the reformulated HOMA and modified HOMED indices: The HOMED index properly describes electron delocalization in any conjugated system. It can be applied for cyclic and acyclic π-electron molecules, such as well delocalized aromatic derivatives, as well as less delocalized π-π, n-π, and σ-π systems, whereas the HOMA descriptor can be applied only for aromatic compounds or for derivatives containing only the same type of bonds, e.g., only CC, only CN, or only CO [35,65,67,68]. 

Since tautomeric mixtures of pyrimidine bases and their model compounds contain differently conjugated tautomers, we chose the HOMED index for the quantitative description of electron delocalization which is always in relation with tautomeric conversion(s). To minimize computational errors in these kinds of investigations, the level of theory selected for the HOMED estimations is the same as that for the calculations of Δ*G*s. The DFT(B3LYP)/6-311+G(d,p) level has been employed for both geometry-optimization and thermochemistry-calculation. For the HOMED determinations, we used the DFT-calculated bond lengths of the title derivatives [18,22,28,49,58,59,60,61,62,63,64,65]. The HOMED values estimated for the neutral and ionized forms are included in Tables S4–S6 (Supplementary Materials).

## 5. Complete Prototropic Mixtures for Title Compounds

Before the analysis of the prototropic conversions for nucleic acid pyrimidine bases and their model compounds, first, it is important to explain how we consider all tautomeric mixtures. In this review, we choose aromatic isomers containing tautomeric proton(s) at exo group(s) as reference tautomers for other less delocalized forms, like for phenol and aniline. Although the aromatic isomers are not always favored for investigated heterocompounds, this kind of treatment makes it possible to distinguish between some internal effects. For example, we can analyze the internal effects of endo N atom(s) on the stability of individual isomers, composition of isomeric mixtures, and tautomeric preferences. We can also estimate the internal effects of additional exo groups (OH and NH_2_) when proceeding from monosubstituted model compounds to disubstituted pyrimidine bases. 

### 5.1. Monosubstituted Azines 

Model compounds of pyrimidine bases, such as mono-hydroxy and mono-amino aromatic azines, contain the six-membered ring with endo N atom(s) and one exo group, OH or NH_2_, respectively. From a structural point of view, model azines can be considered as aza derivatives of phenol and aniline, respectively [48,49,58,59,60,61]. Thus, the scheme of intramolecular proton-transfers in azines is analogous to that in the parent compounds (Figure 1). Independently on the number of endo N atoms, the labile proton can move according to the 1,3- 1,5-, or 1,7-proton shift from the exo group to the conjugated ring-site at the 2-, 4-, or 6-position vis-à-vis OH or NH_2_. Consequently, four tautomers (**a**–**d**) can be distinguished for azines, one hydroxy or one amino form (**a**), and three oxo or three imino forms (**b**–**d**), respectively. They differ by the positions of the tautomeric proton and π-electrons. When the N atom is located in the ring of the model compound at the site conjugated with the exo group, it directly participates in prototropy. We can distinguish between various tautomeric equilibria given in Table 1. In the literature, different names or abbreviations are used for tautomers, such as hydroxy and oxo, amino and imino, or OH, NH, and CH forms. Only the last abbreviations clearly show to which atom (O, N, or C) the labile proton is attached in a particular tautomer. 

**Figure 2:**
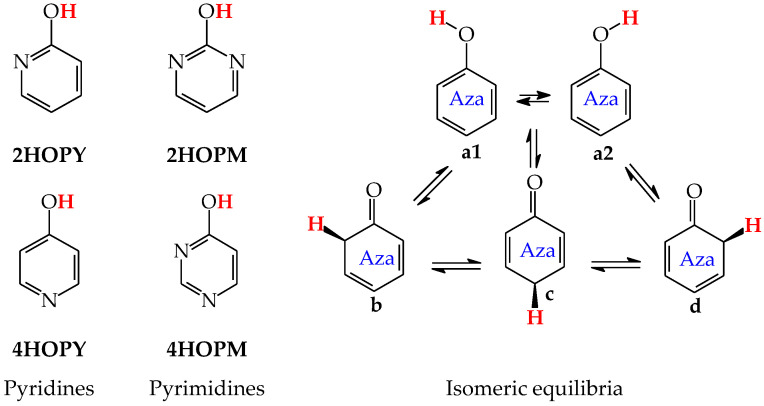
Isomeric equilibria possible for 2- (**2HOPY**) and 4-hydroxypyridines (**4HOPY**), and 2- (**2HOPM**) and 4-hydroxypyrimidines (**4HOPM**). The labile proton shown in bold red color. Tautomeric equilibria and rotation about C–OH indicated by ⇌ and ⇄ arrows, respectively.

Except for tautomeric conversions, OH tautomers of mono-hydroxy azines exhibit additionally conformational isomerism about the C–OH single bond (Figure 2) [48,49,72]. The possibility of OH-rotation leads to two extreme positions of the hydroxy H atom (*syn*- and *antiperiplanar*) vis-à-vis neighboring atoms or groups and to two extreme conformational isomers (**1** and **2**) of OH tautomer (**a**). Consequently, five isomers (tautomers–rotamers) can be distinguished for mono-hydroxy azines. For unsymmetrically substituted 2-hydroxypyridine (**2HOPY**) and 4-hydroxypyrimidine (**4HOPM**), the five tautomers–rotamers possess different constitutions, whereas for symmetrically substituted 4-hydroxypyridine (**4HOPY**) and 2-hydroxypyrimidine (**2HOPM**), the isomers **a1** and **a2**, and also **b** and **d,** have identical structures. Thus, five and three tautomers–rotamers, respectively, can be considered in quantum-chemical calculations. The OH-rotamers **a1** and **a2** of **2HOPY** and **4HOPM** differ by intramolecular interactions with neighboring atoms or groups (see in Section 6). Consequently, they display different stabilities, different relative energies, and different percentage contents in the isomeric mixtures.

**Figure 3:**
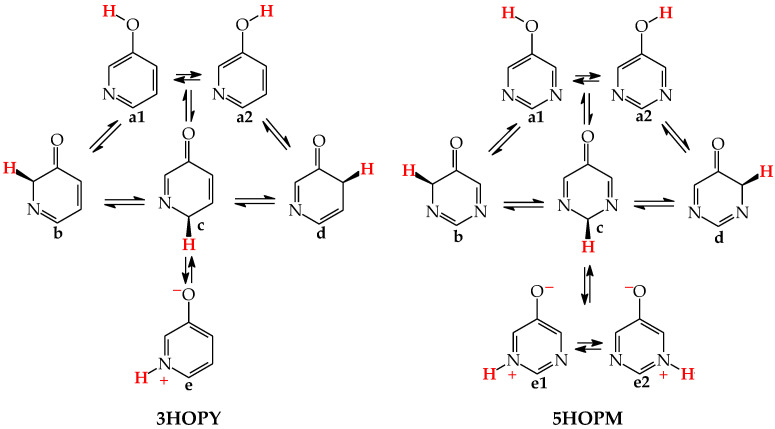
Isomeric equilibria possible for 3-hydroxypyridine (**3HOPY**) and 5-hydroxypyrimidine (**5HOPM**). The labile proton shown in bold red color. Tautomeric conversions indicated by ⇌ arrows, while zwitterion formation and rotation about C–OH by ⇄ arrows.

In an aqueous solution, derivatives possessing the endo N atoms at the 3- and 3,5-positions vis-à-vis the exo OH group (**3HOPY** and **5HOPM** in Figure 3) can additionally form one (**e**) and two (**e1** and **e2**) zwitterionic forms, respectively [73]. It is important to mention here that the isomers **e**, **e1**, and **e2** are exceptionally rare forms in the gas phase and cannot be identified experimentally [74], similar to the zwitterionic forms of amino acids [75]. Moreover, they cannot be considered as prototropic tautomers. Intramolecular proton-transfers from O to N and vice versa cannot be treated as prototropic conversions [1,2,3,73]. The endo N atoms in **3HOPY** and **5HOPM** are not conjugated with the exo OH group, and the intramolecular proton-transfers from O to N are not accompanied by the migration of π-electrons. The 1,4- and 1,6-proton-shifts in **3HOPY** and **5HOPM** are simple intramolecular proton-transfers—internal neutralization reactions, analogous to those for amino acids. These proton-transfers lead to the separation of the positive and negative charge in the zwitterionic forms **e**, **e1**, and **e2**. However, one can find numerous documents in the literature in which zwitterions and intramolecular neutralization processes have been confused with prototropic tautomers and prototropy, respectively. 

The zwitterionic forms of **3HOPY** and **5HOPM** have been examined in an aqueous solution and their presence confirmed in experimental UV spectra in 1970 by Kwiatkowski, who applied the Pariser–Parr–Pople calculations for the interpretation of the electronic absorption spectra [73]. However, in a recent article on interesting resonant inelastic X-ray scattering experiments carried out for **3HOPY** in an aqueous solution, the authors not only considered the zwitterionic structure **e** as a prototropic tautomer, but also inappropriately used the name ‘keto tautomer’ for **e** [74]. In the isomeric mixture of **3HOPY** (Figure 3), only the structures **b**–**d** refer to the keto forms (CH tautomers), like keto forms of phenol (Figure 1), in which the labile proton is attached to the endo C atoms at the 2-, 4-, and 6-positions vis-à-vis OH. The same is true for isomers of **5HOPM**. The two derivatives, **3HOPY** and **5HOPM,** do not belong to the family of tautomeric aromatic azines. From a prototropy phenomenon point of view, they can be classified in the family of tautomeric phenols. The tautomeric mixture of each of them contains four tautomers, one enol (OH rotamers **a1** and **a2**), and three keto tautomers (CH forms **b**–**d**). The endo N atoms do not participate directly in tautomeric conversions. In the gas phase, the zwitterionic forms of **3HOPY** and **5HOPM** can be neglected [74]. 

**Figure 4:**
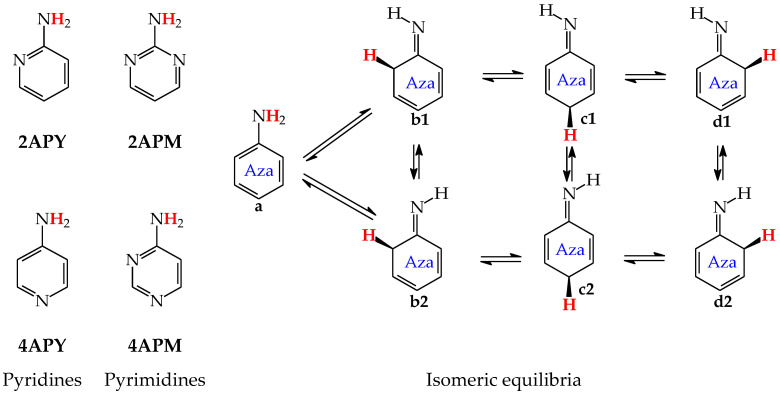
Isomeric equilibria for 2- (**2APY**) and 4-aminopyridines (**4APY**), and 2- (**2APM**) and 4-aminopyrimidines (**4APM**). The labile proton shown in bold red color. Tautomeric equilibria and configurational isomerism about =NH indicated by ⇌ and ⇄ arrows, respectively.

For mono-amino azines (Figure 4), except for prototropic tautomers, we can distinguish, additionally, configurational isomers about the C=NH double bond in the imino forms **b**–**d**. This means that at least seven isomers are possible for 2- (**2APY**) and 4-aminopyridines (**4APY**), and also for 2- (**2APM**) and 4-aminopyrimidines (**4APM**) [58,59,60,61]. For unsymmetrically substituted **2APY** and **4APM**, the seven isomers possess different constitutions. Intramolecular interactions of the exo = NH group with neighboring endo atoms or groups (N/NH or CH/CH_2_) possible in the imino forms influence the stability of configurational isomers (**1** and **2**), and their relative energies and amounts in the isomeric mixtures (see in Section 6). On the other hand, the corresponding isomers **b1** and **d2**, **b2** and **d1**, and also **c1** and **c2** have identical structures for symmetrically substituted **4APY** and **2APM**. Hence, in quantum-chemical calculations, seven and four isomers can be considered for unsymmetrically and symmetrically substituted amino azines, respectively.

Derivatives of amino azines possessing N atoms at the 3- and 3,5-positions in the ring vis-à-vis the exo NH_2_ group do not belong to the family of tautomeric azines. Being unconjugated with NH_2_, the endo N atoms do not participate in prototropic conversions. Any intramolecular proton-transfer from the exo NH_2_ to the endo N atoms can only lead to the zwitterionic form **e**. Then, 3-Aminopyridine (**3APY**) and 5-aminopyrimidine (**5APM**) can be classified in the family of the tautomeric parent system aniline, in which the tautomeric proton can only be transferred from the exo NH_2_ group to endo C atoms at the 2-, 4-, or 6-position vis-à-vis NH_2_ together with the migration of π-electrons. Since the exo NH_2_ group in arenes is a considerably less acidic function in an aqueous solution than OH, the zwitterion formation in **3APY** and **5APM** is less probable than in **3HOPY** and **5HOPM**. We have found no documents in the literature on the zwitterionic forms of **3APY** and **5APM**.

### 5.2. Disubstituted Pyrimidine Bases

**Figure 5:**
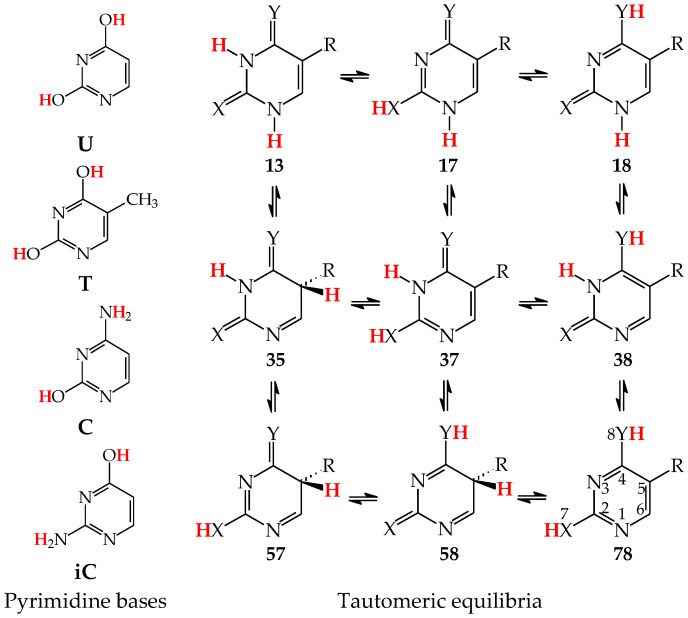
Tautomeric equilibria in pyrimidine bases: uracil (**U** with X, Y = O, R = H), thymine (**T** with X, Y = O, R = CH_3_), cytosine (**C** with X = O, Y = NH, R = H), and isocytosine (**iC** with X = NH, Y = O, R = H). The two labile protons indicated in bold red color. The number of atoms shown in **78**.

Similar to model azines, disubstituted pyrimidine bases (**U**, **C**, **T**, and **iC**) belong to the family of tautomeric heterocycles with the six-membered ring [2,4,5]. Two N atoms are included in the ring at the 1- and 3-positions, and two exo heterogroups are attached to the endo C atoms at the 2- and 4-positions (Figure 5). The aromatic forms of **U** and **T** possess two exo OH groups, whereas those of **C** and **iC** contain one OH and one NH_2_ group. Thymine (**T**) is a 5-methyl derivative of **U**, and **iC** is a constitutional isomer of **C**. The exo OH and NH_2_ groups in **C** are placed at the 2- and 4-positions, respectively, whereas those in **iC** are attached to the reverse positions (4 and 2). The three pyrimidine bases **U**, **T**, and **C** are well-known nucleic acid bases. Their intermolecular interactions with purine bases (**U** with **A** in RNA, **T** with **A** in DNA, and **C** with **G** in RNA and DNA) determine the conformational structure of nucleic acids. Although **iC** cannot be classified in the family of nucleic acid bases, it is a structural part of **G**. The endo and exo functional groups in the **iC** part of **G** are directly engaged in interactions of **G** with **C** in nucleic acids. Consequently, the **iC** part of **G** is also responsible for the conformational structure of nucleic acids. 

Pyrimidine bases are more complex tautomeric systems than mono-substituted azines. Model azines possess one labile proton and four conjugated sites, like phenol and aniline, whereas disubstituted pyrimidine bases contain two labile protons and five conjugated sites (two heteroatoms in exo groups, X7 and Y8, and three endo atoms, N1, N3 and C5). However, the scheme of intramolecular proton-transfers for each labile proton in pyrimidine bases is analogous to that in model compounds. The labile protons can move from the exo groups to the corresponding conjugated sites according to the 1,3- 1,5-, or 1,7-proton shift, and vice versa. Consequently, all tautomeric proton-transfers lead to the mixture of nine tautomers (Figure 5) [48,62,64,65]. 

Although the number of tautomers in pyrimidine bases is higher than that for model compounds, it results from analogous tautomeric conversions given in Table 1. In tautomeric mixtures of pyrimidine bases, we can distinguish the OHOH, NHOH, NHNH, CHNH, and CHOH tautomers for **U** and **T**, and the NHOH, NHNH, CHNH and CHOH forms for **C** and **iC**. To simplify and generalize the names of these nine tautomers for pyrimidine bases, we applied the following abbreviations here: **13**, **17**, **18**, **35**, **37**, **38**, **57**, **58**, and **78**. They refer to the numbers of two atoms which attach the two tautomeric protons. For example, the abbreviations **13** and **78** indicate two tautomers of pyrimidine base (**U**, **T**, **C**, or **iC**), one containing the labile protons at endo N atoms (N1 and N3), and the other one possessing the labile protons at the exo heteroatoms (X7 and Y8).

Three tautomers (**35**, **57**, and **58**) containing one labile proton at C5 have been usually neglected by chemists even in theoretical investigations on the electron affinity of pyrimidine bases. However, these tautomers play a very important role in ionization processes, particularly when nucleic acid bases gain one excess electron [28,57,63,64,76]. For this reason, all nine possible tautomers have to be analyzed for tautomeric bases to obtain the complete picture on their structures and properties in different isolated states. Investigations performed for selected tautomers, i.e., favored (or canonical) forms detected for neutral bases, lead only to partial results.

**Figure 6:**
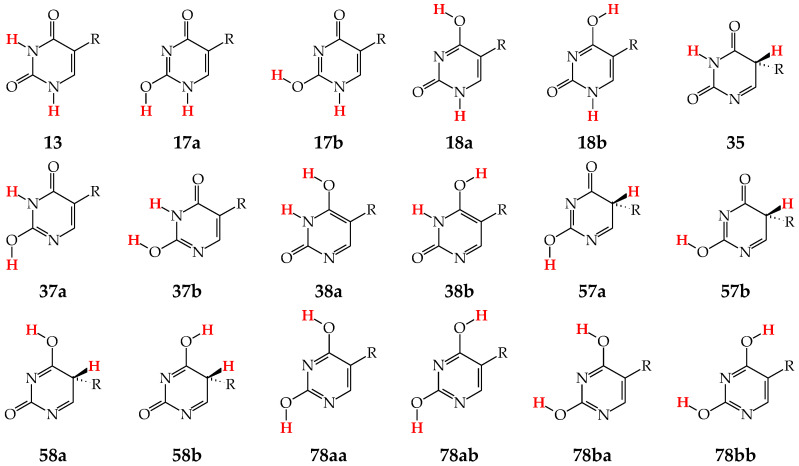
Eighteen tautomers-rotamers possible for **U** (R = H) and **T** (R = CH_3_). The two labile protons shown in bold red color. Abbreviations of tautomers refer to the number of atoms possessing the two labile protons and those of rotamers to the two extreme positions of the exo hydroxy H atom at 2- (**a** or **b**) and/or 4-position (**a** or **b**).

**Figure 7:**
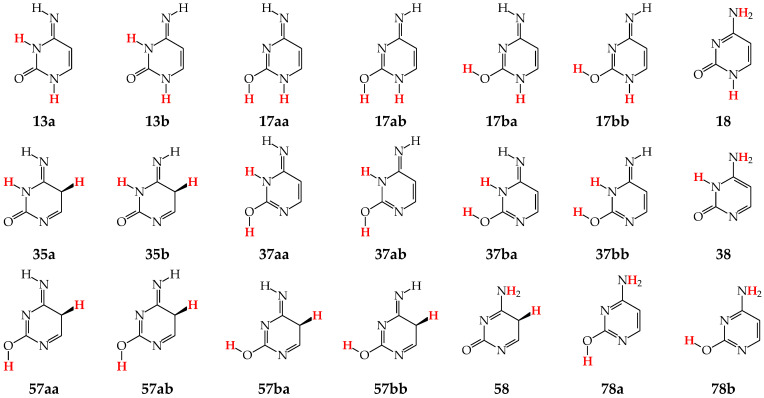
Twenty-one tautomers-rotamers possible for **C**. The two labile protons indicated in bold red color. Abbreviations of tautomers refer to the number of atoms possessing the labile protons and/or those of rotamers to the two extreme positions of the exo hydroxy H atom at 2-position (**a** or **b**) and/or the imino H atom at 4-position (**a** or **b**).

Tautomers of pyrimidine bases possessing the exo –OH and =NH groups display, additionally, two types of isomerism, conformational isomerism about the C–OH single bond and configurational isomerism about the C=NH double bond, respectively, like those of model azines (Figure 2 and Figure 4). The H atoms of these groups can take two extreme positions (*syn*- and *antiperiplanar*) vis-à-vis the neighboring endo atoms or groups (N/NH or CH/CH_2_) and change the stability of isomers due to differences in their intramolecular interactions (see Section 6). In consequence, eighteen tautomers-rotamers can be distinguished for **U** and **T** (Figure 6) [48,62,63], and twenty-one isomers for **C** (Figure 7) [64] and **iC** (Figure S1, Supplementary Materials) [28,57,65]. Although the tautomeric mixtures of **C** and **iC** consist of the same number of tautomers–rotamers (twenty-one), their structures cannot be shown in one common figure, like for **U** and **T**. The two exo groups (OH and NH_2_) in **C** and **iC** are placed at different positions, and, thus, the tautomeric structures of **13**, **17**, **18**, **35**, **37**, **38**, **57**, **58**, and **78** are different. Tautomers-rotamers of **C** and **iC** should be presented separately [64,65].

The complete isomeric mixtures for neutral **U**, **C**, and **iC** have been investigated in vacuo by quantum-chemical computations in the last twenty years [48,62,65,67]. Unfortunately, for neutral thymine, the complete isomeric mixture, including C5H tautomers-rotamers, has not yet been considered even in the recent theoretical-computational study of Stasyuk et al. [77], who investigated only five **T** isomers. Assuming weak polarizability, and inductive and steric effects of CH_3_ on tautomeric equilibria in **T**, the stability order for the five investigated **T** isomers can be similar to that of **U** forms. Indeed, these orders (**T13** > **T37a** > **T18a** > **T78aa** > **T78ba** [77] and **U13** > **U37a** > **U18a** > **U78aa** > **U78ba** [62]) are analogous. The DFT-calculated relative energies for the five isomers of **T** and **U** are almost the same. Some discrepancies, not larger than 1 kcal mol^−^^1^, can be a consequence of the total CH_3_-substituent effect on the stability of **T** isomers. Note that the exo CH_3_ group in **T** is not hyperconjugated with functional endo and exo groups. The possible transfer of H^+^ (or H^●^) from CH_3_ to C2, C4, or C6 can be neglected for the neutral base. Nevertheless, the complete isomeric mixture for neutral **T** should be investigated in the future.

## 6. Internal Effects of Functional Groups on Isomer-Stability

Although pyrimidine nucleic acid bases are structurally similar and display an analogous scheme to prototropic conversions (Figure 5), their isomeric preferences are not the same in vacuo. The orders of isomer-stabilities, measured by means of the relative thermochemical parameters (Δ*E*, Δ*H*, Δ*G*, or *K* at 298.15 K), are completely different for their neutral forms [57,62,64,65]. These differences result mainly from the different internal effects of functional groups. When proceeding from the parent systems, phenol and aniline, to mono-hydroxy and mono-amino aromatic azines, the structural replacement of CH group(s) by N atom(s) at the 2-, 4-, and/or 6-position(s) in the ring strongly influences the thermochemical parameters of individual isomers (**a**–**d**) and their percentage contents in isomeric-mixtures [18,22,49,58,59,60]. Additionally, when going from monosubstituted aromatic azines to disubstituted pyrimidine bases, the structural introduction of the second exo group induces successive variations in internal effects, relative stabilities, and isomeric preferences [49,58,59,60,62,64,65]. 

The internal effects of functional groups can be analyzed on the basis of Δ*G*s computed at the same level of theory for individual isomers of disubstituted pyrimidine bases, monosubstituted azines, and their parent compounds. Depending on isomeric conversions (conformational or configurational isomerism, and/or tautomeric rearrangement), the structurally introduced endo N atom in azines influences the isomer stability in a different way. Generally, we can distinguish between three types of N-endo effects originating from: (i) intramolecular interactions between endo and exo groups in conformational and configurational isomers, (ii) the transmission of N-endo electronic-effects to tautomeric conjugated sites, and (iii) the direct participation of the endo N atom in prototropic conversions. Taking Δ*G*s into account, we can also analyze the internal effects of the additional exo OH or NH_2_ group when going from monosubstituted aromatic azines to disubstituted pyrimidine bases.

### 6.1. Intramolecular Interactions between Endo and Exo Groups 

**Figure 8:**
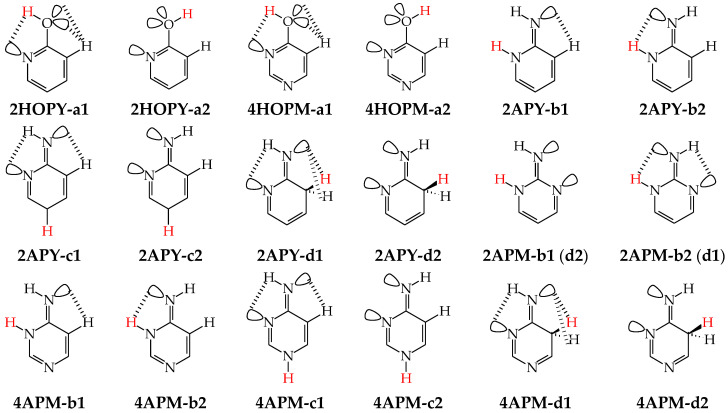
Intramolecular interactions between neighboring exo and endo groups/atoms in selected conformational and configurational isomers of model azines. The labile proton indicated in red color, and H atoms engaged in intramolecular interactions in black color.

Intramolecular interactions of endo N atom(s) with exo group(s) vary from favorable in one isomer to unfavorable in the other one, and also from more favorable (or more unfavorable) in one isomer to less favorable (or less unfavorable) in the other one. For conformational isomers of mono-hydroxy aromatic azines (**a1** and **a2** in Figure 2), these effects strongly influence their stability. When proceeding from **a1** to **a2** in unsymmetrically substituted hydroxy azines (**2HOPY** and **4HOPM**), the Δ*G* values differ by ca. 5 kcal mol^−^^1^ at various levels of theory, AM1, DFT, MP2, G2, and G2(MP2) (for an explanation of theoretical-method abbreviations, see in Supplementary Materials) [48,49,72]. This effect refers to the difference between the favorable and unfavorable interactions of the exo OH with neighboring endo groups (Figure 8). 

The favorable interactions are possible in **a1** between the hydroxy H atom and the lone electron-pair of the endo N atom, and also between the H atom of the endo CH group and two lone electron-pairs of the exo O atom. The unfavorable repulsions occur in **a2** between the exo OH and endo CH groups, and also between the lone electron-pairs of the exo O and endo N atoms. Less or more favorable or unfavorable intramolecular interactions can be distinguished for configurational isomers of mono-amino aromatic azines (**b1** and **b2**, **c1** and **c2**, and, also, **d1** and **d2** presented in Figure 4). The differences between Δ*G*s for these isomer-pairs vary from 3–5 for **2APY** and **4APM** to even 7 kcal mol^−^^1^ for **2APM** at the DFT level {B3LYP/6-311+G(d,p)} [58,59,60]. For selected isomers of **2APY**, **4APM**, and **2APM**, the favorable intramolecular interactions between the lone electron-pairs of heteroatoms and the H atom(s) of the CH or NH group, as well as the unfavorable repulsions between the CH and NH groups and/or between the lone electron-pairs of heteroatoms, are shown in Figure 8.

For pyrimidine nucleic acid bases (**U**, **C**, and **iC**), we can also distinguish between the N-endo effects resulting from favorable and/or unfavorable intramolecular interactions between neighboring exo and endo groups when going from one rotamer to the other one of the same tautomer. For example, the hydroxy or imino H atom of the exo group can favorably interact with the lone electron pair of the neighboring heteroatom in one isomer, whereas repulsions of the neighboring lone electron pairs or H atoms in the other isomer lead to unfavorable interactions. Comparing Δ*G*s calculated for conformational isomers about the C–OH single bond [62,64,65], we can estimate the N-endo effects on the stabilities of rotamers containing –OH at the 2- and/or 4-position in **U**, **C**, and **iC**. Separately, comparing the Δ*G*s of configurational isomers about the C=NH double bond [64,65], we can calculate the N-endo effects on the stabilities of isomers possessing =NH at the 2- and 4-position in **iC** and **C**, respectively. 

Table 2 presents the absolute values of Δ*G* differences {δ*G* = Δ*G*(**a**) − Δ*G*(**b**), referred to N-endo effects} calculated at the same DFT level for the conformational and configurational isomers **a** and **b** having two extreme positions for H atoms of the –OH and =NH groups in **U**, **C**, and **iC**. Interestingly, there is some analogy of the N-endo effects in pyrimidine bases. For –OH at the 2-position, the N-endo effects in the tautomers-rotamers of **U** are close to those of the corresponding **C** isomers, whereas, for –OH at the 4-position, they are close to those of the corresponding **iC** isomers. For the 5-methyl derivative of uracil (**T**), one can find in the literature numerous articles on prototropy. However, the authors limited their computations to a few isomers, mainly major and minor forms with favorable intramolecular interactions. Hence, general estimations of the N-endo effects in **T** isomers cannot be made here, even on the basis of recent high-level DFT-computational data [77]. We can only assume that they can be analogous to those for **U**.

**Figure 9:**
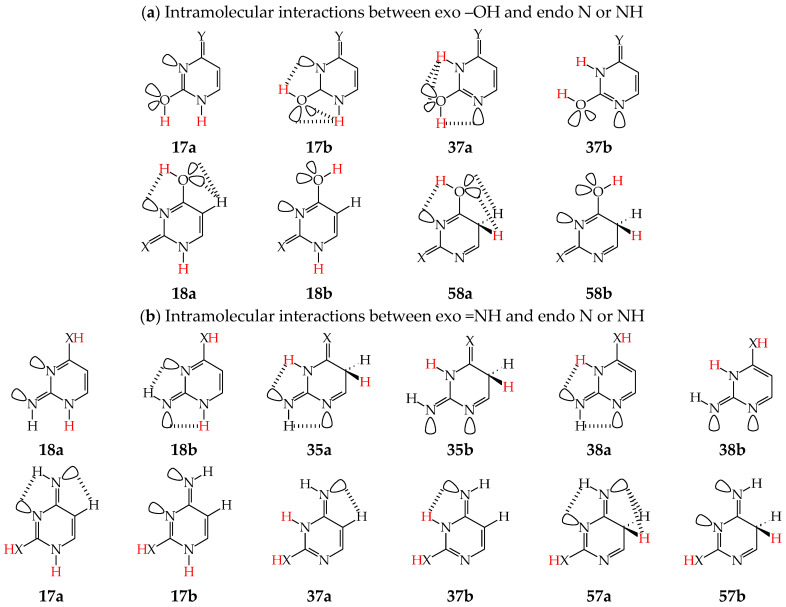
Intramolecular interactions between neighboring exo and endo groups/atoms in selected conformational (**a**) and configurational (**b**) isomers of pyrimidine bases (X, Y: O or NH). The labile protons indicated in red color and H atoms engaged in intramolecular interactions in black color.

When favorable or unfavorable intramolecular interactions in the two isomers **a** and **b** of pyrimidine-base tautomers (**U57**, **U78**, **C57**, and **C78**) do not change very much, the N-endo effects are close to zero (<2 kcal mol^−^^1^). However, when these interactions vary from favorable in one isomer to unfavorable in the other one, these effects increase even to 7–10 kcal mol^−^^1^ for –OH at the 2-position in rotamers of **U37**, **U17**, **C37**, and **C17**. In these rotamers, two very favorable intramolecular interactions are possible in one form between the hydroxy H atom and the lone electron-pair of the neighboring endo N atom, and also between the H atom of the endo NH group and two lone electron-pairs of the exo O atom (Figure 9). In the other form, two very unfavorable repulsions take place between the exo OH and endo NH groups, and also between the lone electron-pairs of the exo O and endo N atoms. For –OH at the 4-position, the N-endo effects do not exceed 6–8 kcal mol^−^^1^ in rotamers of **U58**, **U18**, **iC58**, and **iC18**. The favorable and unfavorable intramolecular interactions of –OH at the 4-position (Figure 9) are analogous to those for **4HOPM** (Figure 8). In the case of configurational isomers about C=NH, the N-endo effects are slightly stronger for the exo group at the 2- (<8 kcal mol^−^^1^) compared to the 4-position (≤5 kcal mol^−^^1^). Stronger effects occur for tautomers for which the configuration-change about C=NH leads to greater differences in the favorable and unfavorable interactions between the exo and endo groups.

### 6.2. Transmission of N-Endo Effects to Tautomeric Sites

Except possible intramolecular interactions with exo groups, the endo N atom(s) can also act as transmitter(s) of electronic effects to other tautomeric sites, and change the keto-enol equilibria in mono-hydroxy aromatic azines or imine-enamine conversions in mono-amino derivatives. In the other words, the endo N atom(s) can influence the proton-transfers from the exo OH or NH_2_ group to the endo C atom(s) at the 2-, 4- and/or 6-position vis-à-vis the exo group and change the relative stabilities of **a**–**d** (Figure 2 and Figure 4). The N-endo transmitter-effects {δ*G* = Δ*G*(model-azine isomer) − Δ*G*(parent-compound isomer)} can be estimated as the difference between Δ*G*s calculated for the corresponding CH isomers of mono-hydroxy (**2HOPY**, **4HOPY**, **2HOPM**, and **4HOPM**) or mono-amino (**2APY**, **4APY**, **2APM**, and **4APM**) derivatives [49,58,59,60], and those for the analogous CH forms of phenol (PhOH) [18] or aniline (PhNH_2_) [22], respectively. Note that, according to our treatment of tautomeric mixtures, the hydroxy and amino isomers of the parent compounds and model azines (structures **a** with favorable intramolecular interactions in unsymmetrically substituted derivatives) play a role as reference tautomers (Δ*G* = 0 kcal mol^−^^1^). 

Generally, when proceeding from phenol to mono-hydroxy azines and from aniline to mono-amino azines, the structural replacement of the CH group(s) by the N atom(s) in the six-membered ring increases the Δ*G*s of the CH tautomers-rotamers. This means that the N-endo atom(s), acting as electron-accepting group(s), reduces the basicity of the endo tautomeric C atoms, decrease the probability of H-attachment, and diminish the stability of CH isomers. Consequently, they become more unlikely forms for the model azines than for the parent systems. This means that the proton-transfers from the exo OH to the endo C atoms (enol → keto conversions) and from the exo NH_2_ to the endo C atoms (enamine → imine conversions) are less probable for hydroxy and amino azines than for phenol and aniline. Table 3 illustrates variations of the N-endo effects (δ*G*s) in model azines. For δ*G* estimations, the Δ*G* values, calculated at the same level of theory {B3LYP/6-311+G(d,p)}, have been employed for analogous CH tautomers of the parent compounds and model azines. 

In the case of mono-hydroxy azines, the Δ*G* values of CH isomers increase by ca. 1 and 7 kcal mol^−1^ for **4HOPY** and **2HOPY**, respectively, and by ca. 8 kcal mol^−^^1^ for **4HOPM** and **2HOPM** [18,49]. This suggests that the effects of the endo N atom at the 2-position vis-à-vis the exo group for **2HOPY** are stronger than those at the 4-position for **4HOPY**. The N-endo effects seem to be additive for **4HOPM** (1 + 7 = 8 kcal mol^−1^). However, the total effects of two endo N atoms at the 2- and 6-positions vis-à-vis the exo group in **2HOPM** are smaller than the sum of the partial effects of the single N atom in **2HOPY** (8 < 14 kcal mol^−1^). This discrepancy can be explained by the differences in intramolecular interactions in the hydroxy forms of **2HOPY** and **2HOPM** (Figure 8), used as the reference tautomers. These interactions are included in the N-endo transmitter-effects. The same is true for mono-amino-derivatives. The Δ*G* values of CH isomers are higher for pyridine and pyrimidine derivatives than for unsubstituted aniline by 1–10 kcal mol^−1^ [22,58,59,60]. The N-endo effects for the pyridine derivative **2APY** (2–7 kcal mol^−1^) are stronger than those for **4APY** (1–2 kcal mol^−^^1^), but they are weaker than those for the pyrimidine derivatives **2APM** (10 kcal mol^−^^1^) and **4APM** (4–9 kcal mol^−^^1^). Some differences between the δ*G*s for the isomers **1** and **2** can result from the differences in intramolecular interactions for the imine forms (Figure 8). Generally, more endo N atoms in aromatic azines mean stronger total N-endo transmitter-effects.

### 6.3. Effects of Additional Exo OH and NH_2_ Groups

The exo OH and NH_2_ groups in aromatic systems in vacuo display various total electron-donating effects. In disubstituted pyrimidine bases and monosubstituted model azines, the exo groups can additionally interact with endo neighboring groups or atoms. Being conjugated with some C atoms, the exo OH and NH_2_ groups can increase its basicity and its ability to perform proton binding [78]. When proceeding from mono-hydroxy and mono-amino pyrimidines (**2HOPM**, **4HOPM**, **2APM**, and/or **4APM**) to **U**, **C**, and **iC**, the electron-donating effects of the additional exo group add to the electron-accepting effects of N-endo. The two opposite effects of the exo and endo groups lead to the total effects of the functional groups. The negative δ*G* values {δ*G* = Δ*G*(pyrimidine-base isomer) − Δ*G*(model-azine isomer)}, calculated on the basis of DFT-data for C5H tautomers with favorable intramolecular interactions [49,59,60,62,64,65] (Table 4), suggest that the electron-donating effects of the exo groups are stronger than the electron-accepting effects of the endo N atoms. The total effects of the exo and endo groups in pyrimidine bases increase the probability of binding the labile proton to C5, and, consequently, increase (by 2–8 kcal mol^−^^1^) the stability of the C5H isomers of **U**, **C**, and **iC**, measured by means of δ*G*. In **C** and **iC**, the total effects of the additional exo NH_2_ group seem to be stronger than those of the additional exo OH group by 2.4 kcal mol^−^^1^. The weakest total effect takes place for 2-OH in **C** and the strongest one occurs for 2-NH_2_ in **iC**. For uracil, the total effects of 2-OH and 4-OH are very similar. They differ by only 0.3 kcal mol^−^^1^. 

Changes of the relative Gibbs energies for more stable isomers of the N1H and N3H tautomers can be estimated in an analogous way as those for the C5H tautomers: δ*G* = Δ*G*(pyrimidine-base isomer) − Δ*G*(model-azine isomer). Since the electron-accepting endo N atoms participate in tautomeric conversions effects of the additional electron-donating exo OH or NH_2_ group in pyrimidine bases are strongly perturbed by both their intramolecular interactions with neighboring atoms or groups and electronic-effect changes of endo =N/>NH (Table 4). These additional internal changes dramatically affect the δ*G*s for the N1H and N3H forms. They vary from the negative to positive values (from −3 to 6 kcal mol^−1^). These variations clearly indicate that the δ*G*s correspond to the sum of all possible internal effects of the additional exo group. 

### 6.4. N-Endo Effects on Tautomeric Preferences

When the endo N atom in aromatic model-azines participates in tautomeric conversions and attaches the labile proton, it completely changes the type of tautomeric conversions from keto-enol in phenol to amide-iminol in mono-hydroxy derivatives and from imine-enamine in aniline to imine-amine (amidine) in mono-amino derivatives (see Figure 1, Figure 2 and Figure 4). These variations in tautomeric equilibria significantly influence the stability of the isomers **a**–**d** in model azines and the composition of tautomeric mixtures. The Δ*G* values calculated at the DFT level for monosubstituted azines strongly decrease for NH tautomers with the endo NH group when compared to the analogous CH isomers in the parent compounds. For mono-hydroxy azines, the Δ*G*s are reduced by 16–19 and 9–15 kcal mol^−^^1^ for isomers with the endo NH group at the 2- and 4-position vis-à-vis the exo oxo group, respectively [18,49]. For mono-amino derivatives, these effects are slightly weaker. When going from unsubstituted aniline to its azine derivatives, Δ*G*s decrease at the same level of theory for analogous isomers by 11–15 and 4–10 kcal mol^−^^1^ [22,58,59,60]. This means that the NH tautomers with the endo NH group in azines are considerably more stable than the corresponding CH isomers in the parent systems. The differences result mainly from the different acid-base properties of the tautomeric sites: endo N(sp^2^)/N(sp^3^)H in the model azines and C(sp^2^)H/C(sp^3^)H_2_ in the parent compounds. 

Table 5 summarizes the percentage contents estimated for all possible tautomers–rotamers of the selected monosubstituted pyridines and pyrimidines in vacuo [49,58,59,60]. For hydroxy tautomers possessing different conformational isomers of the exo –OH group and for imino tautomers having different configurational isomers of the exo =NH group, two values are given in this table for the two extreme rotamers (**1** and **2**). A higher value refers to the rotamer with more favorable intramolecular interactions. Data for the parent systems [18,22], phenol and aniline, are also included in this table. The amounts for all isomeric structures have been calculated on the basis of the relative Gibbs energies (Δ*G*_i_ at 298.15 K) calculated for individual isomers at the same level of theory {B3LYP/6-311+G(d,p)}. Owing to some discrepancies between the experimental and DFT-computed results for mono-hydroxy azines, the percentage contents for isomers of **2HOPY**, **4HOPY**, **2HOPM**, and **4HOPM** have been calculated at the G2 level [49], and listed in Table S7 (Supplementary Materials). 

The isomeric mixtures of mono-amino derivatives (**2APY**, **4APY**, **2APM**, and **4APM**) consist mainly of the aromatic amino forms (**a**, 100%). The other tautomers can be neglected for neutral derivatives in their ground states (<1 ppm). An exception is a photo-induced tautomeric conversion. The reversible amino-imino prototropic equilibria have been observed experimentally between **2APY-a** and **2APY-b** in a low-temperature argon matrix IR spectra upon UV irradiation [79]. A different situation takes place for neutral mono-hydroxy derivatives (**2HOPY**, **4HOPY**, **2HOPM**, and **4HOPM**), for which at least two tautomers, OH (conformation **a1**) and NH, can be detected in their ground states (Table 5). The differences in composition of the isomeric mixtures of mono-hydroxy and mono-amino aromatic azines confirm different acid-base properties of the keto-enol, imine-enamine, amide-iminol, and imine-amine (amidine) tautomeric moieties resulting from the different electronegativity of C, N, and O atoms, as well as from internal effects, particularly intramolecular interactions and push–pull electronic effects between tautomeric groups, and also electron delocalization in the isomeric systems [2,4,13,27,49,58,59,60,61].

The DFT-calculated tautomeric preference for **2HOPY** (Table 5) is different than that found at the G2 level (Table S7 in Supplementary Materials) [48,49]. The G2 theory predicts the less aromatic NH isomer (**b**) as a minor form (27%) for **2HOPY**, while the DFT method indicates it as a major form (82%). The G2 theory reproduces well the experimental result, ca. 75 for **a1** (OH) and 25% for **b** (NH) for **2HOPY**. In the case of **4HOPM**, the two methods predict the **b** form as a favored tautomer, but the G2 method (80%) reproduces better experimental results (70%) than the DFT one (97%). The OH rotamer **a2** has not been experimentally detected, indicating that its amount is considerably lower than 0.1%, and, thus, it could not be distinguished from the background. For experiments, different methods have been applied such as equilibrium methods, supersonic jet UV, matrix-isolation IR, MW, X-ray-PES, and free jet millimeter-wave [2,4,5,80,81,82,83,84,85,86,87]. In the case of symmetrically substituted **4HOPY** and **2HOPM**, both the DFT and G2 methods predict tautomeric preferences analogous to those identified experimentally. Note that only the OH tautomers (100%) have been detected in the gas phase [80,86]. Signals for the NH tautomers have not been found.

Investigating mono-hydroxy aromatic azines, we noticed, analogously to some other scientists, that the theoretical prediction for a tautomer possessing the lowest Gibbs energy depends on the level of calculations, particularly in the case of **2HOPY**. For this derivative, the calculated relative Gibbs energies of the OH and NH tautomers are close to zero. They vary from negative (ca. −1 kcal mol^−1^) to positive values (ca. 1 kcal mol^−1^) at different levels of theory [48,49]. Consequently, the mole fractions estimated on the basis of Δ*G*s (see equation in Section 3) also change. When calculations predict the negative Δ*G* value, this suggests that the NH tautomer predominates in the tautomeric mixture, whereas when they give the positive Δ*G* value, this indicates that the OH tautomer is favored. The different results can be a consequence of some computational errors that can play an important role in the percentage-content predictions for tautomers of close stability, i.e., when tautomeric equilibrium constants are close to unity (Δ*G* ≈ 0 kcal mol^−1^). 

Comparing computational with experimental results, we can see that the ‘best’ computational predictions for **2HOPY**, close to the experimental ones, have been reported only at a few levels of theory, e.g., semi-empirical AM1 and ab initio G2 or G2(MP2) methods [48,49]. Unfortunately, the DFT(B3LYP)/6-311+G(d,p) level of theory predicts the negative Δ*G* value (−0.9 kcal mol^−1^) for **2HOPY** and the reverse relation between the OH and NH forms [48,49,72,88], while the G2 theory gives the positive Δ*G* value (0.5 kcal mol^−1^) [48,49], being in accordance with the experimental data [2,4,5,80,81,82,83,84,85,86,87]. The difference between these two Δ*G* values is not high (1.4 kcal mol^−1^), but significant in the favored-tautomer prediction. Nevertheless, independently of the level of calculations, the CH tautomers are exceptionally rare forms for neutral derivatives, and only in particular cases can they be considered in the isomeric mixtures of azines. Computational difference between the DFT and G2 results (<2 kcal mol^−1^) have only a slight influence on the exceptionally high relative Gibbs energies of the CH tautomers (>20 kcal mol^−1^) [48,49].

**Figure 10:**
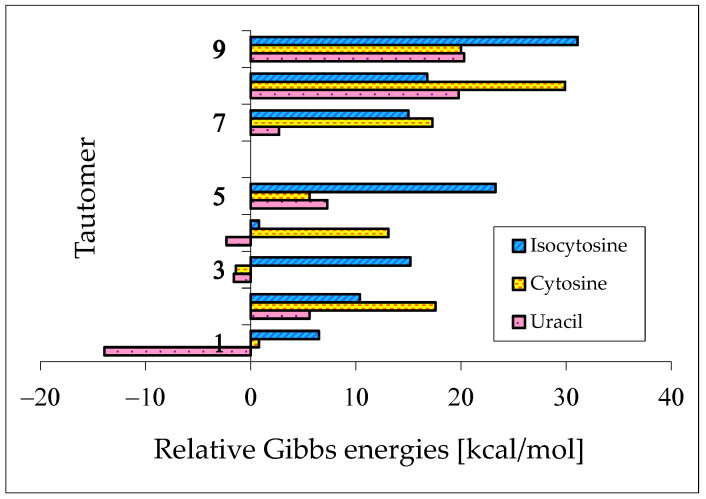
DFT-calculated relative Gibbs energies for selected tautomers–rotamers of pyrimidine bases. The numbers **1**–**9** correspond to the favored isomers of **13**, **17**, **18**, **37**, **38**, **78**, **35**, **57**, and **58**. For **6** (favored isomer of **78**), Δ*G* = 0 kcal mol^−1^. Data taken from refs. [62,64,65].

The presence of two exo groups (OH and/or NH_2_) and two endo N atoms in pyrimidine bases completely changes the internal effects and acid–base properties of tautomeric sites that their ability to attach the labile protons is different from that in model compounds [62,64,65,78]. The largest variations in the isomeric-mixture composition occur for uracil containing two exo OH groups. The aromatic isomer **U78aa**, stabilized by intramolecular interactions between the exo and endo groups, becomes a rare form. When two labile protons move from the exo to endo heteroatoms, the Gibbs energy considerably decreases {by ca. 14 kcal mol^−^^1^ at the B3LYP/6-311+G(d,p) level}, and the tautomeric conversions lead to the favored form **U13** [62]. For analogous conversions in cytosine and isocytosine (containing only one OH group and one NH_2_ group), reverse energetic effects take place. The Gibbs energies increase in a higher degree when going from **iC78a** to **iC13b** than from **C78a** to **C13b** (by 6.5 and 0.8 kcal mol^−^^1^, respectively, at the same DFT level) [64,65]. The variations of the relative Gibbs energies computed at the same level of theory for nine individual tautomers of pyrimidine bases are shown in Figure 10. For clarity of the picture, the Δ*G* values of the favored conformational and/or configurational isomers are only taken into account for each tautomer. The numbers **1**–**9** in this figure correspond to the pyrimidine-base tautomers **13**, **17**, **18**, **37**, **38**, **78**, **35**, **57**, and **58**. All Gibbs energies refer to the aromatic tautomer **78** (number **6**)—its isomer **aa** stabilized by intramolecular interactions between the exo and endo groups, selected as a reference tautomer (Δ*G* = 0 kcal mol^−^^1^). Scatter plots between Δ*G*s for all isomers of pyrimidine bases are shown in Figure S2 (Supplementary Materials).

When compared to the parent systems {phenol and aniline that prefer the aromatic hydroxy and amino forms, respectively (tautomer **a** in Figure 1)}, the strongest effects of functional groups on tautomeric preferences take place for uracil. The stability of two amide functions in **U** strongly favors the tautomer **U13** (100%)—number **1** in Figure 10. Note that the Gibbs energy of **U35** (CH tautomer—number 7 in Figure 10) is higher than that of the aromatic form **U78aa** by only 2.7 kcal mol^−^^1^. Hence, we do not find the reasons for which this isomer has not been investigated earlier by quantum-chemical methods. An analogous difference can be found in the future for thymine between **T78aa** and **T35**. 

Using various spectroscopic techniques (UV, Raman, IR, NMR, MW, etc.), the tautomer **13** has been found to be the favored form (100%) for **U** and **T** in the gas phase, solution, and solid state [2,4,5,89,90,91,92]. Very exciting experiments have been reported by Ito and co-workers [93,94], who also detected another rare isomer of **U** and **T** using the fluorescence excitation and dispersed fluorescence spectra of jet-cooled molecules. Considering the rare tautomers as impurities with particular fluorescence properties (exceptionally high fluorescence yields), they also observed the hydroxy-oxo isomers in the gas phase at about 200 °C. According to the tautomer-stability order predicted by quantum-chemical methods, they attributed the detected rare tautomers to **U37** and **T37**. 

The effects of functional groups on tautomeric preferences in cytosine and isocytosine containing only one amide-iminol tautomeric moiety seem to be considerably weaker than those in uracil. Computations at the same level of theory {B3LYP/6-311+G(d,p)} predict the minor amounts of **C13** and **iC13**. Other tautomers are most important in the tautomeric mixtures of these two bases. If we consider the absolute Δ*G* values for significant tautomers not higher than 10 kcal mol^−1^, their tautomeric mixtures consist of six isomers in the following orders: **C18** (87.0%), **C78a** (8.2%), **C78b** (2.5%), **C13b** (2.1%), **C13a** (0.1%), and **C38** (<0.1%) for cytosine [64], and **iC78a** (79.6%), **iC37** (20.4%), **iC78b** (<0.1%), **iC13b** (<0.1%), **iC13a** (<0.1%), and **iC17** (<0.1%) for isocytosine [65]. All of them, except for **C13** and **iC13**, contain the exo –NH_2_ group that is more favored than the exo =NH one. The differences between the Gibbs energies of CH tautomers (**C35a** and **iC35a**) and the most aromatic forms (**C78a** and **iC78a**) are considerably higher (17.7 and 15.0 kcal mol^−1^, respectively) than that for uracil.

Numerous experiments carried out for cytosine have been reviewed in refs. [2,4,5,40,41,42,64]. Chemists noticed that the tautomeric preferences for cytosine strongly depend on the environment. The canonical form **C18** has been detected in the solid state, whereas a mixture of at least two isomers **C18** and **C38** have been observed in an aqueous solution [2,4,92,95,96,97]. Depending on the experimental method applied to gaseous cytosine {matrix isolation IR and UV, MW, REMPI (resonantly enhanced multiphoton ionization), IR laser in helium nanodroplets, MS (mass spectrometry), core-level X-ray photoemission, and near-edge X-ray absorption}, two (**C18** and **C78**) or three (**C18**, **C78**, and **C13**) tautomers have been identified [2,4,5,92,98,99,100,101,102]. Recently, Alonso et al. [103], who applied a laser-ablation molecular-beam Fourier-transform microwave (LA-MB-FT-MW) spectroscopy and quantum-chemical calculations, detected five tautomers-rotamers (**C18**, **C78a**, **C78b**, **C13a**, and **C13b**)—almost all isomers predicted by quantum-chemical calculations with Δ*G* < 10 kcal mol^−^^1^.

In the case of isocytosine, different experimental tautomeric preferences when going from the solid state and aqueous solution to the vapor phase have also been reported [104,105,106,107,108]. Interestingly, two oxo isomers (**iC17** and **iC37**, ratio 1:1) have been detected in the solid state [104]. These isomers form the associated crystal structure with similar intermolecular H-bonds to those for the **C-G** pair in nucleic acids. The tautomers **iC17** and **iC37** also predominate in an aqueous solution as proven by NMR spectra [105]. Proceeding from a polar environment to the gas phase, the tautomeric preferences change. For isocytosine isolated in argon and neon matrices, the aromatic form **iC78** has been detected in IR spectra, and its transformation into the minor isomer **iC37** has been induced by UV irradiation [106]. This isomerization has been also studied by quantum-chemical calculations [106,107,108]. 

## 7. Alternation of Bond Lengths in Tautomers–Rotamers of Pyrimidine Bases and Their Models

**Figure 11:**
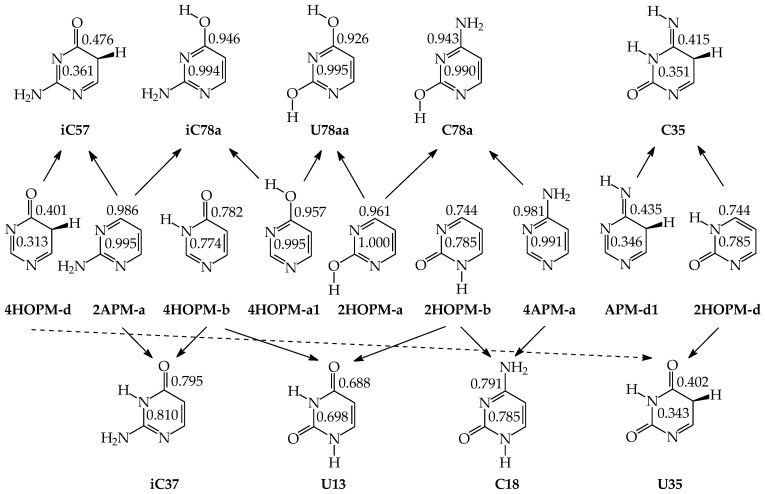
Variations of HOMEDs estimated for selected isomers of model azines and pyrimidine bases. HOMED for the six-membered ring included in the ring and that for the entire molecule placed near formula. DFT-calculated HOMED data taken from refs. [28,49,57,58,59,60,61,62,63,64,65].

The alternation of bond lengths in all possible isomers of the investigated compounds has been quantitatively determined by means of the geometry-based HOMED index [67,68]. This structural descriptor has been applied to the six-membered rings (*n* = 6) present in all tautomers-rotamers of phenol, aniline, azines, and pyrimidine bases, and, additionally, to the entire molecules (including exo groups) of mono- (*n* = 7), and disubstituted derivatives (*n* = 8) [18,22,28,49,57,58,59,60,61,62,63,64,65]. Depending on the number of bonds considered in the investigated tautomeric systems, the following abbreviations are used: HOMED6 (six bonds), HOMED7 (seven bonds), and HOMED8 (eight bonds). For tautomers-rotamers containing the labile proton(s) at the exo group(s), HOMED6s, HOMED7s, and HOMED8s are close to unity. These values confirm full electron delocalization (aromaticity) in the six-membered rings, as well as in the entire molecules. Medium electron delocalization (HOMEDs between 0.6 and 0.9) takes place for isomers possessing the labile proton(s) at the endo N atom(s). During the tautomeric proton-transfer(s) from the exo group(s) to the endo N atom(s), one or two pairs of π-electrons in the ring are replaced by one or two pairs of n-electrons on the endo N atom(s), and, finally, in these isomers, n-electrons are conjugated with the remaining π-electrons in the six-membered ring. Weak electron delocalization (HOMEDs lower than 0.6) occurs for isomers with the labile proton at the endo C atom. The presence of the endo C-sp^3^ atom destructs full electron delocalization in the six-membered ring, and, consequently, strongly reduces electron delocalization in the ring and entire molecule. Figure 11 illustrates the changes of HOMEDs when going from isomers of model azines to canonical pyrimidine bases (**U13**, **C18**, and **iC37**), their aromatic forms (**U78aa**, **C78a**, and **iC78a**, stabilized by favorable intramolecular interactions between functional groups), and also CH tautomers (**U35**, **C35**, and **iC57**). 

The comparison of data in Figure 11 shows that greater HOMED variations occur for **U13** containing two endo NH groups than for **C18** and **iC37** with only one endo NH. In the other words, a greater number of n-electrons in the six-membered ring means weaker ring electron-delocalization. On the other hand, HOMED6s and HOMED8s for the aromatic forms (**U78aa**, **C78a**, and **iC78a**) do not vary very much. They describe the same types of electron delocalization in the six-membered ring (aromaticity, HOMED6s ≥ 0.99), and cross conjugation of n-electrons on exo heteroatoms with six π-electrons of the ring in the entire molecule (HOMED8s between 0.92 and 0.95). For model aromatic azines possessing one exo heterogroup (OH or NH_2_) or one endo NH group, HOMED6s are only slightly higher than those for disubstituted pyrimidine bases (ΔHOMED6 ≤ 0.01). Greater differences take place between HOMED7s and HOMED8s corresponding to the entire molecules (ΔHOMED 0.01–0.04), when n-electrons are mixed with π-electrons. More exo groups (more n-electrons) means slightly weaker electron delocalization in the system and a smaller HOMED.

**Figure 12:**
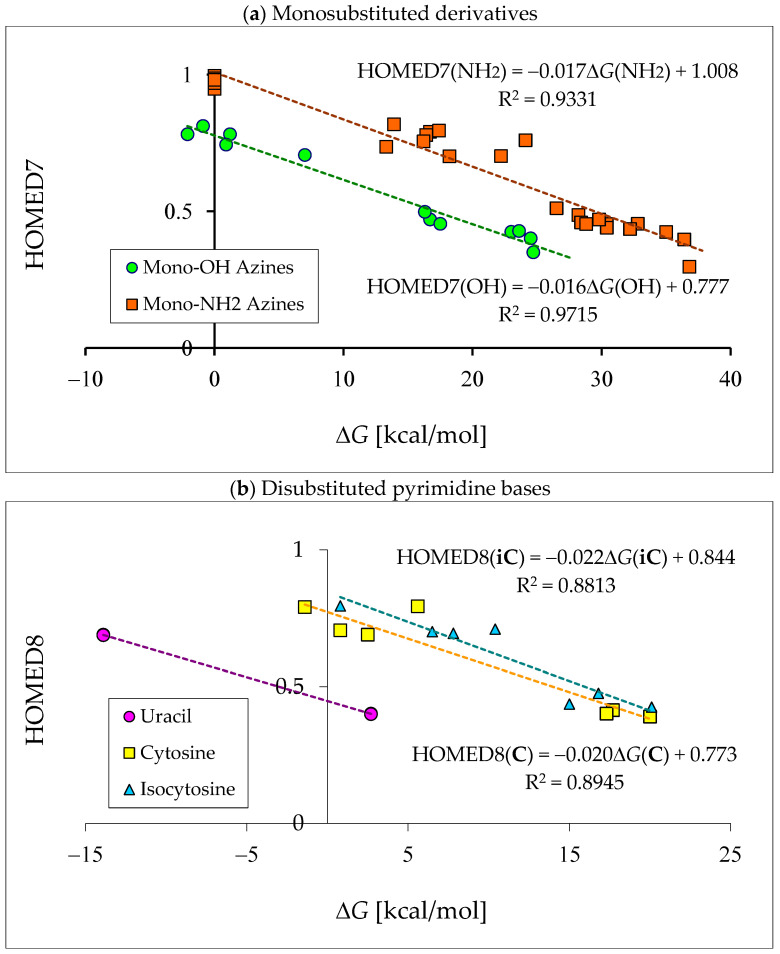
Linear trends for monosubstituted azines including parent systems (**a**) and disubstituted pyrimidine bases (**b**) between HOMEDs (for entire molecule) and Δ*G*s for isomers containing labile proton(s) at N and/or C atoms. DFT-calculated geometric and energetic data taken from refs. [18,22,28,49,57,58,59,60,61,62,63,64,65].

During investigations of the complete isomeric mixture for adenine and their model compounds (imidazole, 4-aminopyrimidine, and purine), we found that changes of HOMEDs are parallel to those of Δ*G*s, both calculated at the same level of theory {DFT(B3LYP)/6-311+G(d,p)} for tautomers–rotamers containing the labile proton(s) at the N and/or C atoms [35]. Analogous linear trends (Figure 12) can be distinguished for monosubstituted aromatic azines and their parent compounds, as well as for disubstituted pyrimidine bases (**U**, **C**, and **iC**). Note that there are only two tautomers for **U** (**13** and **35**) containing the labile protons only at the N and/or C atoms, and, thus, only two points could be included in Figure 12. More points are possible for cytosine and isocytosine. They correspond to tautomers–rotamers without exo OH (**13**, **18**, **35**, **38**, and **58** for **C**, and **13**, **17**, **35**, **37**, and **57** for **iC**). For monosubstituted derivatives, points referring to the tautomers **b**–**d** of mono-hydroxy derivatives and to the isomers **a**–**d** of mono-amino derivatives are also included in Figure 12. 

When we plot, additionally, the points corresponding to isomers possessing the tautomeric proton at the exo OH group, i.e., OH isomer (**a**) for phenol and OH isomers (**a1** and **a2**) for mono-hydroxy azines, the added points deviate from the linear trend observed for the NH and CH tautomers of monosubstituted derivatives [49]. The same is true for disubstituted pyrimidine bases. When we plot, additionally, the points referring to isomers containing the labile protons at one or two exo OH groups, the added points for OHOH (**78**), NHOH (**17**, **18**, **37**, and **38**) and CHOH isomers (**57** and **58**) for uracil, NHOH (**17**, **37**, and **78**) and CHOH isomers (**57**) for cytosine, and also NHOH (**18**, **38**, and **78**) and CHOH isomers (**58**) for isocytosine strongly deviate from the linear trends observed for the NHNH and CHNH tautomers of **U**, **C**, and **iC** [65]. Figures S3 and S4 (Supplementary Materials) show scatter plots rather than linear trends between the HOMEDs and Δ*G*s for all possible tautomers–rotamers of pyrimidine bases and mono-hydroxy model derivatives. 

**Figure 13:**
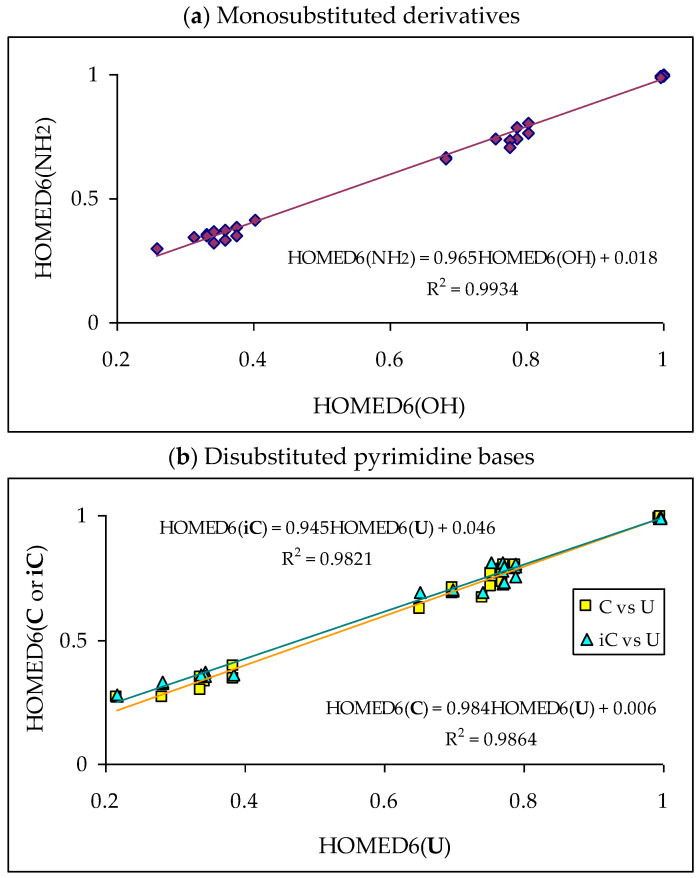
Linear relations between HOMED6s estimated for tautomers–rotamers of mono-amino and mono-hydroxy aromatic derivatives (**a**) and for isomers of disubstituted pyrimidine bases (**b**). DFT-calculated HOMED6s for the six-membered ring taken from refs. [18,22,28,49,57,58,59,60,61,62,63,64,65].

However, no particular deviations exist for geometric parameters (HOMEDs) when a series of analogous derivatives are compared. Isomeric conversions in mono-hydroxy and mono-amino derivatives dictate similar changes in electron delocalization. Individual isomers display analogous types of mixed conjugations (π-π, n-π, and/or σ-π). The same trends occur for disubstituted pyrimidine bases. Hence, we observe parallel variations in bond length alternations, and, consequently, in HOMEDs (Figure 13). 

When we plot the HOMED6 indices calculated for all possible isomers of mono-amino derivatives against those of mono-hydroxy derivatives, a good linear relation exists between them [49]. Linear relations also occur for all possible tautomers–rotamers of pyrimidines bases (**U**, **C**, and **iC**). The HOMED6 indices estimated for isomers of **C** and **iC** are parallel to those of **U** [65]. Additionally, linear trends exist between HOMEDs calculated for the entire molecules of mono- (HOMED7) and disubstituted (HOMED8) derivatives [49,65]. Figure 13 presents good correlations (with exceptionally high correlation coefficients, R ≥ 0.99) between the HOMED6 indices for the analogous series of mono- and disubstituted derivatives. This means that the lack of parallelism between HOMEDs and Δ*G*s for all possible isomers of mono- and disubstituted title compounds can only be explained by significant differences in energetic parameters between isomers with and without the exo OH group(s).

It should be recalled here that the HOMA procedure reformulated by Krygowski in 1993 [71] cannot be applied to the complete tautomeric mixtures of tautomeric heterocompounds. Figure S5 (Supplementary Materials) shows typical discrepancies between the HOMED and reformulated HOMA values, estimated for the six-membered ring (*n* = 6) and for the entire molecule (*n* = 8) of all possible pyrimidine-base isomers. The reformulated HOMA indices are strongly negative for CH isomers (**35**, **57**, and **58**), and they are close to unity for isomers with the labile proton(s) at the exo groups (**78**). The reformulated HOMA scale is artificially extended. Consequently, there are no linear relations between the structural descriptors. For more details on the lack of linear trends between the reformulated HOMA and HOMED indices for other heterocompounds, see refs. [35,65,67,68]. The examples discussed in these articles clearly show that the reformulated HOMA index cannot be applied to differently delocalized OH, NH, and CH isomers of monosubstituted derivatives, nor to differently delocalized OHOH, NHOH, NHNH, CHOH, and CHNH isomers of disubstituted pyrimidine bases. It can only be employed for compounds containing the same type of bonds, e.g., hydrocarbons, or for well-delocalized (aromatic) heterosystems with HOMA close to unity.

## 8. Ionization Effects on Prototropy and Electron Delocalization in Mono- and Disubstituted Derivatives

Positive or negative ionization, also called one-electron loss (**M** − e → **M**^+●^, where **M** is a molecule) or one-electron gain (**M** + e → **M**^−●^), respectively, changes the electronic states of tautomeric compounds from neutral forms to charged radicals. Ionization also affects the acid–base properties of functional groups. In consequence, ionization influences the composition of isomeric mixtures and tautomeric preferences [18,22,28,49,57,58,59,60,61,62,63,64,65]. However, chemists investigating ionization energies (IE) or electron affinities (EA) for nucleobases by means of quantum-chemical methods, considered most frequently the favored (canonical) tautomers identified for neutral forms. The calculated energetic parameters for ionization processes have been attributed to so-called vertical or adiabatic IE, and vertical or adiabatic EA. For unknown reasons, most chemists have not considered prototropic conversions in charged radicals. In the literature, we can find only a few reports in which some tautomeric charged-radicals have been analyzed. They are cited below for particular derivatives. 

Very exciting studies have been documented by Choi et al. [109], who experimentally demonstrated that intramolecular proton-transfer in one isomer and the formation of another one is possible and can be detected in the guanine radical-cation (G^+●^). For investigations, time-resolved resonance Raman spectroscopy combined with pulse radiolysis has been employed. Different tautomers have been also identified by Tureček and co-workers [110] for adenine radical-cation (A^+●^). In this case, chemists applied the collision-induced dissociation mass spectrometry (CID MS) technique and UV-vis photodissociation (UVPD) action spectroscopy. On the other hand, Bowen, Gutowski, Rak, and their co-workers [76], who studied nucleic acid bases by negative ion PES experiments, suggested that intramolecular proton-transfer takes place for the canonical tautomers of nucleic acid bases. This suggestion has been formulated to explain the formation of rare isomers strongly stabilized by one excess electron. The different experiments carried out for positively and negatively ionized bases revealed that they can exhibit prototropy in vacuo, and also indicated that the complete tautomeric mixtures have to be studied by quantum-chemical methods to select the favored isomers. In this chapter, we review the consequences of positive and negative ionizations in derivatives containing the six-membered ring, starting from the parent systems (phenol and aniline) and going through model compounds (mono-hydroxy and mono-amino aromatic azines) to pyrimidine nucleic acid bases (**U**, **C**, and **iC**).

### 8.1. Parent Compounds

**Figure 14:**
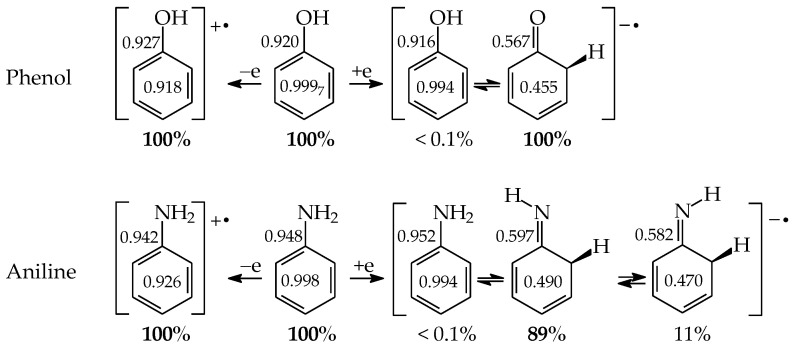
Consequences of positive and negative ionization on composition of isomeric mixtures and electron delocalization for phenol and aniline. DFT-estimated percentage contents given below formula, HOMED6s included in the ring, and HOMED7s placed near the ring. Data taken from refs. [18,22].

When proceeding from the neutral to ionized species of the parent compounds, the DFT-calculated Δ*G* values for the structures **a**–**d** (Figure 1) vary in a higher degree for radical anions (PhOH^−●^ and PhNH_2_^−●^) than for radical cations (PhOH^+●^ and PhNH_2_^+●^) [18,22]. The aromatic hydroxy and amino isomers **a** containing the labile proton at the exo heteroatoms predominate for PhOH^+●^ and PhNH_2_^+●^, respectively, like for PhOH and PhNH_2_ (Figure 14). The other isomers (CH forms **b**–**d**) are unlikely forms for positively charged species as for neutral derivatives. Nguyen and co-workers [111,112,113], generating the isomer **a** of PhOH^+●^ and PhNH_2_^+●^ by means of simple mass spectrometry (MS) techniques, also detected less stable keto and imino isomers (**b**–**d**), but only in tandem mass spectrometry (MS/MS/MS) experiments. 

A significant change of the tautomeric preferences takes place for radical anions of the parent systems (PhOH^−●^ and PhNH_2_^−●^), for which some CH isomers have considerably lower Gibbs energies than the aromatic hydroxy and amino forms **a** [18,22]. The labile proton prefers the position 2- or 6- in the ring, and the corresponding CH isomers are preferred for reduced compounds. The tautomeric conversion can take place in reduced phenol and aniline according to the 1,3- or 1,7-proton shift.

As could be expected, one-electron loss and one-electron gain also affect the bond length alternation, and, consequently, the structural descriptors in the parent compounds [18,22]. HOMED6 and HOMED7, estimated for the DFT-structures, change slightly (generally decrease) for the aromatic hydroxy and amino forms **a**; however, their values are not lower than 0.9 (Figure 14). This means that, after ionization, each of these isomers continues exhibiting an aromatic character. The effects of ionization on electron delocalization in CH isomers (**b**–**d**) are considerably stronger than those in aromatic ones. Generally, π-electrons seem to be more delocalized in the charged radicals than in the neutral forms. HOMED6s and HOMED7s increase in a higher degree for radical cations (by 0.2–0.3 HOMED units) than for radical anions (by ca. 0.1 HOMED units) [18,22].

### 8.2. Monosubstituted Azines

**Figure 15:**
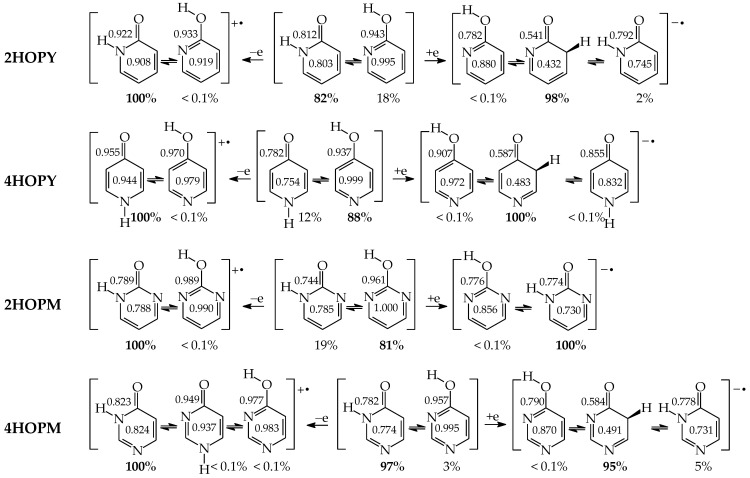

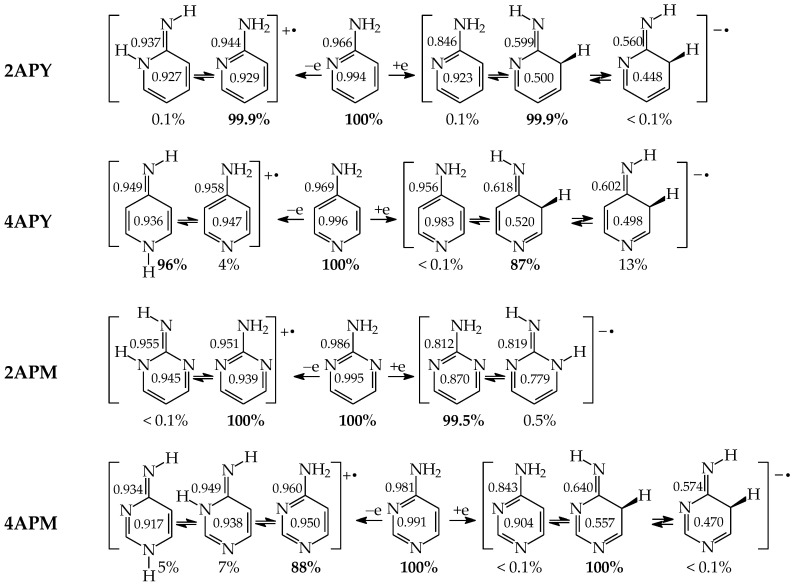
Consequences of positive and negative ionization on isomeric-mixture composition and electron delocalization for mono-hydroxy (**2HOPY**, **4HOPY**, **2HOPM**, and **4HOPM**) and mono-amino azines (**2APY**, **4APY**, **2APM**, and **4APM**). DFT-estimated percentage contents given below formula, HOMED6s included in the ring, and HOMED7s placed near the ring. Data taken from refs. [49,58,59,60,61].

For mono-hydroxy aromatic azines, one-electron loss strongly favors isomers containing the labile proton at the endo N atom [49]. The oxidized derivatives consist mainly of the NH tautomers (Figure 15), contrary to neutral hydroxy azines for which two tautomers (OH and NH) are favored in the tautomeric mixtures (Table 5). The DFT results for oxidized derivatives are consistent with the experiments of Ozeki et al. [114], who showed, by a two-color resonantly enhanced multiphoton ionization zero kinetic energy (REMPI ZEKE) positive ion PES experiment, that the gaseous radical cation of 2-pyridone (**b**) is more stable than that of 2-hydroxypyridine (**a1**). 

A slightly different situation occurs for positively ionized mono-amino aromatic azines [58,59,60,61]. In most of cases, the isomer **a** (amino tautomer) is favored. The Δ*G*s of the imino NH tautomers-rotamers significantly decrease for radical cations. However, only in the case of oxidized 4-aminopyridine, the imino NH isomer (**c**) seems to be the favored form in the isomeric mixture. For other positively ionized amino derivatives, the imino NH isomers are minor or rare forms. Analogous to oxidized parent systems, the CH isomers are unlikely forms for all oxidized model azines. 

The negative ionization of mono-hydroxy and mono-amino aromatic azines dramatically changes the composition of tautomeric mixtures [49,58,59,60,61]. The CH isomers containing the labile proton at 2- or 6-position vis-à-vis the exo group are favored forms in the gas phase for negatively ionized azines (Figure 15). Some exceptions are observed for negatively ionized 2-hydroxy and 2-aminopyrimidines, for which two endo N atoms are placed at the 2- and 6-positions vis-à-vis the exo group. Hence, the NH isomer predominates for one derivative, and the amino tautomer (with a slight amount of the imino NH form) is favored for the other one, respectively. 

The HOMED6 and HOMED7 indices also change when proceeding from neutral to ionized isomers [49,58,59,60,61]. Nevertheless, the HOMED variations are slightly different than those for the parent systems, particularly for tautomers containing the labile proton at the endo N atom(s). In many cases, their values for the ionized forms are larger than those for the neutral ones. It should be mentioned here that non-parallel variations exist between the energetic (Δ*G*s) and separately between the geometric (HOMED) parameters of the neutral and ionized isomers. This lack of linear trends confirms that the mechanisms of one-electron loss and one-electron gain are not the same for the series of model azine-isomers. 

### 8.3. Disubstituted Pyrimidine Bases

**Figure 16:**
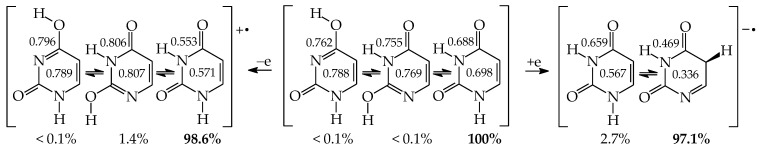
Variations in tautomeric-mixture composition and electron delocalization for neutral and ionized uracil. DFT-estimated percentage contents given below formula, HOMED6s included in the ring, and HOMED8s placed near the ring, all taken from refs. [62,63].

Different consequences of one-electron loss or one-electron gain, already observed for the parent and model compounds, also take place for pyrimidine bases when proceeding from the complete neutral tautomeric mixtures to their ionized forms [28,57,62,63,64,65]. As mentioned above, neutral uracil strongly favors the dioxo form **13** [2,3,4,5,62,89,90,91,92,93,94]. In particular experimental conditions, the rare hydroxy-oxo forms **18a** and **37a** (Δ*G* ca. 12 kcal mol^−^^1^, respectively) can also be identified [93,94]. Our DFT-calculated Gibbs energies for these two neutral isomers differ by only 0.7 kcal mol^−^^1^ [62]. Positive ionization does not change the stability order of these isomers, but reduces their DFT-calculated Δ*G*s to 6.7 and 2.5 kcal mol^−^^1^, respectively, and increases their amounts in the isomeric mixture of **U^+^^●^** [63]. The oxidized oxo-CH tautomers (**35**, **57**, and **58**) are exceptionally rare forms for **U^+^^●^**, like for **U**. Analogous conclusions have been derived by Tureček and co-workers [115], who considered theoretically {B3LYP and QCISD(T)} only ten isomers for **U^+^^●^** (**13**, **17b**, **18a**, **35**, **37a**, **38a**, **38b**, **57a**, **58a**, and **78aa**), and also by Yáñez and co-workers [116], who studied theoretically (B3LYP and G4) only four isomers (**13**, **17b**, **18a**, and **37a**) for **U^+^^●^**. The canonical radical cation seems to be the main form in the isomeric mixture of **U^+^^●^** (Figure 16). The other isomers can be considered as minor, rare, or exceptionally rare forms. 

Negative ionization completely changes the relative stabilities of **U^−^^●^** isomers. DFT-calculations predict the oxo-CH form **35** as a predominant tautomer for **U^−^^●^** [63]. Analogous to mono-hydroxy derivatives, one-electron gain favors in uracil the endo C atom at the 5-position for the labile-proton attachment. The canonical dioxo tautomer **13**—the exceptionally stable form for **U** and **U^+^^●^**—loses its stability during negative ionization and becomes a minor isomer for **U^−^^●^**. The other oxo-CH (**57** and **58**), hydroxy-oxo (**17**, **18**, **37** and **38**), and dihydroxy (**78**) tautomers are rare or exceptionally rare forms for **U^−^^●^**. Analogous conclusions have been formulated by Bachorz et al. [117] on the basis of the negative ion PES experiment and various quantum-chemical calculations {B3LYP, MP2, and CCSD(T)} carried out for uracil stabilized by one excess electron. The CH tautomer **35** has been found as a favored form for the valence anion of uracil. Although Tureček, Yáñez, and their co-workers [115,116] also investigated **U^−^^●^**, the oxo-CH isomers have not been considered in their theoretical analyses. They performed quantum-chemical calculations only for two (**13** and **37a**) and four (**13**, **17b**, **18a**, and **37a**) isomers, respectively, and reported only partial results for **U^−^^●^**. 

Figure 16 illustrates changes in the tautomeric-mixture composition of **U^+^^●^**, **U^−^^●^** and **U**, measured by means of percentage contents calculated at the DFT level [62,63]. It also shows variations in electron delocalization when going from **U** to **U^+^^●^** and **U^−^^●^** isomers, measured by means of the HOMED6s and HOMED8s found for DFT-structures. The HOMED-variations indicate that electron delocalization is very sensitive to positive and negative ionization. Since n- and π-electrons are delocalized in neutral and ionized species, it is difficult to propose the mechanisms of positive and negative ionizations even for the major isomers without an additional detailed investigation by quantum-chemical calculations. Owing to different thermochemical ionization-parameters [63], we can only conclude that the mechanisms are different for individual isomers. Both Δ*G*s and HOMEDs do not correlate well for neutral and ionized isomers (Figures S6 and S7 in Supplementary Materials). 

In the case of **T^+^^●^**, very interesting results have been recently reported by Nguyen, Ryzhov, Tureček, and their co-workers [118], who employed different MS techniques, UV-vis photodissociation (UVPD) action spectroscopy, ion-molecule reactions, and quantum-chemical calculations {DFT with different functionals and CCSD(T)}. Experiments for **T^+^^●^** revealed that some isomers containing the exo CH_2_ group (instead of CH_3_) are more stable than the canonical isomer **13**, indicating the engagement of CH_3_ in intramolecular proton (or H atom) transfers in positively ionized thymine. Considering three labile protons in **T^+^^●^**, four stable isomers with the exo CH_2_ group have been proposed: **178**, **138**, **137**, and **378**, the mixture of which seems to be favored in **T^+^^●^** (77%). The canonical isomer **13** is a minor form (23%) for **T^+^^●^**. These preliminary experimental and theoretical investigations shed new light on isomerism in thymine and need further theoretical investigations for **T^+^^●^**, for which three labile H^+^/H**^●^** can move in isomeric conversions. Bowen, Gutowski, Rak, and their co-workers [76,119] also derived interesting conclusions for thymine exposed to low-energy electrons. Applying negative ion PES and MS spectra combined with quantum chemical calculations {B3LYP, MP2, CCSD(T)}, they indicated that the oxo-CH tautomer **35** can be considered as the favored isomer for **T^−^^●^**. Note that the engagement of the exo CH_3_ group in intramolecular H^+^/H**^●^**-transfers has not been considered in this investigation. Taking these results into account, prototropy in **T^−^^●^** also needs further studies.

Although cytosine and isocytosine contain only one exo OH and one NH_2_ group, they display a similar variety of the relative Gibbs energies for neutral CH isomers, like uracil (Δ*G*s ca. 40 kcal mol^−1^) [62,63,64,65]. However, for some NHOH and NHNH tautomers of cytosine, the Δ*G*s are lower than 5 kcal mol^−^^1^, whereas those for OHOH and NHOH isomers of uracil are considerably larger than 5 kcal mol^−^^1^. Consequently, the stability orders are not the same [28,57,62,63,64,65], and no linear trends exist between the Δ*G*s of neutral (Figure S2 in Supplementary Materials), as well as between the Δ*G*s of neutral and ionized tautomers–rotamers of pyrimidine bases (Figure S6 in Supplementary Materials). The same conclusion can be derived on the basis of the HOMED indices. There is no parallelism between HOMEDs of all neutral and ionized isomers of pyrimidine bases (Figure S7 in Supplementary Materials). 

**Figure 17:**
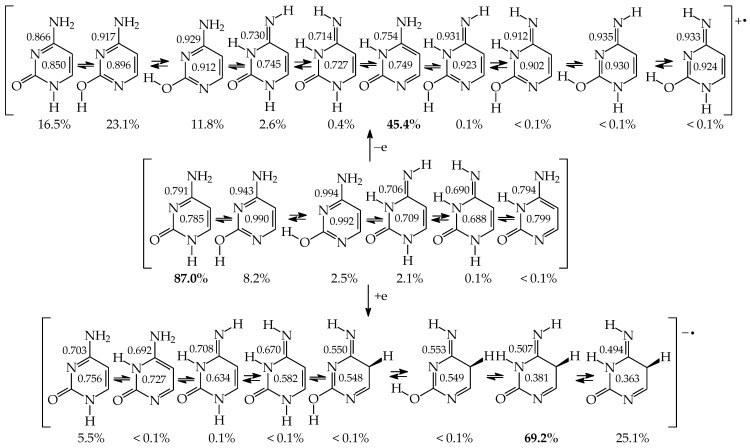
Variations of isomeric preferences and electron delocalization when proceeding from neutral to ionized cytosine. DFT-estimated percentage contents given below formulae, HOMED6s included in the ring, and HOMED8s placed near the ring, all taken from ref. [64].

According to our complete DFT calculations, the tautomeric mixtures of **C**, **C^+^^●^** and **C^−^^●^** consist of six, ten, and eight isomers, respectively, with Δ*G*s not larger than 10 kcal mol^−1^ [64]. Not all of them are the major or minor isomers. The canonical oxo-amino tautomer **18** is a major form for **C** (Figure 17). The two hydroxy-amino isomers **78a** and **78b**, and the two oxo-imino isomers **13a** and **13b** are minor forms. The oxo-amino tautomer **38** is a rare form. The other isomers as rare or exceptionally rare forms can be neglected in the isomeric mixture of **C** [64,103]. All CH isomers are exceptionally rare forms for **C** and **C^+^^●^**. However, positive ionization changes the isomeric-stability order for other isomers, and, consequently, changes the tautomeric preferences for **C^+^^●^**. The tautomer **38** seems to be more stable than **18**, although the two isomers are major forms for **C^+^^●^** together with **78a** and **78b** as found at the DFT level [64]. The isomeric mixture of **C^+^^●^** can also consist of three minor forms, **13a**, **13b**, and **37ab**, and three rare forms, **37aa**, **17ba** and **17bb**. 

Similar stabilities for **C^+^^●^** isomers have been reported by Tureček, Wesdemiotis, and their co-workers [120], who applied various quantum chemical methods {e.g., B3LYP and CCSD(T)} for seven isomers. They found that **38** has the smallest energy followed by **78a**, **78b**, **18**, **13b**, **37ab**, and **13a**. Since they calculated only the relative energies at 0 K, we can see some slight differences between this order and our order of the relative Gibbs energies determined at 298 K (**38**, **78a**, **18**, **78b**, **13b**, **13a**, **37ab**). Note that Δ*G*s contain, additionally, the relative thermal corrections and relative entropy terms. According to the literature on MS spectra, the isomeric mixture of **C^+^^●^** can equilibrate before fragmentation [120]. 

Tureček, Ryzhov, and their co-workers [121] analyzed, additionally, **C^+^^●^** isomers at room temperature combining various experimental techniques such as infrared multiple-photon dissociation (IRMPD) spectroscopy with a free electron laser in the fingerprint region, UV-vis photodissociation (UVPD) action spectroscopy, ion-molecule reactions, and also quantum-chemical calculations {DFT and CCSD(T)}. The experimental IRMPD and UVPD action spectra for **C^+^^●^** have been interpreted as a mixture of spectra calculated for isomers of the lowest energy. The IRMPD spectrum has been considered as a mixture of four **C^+^^●^** isomer, **38**, **18**, **78a**, and **78b**, while the UVPD spectrum is considered as a mixture of more numbers of **C^+^^●^** isomers. Note that only seven low-energy tautomers-rotamers have been considered for **C^+^^●^**. Interesting experiments for **C^+^^●^** have been recently reported by Grégoire and co-workers [122], who employed cryogenic IR and UV spectroscopy and also quantum-chemical calculations (TD-DFT and various CASSCF methods). They detected two oxo-amino tautomers (**17** and **37**) for **C^+^^●^** in the IR-UV cryogenic ion spectrum, recorded for a radical cation generated by the photodissociation of cold cytosine silver complex. More critical information on the experimental analysis of the nucleic acid bases radical cations can be found in the recent review article of Tureček [123].

Like for uracil, dramatic changes cause the negative ionization of cytosine. For **C^−^^●^**, the two oxo-imino CH isomers **35a** and **35b** are major forms, and the canonical tautomer **18** is a minor form, together with **13b** [64]. The isomer **13a**, **38**, **57aa**, and **57ba** are rare forms. The other isomers are very rare or exceptionally rare forms. As expected, positive and negative ionization also affect the HOMED indices. Their variations are shown in Figure 17. Experiments performed for negatively ionized cytosine by Bowen, Gutowski, Rak, and their co-workers [76] showed some resemblance of the PES spectra to those for uracil and thymine. Two bands have been detected, indicating that there are two different major isomers. This is in accordance with our DFT calculations. The favored form can refer to **35b** and the minor form to **35a**. However, authors attributed the two PES bands to the stable isomer **35b** and to less stable hypothetical form with two labile H**^●^**/H^+^ at N1 and C5. The CH isomer of cytosine (**35**) has also been identified as an intermediate form in the radical chemistry of the pyrimidine base [124]. 

**Figure 18:**
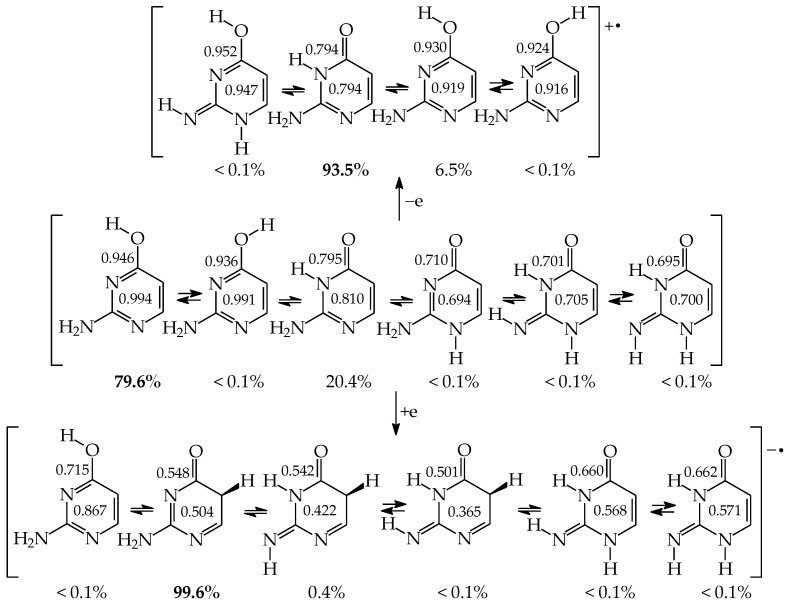
Variations of isomeric preferences and electron delocalization when proceeding from neutral to ionized isocytosine. DFT-estimated percentage contents given below formulae, HOMED6s included in the ring, and HOMED8s placed near the ring, all taken from refs. [28,57,65].

For the tautomeric mixtures of **iC**, **iC^+^^●^** and **iC^−^^●^**, four, six, and also six isomers (but not all the same), respectively, have been isolated by DFT calculations, and confirmed by the G4 method for selected low-energy isomers [28,57,65]. Exceptionally, one isomer of the most aromatic amino-hydroxy tautomer **78a** is favored for neutral **iC** in the gas phase (Figure 18). It is a major form together with the amino-oxo tautomer **37**, being the **iC** part in the canonical **G** isomer. The isomer **78b** is a rare form together with the amino-oxo tautomer **17**, and the two imino-oxo isomers **13a** and **13b**. 

Positive and negative ionizations change the isomeric-stability order and isomeric preference for isocytosine. The amino-oxo tautomer **37** is a favored form for **iC^+^^●^**, whereas the amino-oxo CH tautomer **57** predominates for **iC^−^^●^**. The amino-hydroxy isomer **78a** is a minor form and its rotamer **78b** together with the imino-hydroxy isomer **18ba** are rare forms for **iC^+^^●^**. The isomeric mixture of **iC^−^^●^** also contains the imino-oxo CH isomer **35a** as a minor form, and four isomers as rare forms: **35b**, **78a**, **13a**, and **13b**. From an energetic point of view (Δ*G* < 10 kcal mol^−1^), the other isomers can be neglected in the isomeric mixtures of **iC**, **iC^+^^●^** and **iC^−^^●^**. Figure 18 illustrates variations of isomeric preferences (percentage contents) and electron delocalization (HOMED indices) when going from neutral to positively and negatively ionized isocytosine. All parameters have been estimated at the same DFT level [28,57,65]. The literature experimental data for ionized isocytosine are exceptionally scarce. We found only one old experimental report on the ionic radical species of **iC^+^^●^** and **iC^−^^●^** detected in the 1970s by Herak et al. [125], who used electron spin resonance (ESR) spectroscopy for a single crystal of isocytosine, irradiated with γ-rays at 77 K. To our knowledge, there is no other document on ionized isocytosine in the gas phase. 

## 9. Conclusions

Although experimental techniques with their own limits give the possibility to identify the favored structures for isomeric organic compounds, including biomolecules, quantum-chemical methods have this advantage over experimental ones that all possible isomers can be investigated, even those experimentally inaccessible, and the complete information on isomeric systems can be derived. Experiments reveal mainly partial information, and no detailed and complete analyses on various effects that influence the isomer-stability and isomer-preference can be performed well. Our review evidently shows the pivotal role of the computational-method application to tautomeric systems, particularly to those in which prototropy governs their physicochemical, chemical, and biochemical properties. It also indicates how to explore tautomeric conversions in nucleic acid bases by means of quantum-chemical methods, starting from the theoretical aspects and definition of prototropy for simple derivatives (phenol and aniline) and proceeding to structural and energetic descriptors for all possible tautomers–rotamers of more complex nucleic acid bases (pyrimidine bases and their model compounds). In our extensive analyses of computational results, we could consider various factors such as different functional groups, their position within the ring, electronic effect, electron delocalization, and intramolecular interactions that influence tautomeric stabilities and tautomeric preferences.

Investigating the title compounds, first, we showed some structural similarity for the complete scheme of proton-transfers for each labile proton in mono- and disubstituted tautomeric derivatives containing the six-membered aromatic ring (Figure 1, Figure 2, Figure 3, Figure 4 and Figure 5). Although the number of tautomers (nine) for disubstituted pyrimidine bases (**U**, **T**, **C**, and **iC**) is different than that (four) for mono-hydroxy and mono-amino aromatic azines and their parent compounds (phenol and aniline), the labile protons move between conjugated atoms placed at the same positions vis-à-vis exo groups. In parallel, the labile electrons in all possible tautomers are delocalized in an analogous way. Consequently, good correlations exist between the geometry-based HOMED indices (structural measure of electron delocalization) estimated for isomers of neutral mono-hydroxy and mono-amino aromatic azines (including phenol and aniline), and, separately, for isomers of neutral disubstituted pyrimidine bases, **C**, **iC,** and **U** (Figure 13). 

Additionally, we found that the relative Gibbs energies (thermochemical measure of proton-transfer and tautomeric preference) correlate well with the HOMEDs for isomers of an analogous series of neutral derivatives (pyrimidine bases and model compounds) containing the labile protons at the conjugated N and/or C atoms (Figure 12). Some exceptions take place for hydroxy isomers which display particular stabilities in comparison to the other ones and deviate from the linear trends between Δ*G*s and HOMEDs. 

Next, we quantitatively confirmed that, from an energetic point of view, keto-enol (proton-transfers between conjugated O and C atoms) and imine-enamine equilibria (proton-transfers between conjugated N and C atoms) can be neglected for neutral tautomeric heterocompounds (pyrimidine bases and their models). All tautomers–rotamers containing the labile proton at the endo C atom are exceptionally rare forms. Their percentage contents are considerably lower than 0.0001 ppm. The tautomeric preferences for neutral derivatives in vacuo result mainly from the acid-base properties of tautomeric sites (N and O) and various internal effects. For neutral uracil and thymine, the stability of two amide functions seems to play a more important role than the aromaticity of the six-membered ring and intramolecular interactions between functional groups. This factor can only explain why the dioxo form **13** is strongly favored for **U** and **T**. In the case of **C** and **iC** containing only one tautomeric amide function, the difference between the aromatic and amide-group stabilities seems to be not as high as that in **U** and **T**. Aromatic isomers with favorable intramolecular interactions between the exo and endo groups contribute significantly to the isomeric mixtures of **C** and **iC**. The same is true for neutral monosubstituted aromatic azines.

Finally, we demonstrated that positive ionization does not change the tautomeric preferences for phenol and aniline in vacuo (Figure 14). However, when at least one endo N atom appears in the six-membered ring at the position conjugated with the exo group, it binds favorably the tautomeric proton in model azines (Figure 15). Some exceptions occur for **2APY^+^^●^**, **2APM^+^^●^**, and **2APM^+^^●^**, for which the aromatic amino-tautomers predominate. The canonical di-oxo tautomer (**13**) is favored only for **U^+^^●^** (Figure 16). This suggests the strong stability of the amide functions not only for neutral but also for oxidized uracil. For **C^+^^●^** and **iC^+^^●^**, the composition of the tautomeric mixtures and tautomeric preferences completely change in comparison to those for neutral **C** and **iC**. The favored tautomer for **C** (canonical amino-oxo form **18**) is the less important form for **C^+^^●^**, which prefers the other amino-oxo form (**38**)—the rare isomer of **C** (Figure 17). The predominant tautomer for **iC** (aromatic amino-hydroxy form **78**) becomes the rare form for **iC^+^^●^** (Figure 18), which favors the amino-oxo form (**37**). In both **C^+^^●^** and **iC^+^^●^**, two N atoms (exo N and endo N3) are favored for the two labile-protons. 

Moreover, we quantitatively proved that one-electron gain involves more dramatic changes in tautomeric equilibria than one-electron loss. Tautomers–rotamers with one labile proton at the endo C atom (exceptionally rare forms for neutral and positively ionized derivatives) become the favored isomers for all reduced derivatives: **PhOH^−^^●^**, **PhNH_2_^−^^●^**, **2HOPY^−^^●^**, **4HOPY^−^^●^**, **4HOPM^−^^●^**, **2APY^−^^●^**, **4APY^−^^●^**, **4APM^−^^●^**, **U^−^^●^**, **C^−^^●^**, and **iC^−^^●^** (Figure 14, Figure 15, Figure 16, Figure 17 and Figure 18). Exceptions take place for **2HOPM^−^^●^** and **2APM^−^^●^** containing the endo N atom at the position favored for the labile-proton in the reduced forms. 

To conclude, changes in the tautomeric preferences for neutral and ionized derivatives can only be observed in theoretical investigations carried out for the complete tautomeric mixtures. The application of one level of theory to all tautomeric derivatives offer the possibility of formulating complete conclusions on the geometric and energetic parameters for all possible isomers. Additionally, according to the LFER rules, we could deduce that the lack of linear relations between the Δ*G*s of neutral and ionized isomers, and, separately, between the HOMEDs of neutral and ionized species clearly indicates different mechanisms of positive and negative ionizations in individual tautomers-rotamers of pyrimidine bases, as well as in their model compounds.

## 10. General Remarks for Future Theoretical Investigations on Tautomeric Systems

Pyrimidine bases present in nucleic acids (**U** in **RNA**, **T** in DNA, and **C** in both RNA and DNA) form the H-bonded pairs with the corresponding purine bases (**A** and **G**, respectively). When tautomeric conversions in one base are perturbed by an external molecule, ion, radical, free electron, or other species, the base-pairing may be mismatched. Consequently, the mutation of a single nucleotide in genes may be followed by errors during DNA replication, and also during protein synthesis, particularly in the sequence of amino acids. Since these errors may lead to serious diseases, and even to death, prototropy in nucleic acid bases, as well as the influence of various internal and external factors on tautomeric-preference changes, need extensive and detailed investigations by both experimental and theoretical methods.

It is obvious that prototropy, as an exceptionally fast and reversible intramolecular movement of labile proton(s) and π-electrons, is not a simple process in nucleic acid bases. This phenomenon has already been explored in the mid-20th century and continued to greatly attract the attention of many chemists in the last twenty years. We can find hundreds of theoretical and experimental documents discussing the gaseous, micro- and macro-solvated, and, also, crystal structures of nucleobases. There are also numerous reports on their neutral, ionized, protonated, and deprotonated forms in the ground and excited states, and on non-covalently bounded adducts with other molecules or ions (including H-bond associates and metal-cation complexes), and also on their physicochemical and chemical properties. 

Most frequently, only the favored tautomer(s) have been considered, and, thus, the reported results, discussions, and conclusions are only partial (local). Although significant, they do not give the complete information on prototropy in nucleobases in different environments. Since tautomeric equilibria are very sensitive to various internal and external factors, the complete tautomeric mixtures should be investigated in the future, at least at a lower level of theory, and, next, the selected isomers can be considered at a higher level of theory. This principle concerns not only nucleic acid bases, but all tautomeric systems, particularly tautomeric nucleosides and nucleotides, new drugs and toxicants, and their homo- and hetero-associates, which can be explored in the future. Investigations limited to a few tautomeric forms and to favored tautomeric equilibria detected for neutral forms in the gas phase or in an aqueous solution cannot give the real and complete picture of the structure and biochemical properties of tautomeric systems in the presence of other species. These questions can be undertaken in future studies. They can help biochemists in understanding well the DNA mutation processes and in verifying the hypothesis of rare tautomers. They can also help to understand the pharmacology of new products considered as drugs for the reparation of the consequences of DNA mutation, and prevent unfavorable toxic effects. 

## Figures and Tables

**Table 1:** Prototropic conversions occurring for nucleic acid bases in selected tautomeric fragments.

Equilibria	Name of Conversion
>CH–C(=O)– ⇌ >C=C(–OH)–	keto-enol
>CH–C(=N–)– ⇌ >C=C(–NH–)–	imine-enamine
–NH–C(=N–)– ⇌ –N=C(–NH–)–	imine-amine (amidine)
–NH–C(=O)– ⇌ –N=C(–OH)–	amide-iminol

**Table 2:** Effects of intramolecular interactions between endo and exo groups (N-endo effects) in pyrimidine bases on stability of conformational isomers about C–OH and configurational isomers about C=NH.

N-Endo Effects (δ*G*) ^a^									
Conformational Isomers about C–OH						Configurational Isomers about C=NH			
Tautomer	2-Position		Tautomer	4-Position		Tautomer	2-Position	Tautomer	4-Position
	U ^b^	C ^c^		U ^b^	iC ^d^		iC ^d^		C ^c^
**17**	9.6	9.1, 10.0	**18**	6.7	6.8, 6.0	**18**	6.2, 7.0	**17**	4.3, 3.4
**37**	7.4	7.4, 7.4	**38**	2.5	2.2, 3.3	**38**	7.5, 6.6	**37**	2.8, 2.8
**57**	1.6	0.9, 1.7	**58**	7.5	8.0, 7.0	**58**	0.6, 0.4	**57**	5.1, 4.3
**78**	1.1, 0.0	0.7	**78**	5.3, 4.2	4.4	**13**	1.3	**13**	1.7
						**35**	5.1	**35**	0.4

^a^ The absolute δ*G* value in kcal mol^−1^, estimated as difference between the relative Gibbs energies of the corresponding isomers **a** and **b**, all calculated at the DFT(B3LYP)/6-311+G(d,p) level at 298 K. ^b^ Ref. [62]. ^c^ Ref. [64] ^d^ Ref. [65].

**Table 3:** Effects of N-endo atom(s) (δ*G*s) ^a^ on keto-enol and imine-enamine equilibria estimated for model azines when going from the parent compounds (PhOH or PhNH_2_).

Azine	Isomer	δ*G*	Azine	Isomer	δ*G*	Azine	Isomer	δ*G*
**2HOPY**	**c**	6.7	**2APY**	**c1**	2.3	**4APY**	**b2/d1**	1.4
**2HOPY**	**d**	6.9	**2APY**	**c2**	6.3	**2APM**	**c**	9.9
**4HOPY**	**b/d**	0.8	**2APY**	**d1**	2.0	**4APM**	**d1**	3.8
**2HOPM**	**c**	8.4	**2APY**	**d2**	6.8	**4APM**	**d2**	8.6
**4HOPM**	**d**	7.8	**4APY**	**b1/d2**	2.2			

^a^ In kcal mol^−1^, δ*G* as defined in the main text, estimated at the DFT(B3LYP)/6-311+G(d,p) level at 298 K according to data from refs. [18,22,49,58,59,60].

**Table 4:** Total effects of additional exo group (OH or NH_2_) and endo N atoms (δ*G*) ^a^ on stability of N1H, N3H, and C5H tautomers when going from model monosubstituted azines to disubstituted pyrimidine bases.

Model	Additional	Pyrimidine	δ*G*		
Compound	Exo Group	Base	N1H Isomer	N3H Isomer	C5H Isomer
**4HOPM**	2-OH	**U**	−1.4	−0.2	−4.7
**4APM**	2-OH	**C**	−0.6	−0.2	−2.3
**2HOPM**	4-OH	**U**	−2.5	6.4	−4.4
**2APM**	4-OH	**iC**	−2.2	5.9	−5.3
**4HOPM**	2-NH_2_	**iC**	3.4	2.9	−7.7
**2HOPM**	4-NH_2_	**C**	−2.3	4.7	−4.7

^a^ In kcal mol^−1^, δ*G* as defined in the main text, estimated at the DFT(B3LYP)/6-311+G(d,p) level at 298 K for the favored conformational and configurational isomers, according to data from refs. [49,59,60,62,64,65].

**Table 5:** Composition of isomeric mixtures for neutral model azines and their parent systems.

(a) Mono-hydroxy derivatives									
Derivative	Isomer amount (*x*_i_ in %) ^a^								Ref.
	**a1**	**a2**	**b**		**c**		**d**		
Phenol	100		4·10^−15^		4·10^−15^		4·10^−15^		[18]
**2HOPY**	18	3·10^−3^	82		3·10^−16^		1·10^−16^		[49]
**4HOPY**	88		1·10^−11^		12		1·10^−11^		[49]
**2HOPM**	81		19		8·10^−17^		19		[49]
**4HOPM**	3	5·10^−4^	97		2·10^−5^		4·10^−18^		[49]
(b) Mono-amino derivatives									
Compound	Isomer amount (*x*_i_ in %) ^a^								Ref.
	**a**		**b1**	**b2**	**c1**	**c2**	**d1**	**d2**	
Aniline	100		1·10^−19^		4·10^−18^		1·10^−19^		[22]
**2APY**	100		6·10^−11^	6·10^−9^	8·10^−20^	9·10^−23^	6·10^−21^	2·10^−24^	[58]
**4APY**	100		5·10^−21^	1·10^−20^	9·10^−11^		1·10^−20^	5·10^−21^	[58]
**2APM**	100		9·10^−19^	2·10^−13^	3·10^−28^		2·10^−13^	9·10^−19^	[59]
**4APM**	100		1·10^−10^	2·10^−8^	5·10^−12^	6·10^−15^	2·10^−24^	1·10^−25^	[60]

^a^ Calculated at the DFT(B3LYP)/6-311+G(d,p) level at 298 K.

## Data Availability

Not applicable.

## Supplementary Materials

The supplementary materials, including additional tables and figures with theoretical data, and also a list of abbreviations cited in the text, are available online at: https://www.mdpi.com/article/10.3390/molecules28217282/s1.

## References

[1] Pauling L. (1960). The Nature of the Chemical Bond.

[2] Raczyńska E.D., Kosińska W., Ośmiałowski B., Gawinecki R. (2005). Tautomeric Equilibria in Relation to pi-Electron Delocalization. Chem. Rev..

[3] Perrin C.L., Agranat I., Bagno A., Braslavsky S.E., Fernandes P.A., Gal J.-F., Lloyd-Jones G.C., Mayr H., Murdoch J.R., Nudelman N.S. (2022). Glossary of Terms Used in Physical Organic Chemistry (IUPAC Recommendations 2021). Pure Appl. Chem..

[4] Elguero J., Marzin C., Katritzky A.R., Linda P. (1976). The Tautomerism of Heterocycles (Advances in Heterocyclic Chemistry: Supplement 1).

[5] Stanovnik B., Tišler M., Katritzky A.R., Denisko O.V. (2006). The Tautomerism of Heterocycles: Substituent Tautomerism of Six-Membered Ring Heterocycles. Adv. Heterocyclic Chem..

[6] Rappoport Z. (1990). The Chemistry of Enols.

[7] Perez P., Toro-Labbe A. (2000). Characterization of Keto-Enol Tautomerism of Acetyl Derivatives from the Analysis of Energy, Chemical Potential, and Hardness. J. Phys. Chem. A.

[8] Hansen P.E. (2021). Structural Studies of β-Diketones and Their Implications on Biological Effects. Pharmaceuticals.

[9] Reichardt C. (2002). Solvent and Solvent Effects in Organic Chemistry.

[10] Lammertsma K., Prasad B.V. (1994). Imine-Enamine Tautomerism. J. Am. Chem. Soc..

[11] Fogarasi G. (2010). Studies on Tautomerism: Benchmark Quantum Chemical Calculations on Formamide and Formamidine. J. Mol. Struct..

[12] Rappoport Z. (2003). The Chemistry of Phenols.

[13] Cook M.J., Katritzky A.R., Linda P., Tack R.D. (1972). Aromatic Resonance Energies from Equilibrium Data. Tetrahedron Lett..

[14] Capponi M., Gut I.G., Hellrung B., Persy G., Wirz J. (1999). Ketonization Equilibria of Phenol in Aqueous Solution. Can. J. Chem..

[15] Zhu L., Bozzelli J.W. (2003). Kinetcs and Thermochemistry for the Gas-Phase Keto-Enol Tautomerism of Phenol↔2,4-Cyclohexadienone. J. Phys. Chem. A.

[16] Ośmiałowski B., Raczyńska E.D., Krygowski T.M. (2006). Tautomeric Equilibria and pi Electron Delocalization for some Monohydroxyarenes—Quantum Chemical Studies. J. Org. Chem..

[17] Gomez I., Rodriguez E., Reguero M. (2006). New Insights into the Interconversion Mechanism between Phenol and Its Isomers. J. Mol. Struct. (Theochem).

[18] Raczyńska E.D., Kolczyńska K., Stępniewski T.M. (2011). Tautomeric Preferences and π-Electron Delocalization for Redox Forms of Phenol. Comput. Theoret. Chem..

[19] Zeh D., Bast M., Rap D.B., Schmid P.C., Thorwith S., Brünken S., Schlemmer S., Schäfer M. (2021). Cryogenic Messenger-IR Ion Spectroscopy Study of Phenol & Aniline Molecular Ions and the Common Fragment Ion [C_5_H_6_]^+^ Formed by EI-MS. J. Mol. Spectrosc..

[20] Korth H.-G., Mulder P. (2013). Anthrone and Related Hydroxyarenes: Tautomerization and Hydrogen Bonding. J. Org. Chem..

[21] Raczyńska E.D. (2021). Quantum-Chemical Search for Keto Tautomers of Azulenols in Vacuo and Aqueous Solution. Symmetry.

[22] Raczyńska E.D., Stępniewski T.M., Kolczyńska K. (2011). Consequence of One-Electron Oxidation and One-Electron Reduction for Aniline. J. Mol. Model..

[23] Walker S.W.C., Mark A., Verbuyst B., Bogdanov B., Campbell J.L., Hopkins W.S. (2018). Characterizing the Tautomers of Protonated Aniline Using Differential Mobility Spectrometry and Mass Spectrometry. J. Phys. Chem. A.

[24] Kune C., Delvaux C., Haler J.R.N., Quinton L., Eppe G., De Pauw E., Far J. (2019). A Mechanistic Study of Protonated Aniline to Protonated Phenol Substitution Considering Tautomerization by Ion Mobility Mass Spectrometry and Tandem Mass Spectrometry. J. Am. Soc. Mass Spectrom..

[25] Lichte D., Pirkl N., Heinrich G., Goebel J.F., Koley D., Gooβen L.J. (2022). Palladium-Catalyzed *para*-C−H Arylation of Anilines with Aromatic Halides. Angew. Chem. Int. Ed. Engl..

[26] Naylor C.N., Schaefer C., Kirk A., Zimmermann S. (2023). The Origin of Izomerization of Aniline Revealed by High Kinetic Energy Ion Mobility Spectrometry (HiKE-IMS). Phys. Chem. Chem. Phys..

[27] Schleyer P.V.R., Pühlhofer F. (2002). Recommendations for the Evaluation of Aromatic Stabilization Energies. Org. Lett..

[28] Raczyńska E.D., Juras W. (2019). Effects of Ionization and Proton Transfer on Bond Length Alternation in Favored and rare isomers of Isocytosine. Comput. Theoret. Chem..

[29] Raczyńska E.D., Kamińska B. (2010). Prototropy and π-Electron Delocalization for Purine and Its Radical Ions—DFT Studies. J. Phys. Org. Chem..

[30] Raczyńska E.D., Gal J.-F., Maria P.-C., Kamińska B., Igielska M., Kurpiewski J., Juras W. (2020). Purine Tautomeric-Preferences and Bond-Length Alternation in Relation with Protonation-Deprotonation and Alkali-Metal Cationization. J. Mol. Model..

[31] Raczyńska E.D., Makowski M., Zientara-Rytter K., Kolczyńska K., Stępniewski T.M., Hallmann M. (2013). Quantum-Chemical Studies on the Favored and Rare Tautomers of Neutral and Redox Adenine. J. Phys. Chem. A.

[32] Raczyńska E.D., Kamińska B. (2022). Structural and Thermochemical Consequences of Prototropy and Ionization for the Biomolecule Xanthine in Vacuo. J. Chem. Thermodyn..

[33] Szeląg M., Raczyńska E.D. (2008). Tautomeric Equilibria for Uric Acid. Trends Org. Chem..

[34] Raczyńska E.D., Makowski M., Szeląg M., Kamińska B., Zientara K. (2010). Importance of CH Tautomers in the Tautomeric Mixture of Uric Acid. J. Mol. Struct. (Theochem).

[35] Raczyńska E.D., Makowski M., Hallmann M., Kamińska B. (2005). Geometric and Energetic Consequences of Prototropy for Adenine and Its Structural Models—A Review. RSC Adv..

[36] Watson J.D., Crick F.H.C. (1953). Genetical Implications of the Structure of Deoxyribonucleic Acid. Nature.

[37] Löwdin P.-O. (1963). Proton Tunneling in DNA and its Biological Implications. Rev. Mod. Phys..

[38] Löwdin P.-O. (1966). Quantum Genetics and the Aperiodic Solid: Some Aspects on the Biological Problems of Heredity, Mutations, Aging, and Tumors in View of the Quantum Theory of the DNA Molecule. Adv. Quantum Chem..

[39] Topal M.D., Fresco J.R. (1976). Complementary Base Pairing and the Origin of Substitution Mutations. Nature.

[40] Kwiatkowski J.S., Person W.B., Beveridge D.L., Lavery R. (1990). Theoretical Biochemistry and Molecular Biology.

[41] Leszczynski J. (1999). Computational Molecular Biology: Theoretical and Computational Chemistry.

[42] Hobza P., Šponer J. (1999). Structure, Energetics, and Dynamics of the Nucleic Acid Base Pairs. Nonempirical Ab Initio Calculations. Chem. Rev..

[43] Harańczyk M., Maciej Gutowski M. (2007). Quantum Mechanical Energy-Based Screening of Combinatorially Generated Library of Tautomers. TauTGen: A Tautomer Generator Program. J. Chem. Inf. Model..

[44] Brovarets’ O.O., Hovorun D.M. (2020). A Hidden Side of the Conformational Mobility of the Quercetin Molecule Caused by the Rotations of the O3H, O5H and O7H Hydroxyl Groups: In Silico Scrupulous Study. Symmetry.

[45] Dobrowolski J.C., Ostrowski S. (2020). The Acid-Base Through-the-Cage Interaction as an Example of an Inversion in a Cage Isomerism. Symmetry.

[46] Raczyńska E.D., Gal J.-F., Maria P.-C., Sakhawat G.S., Fahim M.Q., Saeidian H. (2022). Nitriles with High Gas-Phase Basicity—Part II Transmission of the Push-Pull Effect through Methylenecyclopropene and Cyclopropenimine Scaffolds Intercalated between Different Electron-Donor(s) and the Cyano N-Protonation Site. Molecules.

[47] Glasovac Z., Barešić L., Margetić D. (2023). A DFT Investigation of the Reactivity of Guanidinium Salts in Tandem Aza-Michael Addition/Intramolecular Cyclization. Molecules.

[48] Raczyńska E.D., Zientara K., Kolczyńska K., Stępniewski T. (2009). Change of Tautomeric Equilibria, Intramolecular Interactions and π-Electron Delocalization when Going from Phenol to Uracil. J. Mol. Struct. (Theochem).

[49] Raczyńska E.D. (2014). Electron Delocalization and Relative Stabilities for the Favored and Rare Tautomers of Hydroxyazines in the Gas Phase—A Comparison with Aminoazines. Comput. Theoret. Chem..

[50] Parr R.G., Yang W. (1989). Density Functional Theory of Atoms and Molecular Orbital Theory.

[51] Becke A.D. (1993). Density-Functional Thermochemistry. III. The Role of Exact Exchange. J. Chem. Phys..

[52] Lee C., Yang W., Parr R.G. (1988). Development of the Colle-Salvetti Correlation-Energy Formula into a Functional of the Electron Density. Phys. Rev. B.

[53] Hehre W.J., Radom L., Schleyer P.V.R., Pople J.A. (1986). Ab Initio Molecular Theory.

[54] Curtiss L.A., Raghavachari K., Trucks G.W., Pople J.A. (1991). Gaussian-2 Theory for Molecular Energies of First- and Second-Row Compounds. J. Chem. Phys..

[55] Curtiss L.A., Raghavachari K., Pople J.A. (1993). Gaussian-2 Theory Using Reduced Møller-Plesset Orders. J. Chem. Phys..

[56] Curtiss L.A., Redfern P.C., Raghavachari K. (2007). Gaussian-4 theory. J. Chem. Phys..

[57] Raczyńska E.D., Makowski M. (2018). Effects of Positive and Negative Ionization on Prototropy in Pyrimidine Bases: An Unusual Case of Isocytosine. J. Phys. Chem. A.

[58] Raczyńska E.D., Kolczyńska K., Stępniewski T.M. (2012). DFT Studies on One-Electron Oxidation and One-Electron Reduction for 2- and 4-Aminopyridines. J. Mol. Model..

[59] Raczyńska E.D. (2014). Effects of Positive and Negative Ionization for 2-Aminopyrimidine in the Gas Phase and in Water Solution. Comput. Theoret. Chem..

[60] Raczyńska E.D., Kolczyńska K., Stępniewski T.M. (2012). Consequences of One-Electron Oxidation and One-Electron Reduction for 4-Aminopyrimidine—DFT Studies. J. Mol. Model..

[61] Raczyńska E.D. (2015). Geometric and Energetic Consequences of Prototropy for Neutral and Ionized 4-Aminopyrimidine in Water Solution. Bulg. Chem. Commun..

[62] Raczyńska E.D., Zientara K., Stępniewski T.M., Kolczyńska K. (2009). Stability, Polarity, Intramolecular Interactions and π-Electron Delocalization for All Eighteen Tautomers Rotamers of Uracil. DFT Studies in the Gas Phase. Collect. Czech. Chem. Commun..

[63] Raczyńska E.D., Zientara K., Kolczyńska K., Stępniewski T.M. (2009). Change of Prototropic Equilibria for Uracil when Going from Neutral Molecule to Charged Radicals. Quantum-Chemical Studies in the Gas Phase. Polish J. Chem..

[64] Raczyńska E.D., Sapuła M., Zientara-Rytter K., Kolczyńska K., Stępniewski T.M., Hallmann M. (2016). DFT Studies on the Favored and Rare Tautomers of Neutral and Redox Cytosine. Struct. Chem..

[65] Raczyńska E.D. (2017). Quantum-Chemical Studies on the Favored and Rare Isomers of Isocytosine. Comput. Theoret. Chem..

[66] Raczyńska E.D. (2023). On Analogies in Proton-Transfers for Pyrimidine Bases in the Gas Phase (Apolar Environment)—Cytosine Versus Isocytosine. Symmetry.

[67] Raczyńska E.D., Hallmann M., Kolczyńska K., Stępniewski T.M. (2010). On the Harmonic Oscillator Model of Electron Delocalization (HOMED) Index and Its Application to Heteroatomic π-Electron Systems. Symmetry.

[68] Raczyńska E.D. (2019). Application of the Extended HOMED (Harmonic Oscillator Model of Aromaticity) Index to Simple and Tautomeric Five-Membered Heteroaromatic Cycles with C, N, O, P, and S Atoms. Symmetry.

[69] Kruszewski J., Krygowski T.M. (1972). Definition of Aromaticity Basing on the Harmonic Oscillator Model. Tetrahedron Lett..

[70] Krygowski T.M., Kruszewski J. (1974). Aromaticity of Thiophene, Pyrrole and Furan in Terms of Aromaticity Indices and Hammett σ Constants. Bull. Acad. Pol. Sci. Chim..

[71] Krygowski T.M. (1993). Crystallographic Studies of Inter- and Intramolecular Interactions Reflected in Aromatic Character of π-Electron Systems. J. Chem. Inform. Comput. Sci..

[72] Graff M., Dobrowolski J.C. (2013). On Tautomerism of Diazinones. Comput. Theoret. Chem..

[73] Kwiatkowski J.S. (1970). Electronic Absorption Spectra of 3-Hydroxypyridine and 5-Hydroxypyrimidine. Theor. Chim. Acta.

[74] Vaz da Cruz V., Büchner R., Fondell M., Pietzsch A., Eckert S., Föhlisch A. (2022). Targeting Individual Tautomers in Equilibrium by Resonant Inelastic X-ray Scattering. J. Phys. Chem. Lett..

[75] Raczyńska E.D., Gal J.-F., Maria P.-C., Zientara K., Szeląg M. (2007). Application of FT-ICR-MS for the Study of Proton-Transfer Reactions Involving Biomolecules. Anal. Bioanal. Chem..

[76] Li X., Bowen K.H., Haranczyk M., Bachorz R.A., Mazurkiewicz K., Rak J., Gutowski M. (2007). Photoelectron Spectroscopy of Adiabatically Bound Valence Anions of Rare Tautomers of the Nucleic Acid Bases. J. Chem. Phys..

[77] Stasyuk O.A., Szatylowicz H., Krygowski T.M. (2014). Tautomerisation of Thymine Acts against the Hückel 4N + 2 Rule. The Effect of Metal Ions and H-Bond Complexations on the Electronic Structure of Thymine. Org. Biomol. Chem..

[78] Liu M., Li T., Amegayibor F.S., Cardoso D.S., Fu Y., Lee J.K. (2008). Gas-Phase Thermochemical Properties of Pyrimidine Nucleobases. J. Org. Chem..

[79] Akai N., Ohno K., Aida M. (2005). Photoinduced Amino-Imino Tautomerism of 2-Aminopyridine in a Low-Temperature Argon Matrix. Chem. Phys. Lett..

[80] Beak P., Fry J.S., Lee J., Steele F. (1976). Equilibrium Studies. Protomeric Equilibria of 2- and 4-Hydroxypyridines, 2- and 4-Hydroxypyrimidines, 2- and 4-Mercaptopyridines, a Structurally Related Compounds in the Gas Phase. J. Am. Chem. Soc..

[81] Guimon C., Garrabe G., Pfister-Guillouzo G. (1979). Spectroscopie Photoelectronique a Temperature Variable Equilibre Prototropique des Hydroxyl-2 et Mercapto-2-pyridines. Tetrahrdron Lett..

[82] Nowak M.J., Lapinski L., Fulara J., Les A., Adamowicz L. (1992). Matrix-Isolation IR Spectroscopy of Tautomeric Systems and Its Theoretical Interpretation: 2-Hydroxypyridine/2(1H)-Pyridinone. J. Phys. Chem..

[83] Hatherley L.D., Brown R.D., Godfrey P.D., Pierlot A.P., Caminati W., Damiani D., Melandri S., Favero L.B. (1993). Gas-Phase Tautomeric Equilibrium of 2-Pyridinone and 2-Hydroxypyridine by Microwave Spectroscopy. J. Phys. Chem..

[84] Tanjaroon C., Subramanian R., Karunatilaka C., Kukolich S.G. (2004). Microwave Measurements of N14 and D Quadrupole Coupling for (Z)-2-Hydroxypyridine and 2-Pyridone Tautomers. J. Phys. Chem. A.

[85] Sanchez R., Giuliano B.M., Melandri S., Favero L.B., Caminati W. (2007). Gas-Phase Tautomeric Equilibrium of 4-Hydroxypyrimidine with Its Ketonic Forms: A Free Jet Millimeterwave Spectroscopy Study. J. Am. Chem. Soc..

[86] Giuliano B.M., Feyer V., Prince C.K., Coreno M., Evangelisti L., Melandri S., Caminati W. (2010). Tautomerism in 4-Hyrdoxypyrimidine, S-Methyl-2-Thiouracil and 2-Thiouracil. J. Phys. Chem. A.

[87] Lobsiger S., Frey H.M., Leutwyler S. (2010). Supersonic Jet UV Spectrum and Nonradiative Processes of the Thymine Analogue 5-Methyl-2-hydroxypyrimidine. Phys. Chem. Phys. Chem..

[88] Ośmiałowski B., Dobosz R. (2011). The Influence of Secondary Interactions on Complex Stability and Double Proton Transfer Reaction in 2-[1H]-Pyridone/2-Hydroxypyridine Dimers. J. Mol. Model..

[89] Jalbout A.F., Trzaskowski B., Xia Y., Li Y., Hu X., Li H., El-Nahas A., Adamowicz L. (2007). Structures, Stabilities and Tautomerizations of Uracil and Diphosphouracil Tautomers. Chem. Phys..

[90] Vaquero V., Sanz M.E., López J.C., Alonso J.L. (2007). The Structure of Uracil: A Laser Ablation Rotational Study. J. Phys. Chem. A.

[91] López J.C., Peña M.I., Sanz M.E., Alonso J.L. (2007). Probing Thymine with Laser Ablation Molecular Beam Fourier Transfrom Microwave Spectroscopy. J. Chem. Phys..

[92] Feyer V., Plekan O., Richter R., Coreno M., Vall-llosera G., Prince K.C., Trofimov A.B., Zaytseva I.L., Moskovskaya T.E., Gromov E.V. (2009). Tautomerism in Cytosine and Uracil: An Experimental and Theoretical Core Level Spectroscopic Study. J. Phys. Chem. A.

[93] Fujii M., Tamura T., Mikami N., Ito M. (1986). Electronic Spectra of Uracil in a Supersonic Jet. Chem. Phys. Lett..

[94] Tsuchiya Y., Tamura T., Fujii M., Ito M. (1988). Keto-Enol Tautomer of Uracil and Thymine. J. Phys. Chem..

[95] McClure R.J., Craven B.M. (1973). New Investigations of Cytosine and its Monohydrate. Acta Crystallogr. Sect. B.

[96] Dreyfus M., Bensaude O., Dodin G., Dubois J.E. (1976). Tautomerism in Cytosine and 3-Methylcytosine. A Thermodynamic and Kinetic Study. J. Am. Chem. Soc..

[97] Trygubenko S.A., Bogdan T.V., Rueda M., Orozca M., Luque F.J., Šponer J., Slavíček P., Hobza P. (2002). Correlated Ab Initio Study of Nucleic Acid Bases and Their Tautomers in the Gas Phase, in a Microhydrated Environment and in Aqueous Solution. Pant 1. Cytosine. Phys. Chem. Chem. Phys..

[98] Ai H., Liu J., Chan K. (2013). Stability and Isomerization of Complexes Formed by Metal Ions and Cytosine Isomers in Aqueous Phase. J. Mol. Model..

[99] Nir E., Müller M., Grace L.I., de Vries M.S. (2002). REMPI Spectroscopy of Cytosine. Chem. Phys. Lett..

[100] Nir E., Huenig I., Kleinermanns K., de Vries M.S. (2003). The Nucleobase Cytosine and the Cytosine Dimer Investigated by Double Resonance Laser Spectroscopy and Ab Initio Calculations. Phys. Chem. Phys. Chem..

[101] Choi M.Y., Dong F., Miller R.E. (2005). Multiple Tautomers of Cytosine Identified and Characterized by Infrared Laser Spectroscopy in Helium Nanodroplets: Probing Structure Using Vibrational Transition Moment Angles. Phil. Trans. R. Soc. A.

[102] Bazsó G., Tarczay G., Fogarasi G., Szalay P.G. (2011). Tautomers of Cytosine and Their Excited Electronic States: A Matrix Isolation Spectroscopic and Quantum Chemical Study. Phys. Chem. Chem. Phys..

[103] Alonso J.L., Vaquero V., Peña I., López J.C., Mata S., Caminati W. (2013). All Five Forms of Cytosine Revealed in the Gas Phase. Ang. Chem. Int. Ed..

[104] Portalone G., Colapietro M. (2007). Redetermination of Isocytosine. Acta Crystallogr. Sect. E Struct. Rep. Online.

[105] Dracínský M., Jansa P., Ahonen K., Budesínský M. (2011). Tautomerism and the Protonation/Deprotonation of Isocytosine in Liquid- and Solid-States Studied by NMR Spectroscopy and Theoretical Calculations. Eur. J. Org. Chem..

[106] Ivanov A.Y., Stepanian S.G., Adamowicz L. (2012). Tautomeric Transitions of Isocytosine Isolated in Argon and Neon Matrices Induced by UV Irradiation. J. Mol. Struct..

[107] Bakalska R.I., Delchev V.B. (2012). Comparative Study of the Relaxation Mechanisms of the Excited States of Cytosine and Isocytosine. J. Mol. Model..

[108] Szabla R., Góra R.W., Šponer J. (2016). Ultrafast Excited-State Dynamics of Isocytosine. Phys. Chem. Chem. Phys..

[109] Choi J., Yang C., Fujitsuka M., Tojo S., Ihee H., Majima T. (2015). Proton Transfer of Guanine Radical Cations Studied by Time-Resolved Resonance Raman Spectroscopy Combined with Pulse Radiolysis. J. Phys. Chem. Lett..

[110] Huang S.R., Dang A., Turećek F. (2020). Ground and Excited States of Gas-Phase DNA Nucleobase Cation-Radicals. A UV-vis Photodisociation Action Spectroscopy and Computational Study of Adenine and 9-Methyladenine. J. Am. Soc. Mass Spectrom..

[111] Le H.T., Flammang R., Gerbaux P., Bouchoux G., Nguyen M.T. (2001). Ionized Phenol and Its Isomers in the Gas Phase. J. Phys. Chem. A.

[112] Trupia L., Dechamps N., Flammang R., Bouchoux G., Nguyen M.T., Gerbaux P. (2008). Isomeric Recognition by Ion/Molecule Reactions: The Ionized Phenol-Cyclohexadienone Case. J. Am. Soc. Mass Spectrom..

[113] Le H.T., Flammang R., Barbieux-Flammang M., Gerbaux P., Nguyen M.T. (2002). Ionized Aniline and Its Distonic Radical Cation Isomers. Int. J. Mass Spectrom..

[114] Ozeki K., Cockett M.C.R., Okuyama K., Takahashi M., Kimura K. (1995). Structural Isomers and Tautomerism of 2-Hydroxypyridine in the Cation Ground State Studied by Zero Kinetic Energy (ZEKE) Photoelectron Spectroscopy. J. Phys. Chem..

[115] Tureček F., Wolken J.K. (2001). Energetics of Uracil Cation Radical and Anion Radical Ion-Molecule Reactions in the Gas Phase. J. Phys. Chem. A.

[116] Lamsabhi A.M., Gutiérrez-Oliva S., Mó O., Toro-Labbé A., Yáñez M. (2015). Effects of the Ionization in the Tautomerism of Uracil: A Reaction Electronic Flux Perspective. J. Comput. Chem..

[117] Bachorz R.A., Klopper W., Gutowski M., Li X., Kit H., Bowen K.H. (2008). Photoelectron Spectrum of Valence Anions of Uracil and First-Principles Calculations of Excess Electron Binding Energies. J. Chem. Phys..

[118] Dang A., Nguyen H.T.H., Ruiz H., Piacentino E., Ryzhov V., Tureček F. (2018). Experimental Evidence for Noncanonical Thymine Cation Radicals in the Gas Phase. J. Phys. Chem. B.

[119] Mazurkiewicz K., Bachorz R.A., Gutowski M., Rak J. (2006). On the Unusual Stability of Valence Anions of Thymine Based on Very Rare Tautomers: A Computational Study. J. Phys. Chem. B.

[120] Wolken J.K., Yao C., Tureček F., Polce M.J., Wesdemiotis C. (2007). Cytosine Neutral Molecules and Cation-Radicals in the Gas-Phase: Structures, Energies, Ion Chemistry, and Neutralization-Reionization Mass Spectrometry. Int. J. Mass Spectrom..

[121] Lesslie M., Lawler J.T., Dang A., Korn J.A., Bím D., Steinmetz V., Maître P., Tureček F., Ryzhov V. (2017). Cytosine Radical Cations: A Gas-Phase Study Combining IRMPD Spectroscopy, UVPD Spectroscopy, Ion-Molecule Reactions, and Theoretical Calculations. Chem. Phys. Chem..

[122] Molina F., Dezalay J., Soorkia S., Broquier M., Hochlaf M., Pino G.A., Grégoire G. (2022). Cryogenic IR and UV Spectroscopy of Isomer-Selected Ctosine Radical Cation. Phys. Chem. Chem. Phys..

[123] Tureček F. (2021). Flying DNA Cation Radicals in the Gas Phase: Generation and Action Spectroscopy of Canonical and Noncanonical Nucleobase Forms. J. Phys. Chem. B.

[124] Schuchmann M.N., Naumov S., Schuchmann H.-P., von Sonntag J., von Sonntag C. (2005). 4-Amino-3H-Pyrimidin-2-one (‘Isocytosine’) Is a Short-Lived Non-Radical Intermediate Formed in the Pulse Radiolysis of Cytosine in Aqueous Solution. Radiat. Phys. Chem..

[125] Herak J.N., Rakvin B., Bytyci M. (1979). ESR Study of the Ionic Radical Species in an Irradiated Single Crystal of Isocytosine. J. Magn. Reson..

