# Supplementary material for: On Prototropy and Bond Length Alternation in Neutral and Ionized Pyrimidine Bases and Their Model Azines in Vacuo
Source: Molecules. 2023 Oct 26;28(21):7282. doi: 10.3390/molecules28217282 (PMC10648772; doi:10.3390/molecules28217282)
Supplement: Supplementary file 1 [file molecules-28-07282-s001.zip › molecules-2657997-supplementary.pdf]

Supplementary Materials

On Prototropy and Bond-Length Alternation in Neutral and Ionized Pyrimidine Bases and Their Model Azines in Vacuo

Ewa D. Raczyńska

Department of Chemistry, Warsaw University of Life Sciences (SGGW),

ul. Nowoursynowska 159c, 02-776 Warszawa, Poland,

ewa_raczyńska@sggw.edu.pl; Tel.: +48-22-59-37623

**Contents**	**Page**
Relative Gibbs energies for neutral and ionized tautomers-rotamers of phenol and mono-hydroxy azines (Table S1)	S2
Relative Gibbs energies for neutral and ionized tautomers-rotamers of aniline and mono-amino azines (Table S2)	S3
Relative Gibbs energies for neutral and ionized tautomers-rotamers of pyrimidine bases (Table S3)	S4
HOMED indices for neutral and ionized tautomers-rotamers of phenol and mono-hydroxy azines (Table S4)	S6
HOMED indices for neutral and ionized tautomers-rotamers of aniline and mono-amino azines (Table S5)	S7
HOMED indices for neutral and ionized tautomers-rotamers of pyrimidine bases (Table S6)	S8
Composition of isomeric mixtures for neutral mono-hydroxy azines estimated at the G2 level (Table S7)	S10
Twenty-one tautomers-rotamers possible for isocytosine (Figure S1)	S11
Plots between Δ*G*s of all tautomers-rotamers of neutral pyrimidine bases (Figure S2)	S12
Plots between HOMED6s and Δ*G*s of all tautomers-rotamers of neutral mono-hydroxy model compounds and pyrimidine bases (Figure S3)	S13
Plots between HOMED8s and Δ*G*s of all tautomers-rotamers of neutral pyrimidine bases (Figure S4)	S14
Lack of linear relations between reformulated HOMAs and HOMEDs of all tautomers-rotamers of neutral pyrimidine bases (Figure S5)	S15
Plots between Δ*G*s of neutral and ionized tautomers-rotamers of pyrimidine bases (Figure S6)	S16
Plots between HOMEDs of neutral and ionized tautomers-rotamers of pyrimidine bases (Figure S7)	S17
List of abbreviations applied in the main text	S18

Table S1. Relative Gibbs energies (Δ*G*)[a] for neutral, positively and negatively ionized tautomers-rotamers of phenol and mono-hydroxy aromatic azines.

Compound	Isomer	Δ*G*		
		**Neutral**	**Radical cation**	**Radical anion**
Phenol[b]	**a**	0.0	0.0	0.0
	**b/d**	16.7	26.6	−19.8
	**c**	16.3	31.6	−11.1
2-Hydroxypyridine[c]	**2HOPY-a1**	0.0	0.0	0.0
	**2HOPY-a2**	5.2	6.3	5.2
	**2HOPY-b**	−0.9	−11.0	−11.4
	**2HOPY-c**	23.0	30.7	−1.0
	**2HOPY-d**	23.6	30.0	−13.7
4-Hydroxypyridine[c]	**4HOPY-a**	0.0	0.0	0.0
	**4HOPY-b/d**	17.5	23.8	−20.3
	**4HOPY-c**	1.2	−11.4	−6.9
2-Hydroxypyrimidine[c]	**2HOPM-a1/a2**	0.0	0.0	0.0
	**2HOPM-b/d**	0.9	−7.9	−10.3
	**2HOPM-c**	24.7	17.9	3.0
4-Hydroxypyrimidine[c]	**4HOPM-a1**	0.0	0.0	0.0
	**4HOPM-a2**	5.2	4.0	4.9
	**4HOPM-b**	−2.1	−17.2	−12.3
	**4HOPM-c**	7.0	−9.7	5.2
	**4HOPM-d**	24.5	15.0	−14.0

[a] Δ*G* (in kcal mol^{-1}) refers to difference between G of **a-d** and that of **a** (or **a1**) estimated at the DFT(B3LYP)/6-311+G(d,p) level at 298.15 K. [b] As in ref. [18]. [c] As in ref. [49].

Table S2. Relative Gibbs energies (ΔG)[a] for neutral, positively and negatively ionized tautomers-rotamers of aniline and mono-amino aromatic azines.

Compound	Isomer	ΔG		
		**Neutral**	**Radical cation**	**Radical anion**
Aniline[b]	**a**	0.0	0.0	0.0
	**b1/d2**	28.2	43.1	−4.7
	**b2/d1**	28.4	43.0	−3.4
	**c**	26.5	47.9	4.6
2-Aminopyridine[c]	**2APY-a**	0.0	0.0	0.0
	**2APY-b1**	16.7	7.5	5.3
	**2APY-b2**	13.9	3.9	2.0
	**2APY-c1**	28.8	50.2	7.9
	**2APY-c2**	32.8	51.2	11.6
	**2APY-d1**	30.4	50.4	−4.2
	**2APY-d2**	35.0	52.9	0.6
4-Aminopyridine[c]	**4APY-a**	0.0	0.0	0.0
	**4APY-b1/d2**	30.4	39.8	−3.4
	**4APY-b2/d1**	29.8	39.8	−4.6
	**4APY-c**	16.4	−1.9	8.3
2-Aminopyrimidine[d]	**2APM-a**	0.0	0.0	0.0
	**2APM-b1/d2**	24.1	14.7	10.2
	**2APM-b2/d1**	17.4	6.5	3.5
	**2APM-c**	36.4	44.0	15.6
4-Aminopyrimidine[e]	**4APM-a**	0.0	0.0	0.0
	**4APM-b1**	16.2	4.8	4.6
	**4APM-b2**	13.3	1.5	1.5
	**4APM-c1**	18.2	1.8	14.3
	**4APM-c2**	22.2	6.2	18.0
	**4APM-d1**	32.2	42.4	−4.9
	**4APM-d2**	36.8	43.0	−0.3

[a] ΔG (in kcal mol^{-1}) refers to difference between G of **a**-**d** and that of **a** estimated at the DFT(B3LYP)/6-311+G(d,p) level at 298.15 K. [b] As in ref. [22]. [c] As in ref. [58]. [d] As in ref. [59]. [e] As in refs [60,61].

Table S3. Relative Gibbs energies (Δ*G*)[a] for neutral, positively and negatively ionized tautomers-rotamers of pyrimidine bases.

		Δ*G*		
**Compound**	**Isomer**	**Neutral**	**Radical cation**	**Radical anion**
Uracil[b]	**U13**	–13.9	–11.5	–22.8
	**U17a**	15.2	9.8	7.1
	**U17b**	5.6	0.9	5.8
	**U18a**	–1.6	–4.8	–11.3
	**U18b**	5.1	1.8	–5.0
	**U35**	2.7	25.3	–24.9
	**U37a**	–2.3	–9.0	–6.8
	**U37b**	5.1	–1.7	nf
	**U38a**	9.8	0.9	–4.2
	**U38b**	7.3	–0.5	–4.2
	**U57a**	21.4	24.2	–12.6
	**U57b**	19.8	24.2	–14.1
	**U58a**	20.3	23.0	–1.7
	**U58b**	27.8	29.3	4.6
	**U78aa**	0.0	0.0	0.0
	**U78ab**	5.3	6.8	6.3
	**U78ba**	1.1	0.6	1.0
	**U78bb**	5.3	7.9	5.7
Cytosine[c]	**C13a**	2.5	2.4	–4.9
	**C13b**	0.8	1.3	–7.9
	**C17aa**	26.7	14.5	15.8
	**C17ab**	31.0	18.1	20.1
	**C17ba**	17.6	5.8	18.0
	**C17bb**	21.0	9.4	20.7
	**C18**	–1.4	0.2	–10.4
	**C35a**	17.7	nf	–11.3
	**C35b**	17.3	37.2	–11.9
	**C37aa**	15.9	5.9	10.0
	**C37ab**	13.1	3.1	7.8
	**C37ba**	23.3	14.6	13.5

	**C37bb**	20.5	10.8	nf
	**C38**	5.6	–0.4	–7.3
	**C57aa**	30.8	38.6	–2.9
	**C57ab**	35.9	nf	1.9
	**C57ba**	29.9	39.2	–3.6
	**C57bb**	34.2	nf	0.4
	**C58**	20.0	25.2	–1.3
	**C78a**	0.0	0.0	0.0
	**C78b**	0.7	0.4	0.9
Isocytosine[d]	**iC13a**	7.8	14.5	–4.5
	**iC13b**	6.5	11.6	–5.6
	**iC17**	10.4	17.0	1.9
	**iC18aa**	21.4	14.1	8.4
	**iC18ab**	28.2	20.0	14.8
	**iC18ba**	15.2	7.0	2.6
	**iC18bb**	21.2	12.0	8.2
	**iC35a**	15.0	37.8	–11.9
	**iC35b**	20.1	nf	–5.8
	**iC37**	0.8	–1.6	–8.8
	**iC38aa**	25.7	15.3	9.1
	**iC38ab**	23.3	12.8	nf
	**iC38ba**	33.2	24.0	15.2
	**iC38bb**	29.9	20.6	nf
	**iC57**	16.8	26.7	–15.1
	**iC58aa**	31.1	44.2	10.7
	**iC58ab**	39.1	50.8	17.0
	**iC58ba**	31.7	44.6	12.0
	**iC58bb**	38.7	50.8	17.4
	**iC78a**	0.0	0.0	0.0
	**iC78b**	4.4	5.4	4.5

[a] Δ*G* (in kcal mol^{-1}) for **U**, **C**, and **iC** relative to G of **U78aa**, **C78a**, and **iC78a**, respectively, estimated at the DFT(B3LYP)/6-311+G(d,p) level at 298.15 K (nf – structure not found). [b] As in refs [62,63]. [c] As in ref. 64]. [d] As in refs [28,57,65].

Table S4. HOMED6[a,b] and HOMED7[b,c] indices for neutral, positively and negatively ionized tautomers-rotamers of phenol and mono-hydroxy aromatic azines.

Compound	Isomer	Neutral		Radical cation		Radical anion	
		HOMED6	HOMED7	HOMED6	HOMED7	HOMED6	HOMED7
Phenol[d]	**a**	0.999_7	0.920	0.918	0.927	0.994	0.916
	**b/d**	0.374	0.470	0.518	0.606	0.455	0.567
	**c**	0.403	0.498	0.613	0.685	0.503	0.609
2-HO-pyridine[e]	**2HOPY-a1**	0.995	0.943	0.919	0.933	0.880	0.782
	**2HOPY-a2**	0.996	0.935	0.908	0.922	0.908	0.802
	**2HOPY-b**	0.803	0.812	0.808	0.807	0.745	0.792
	**2HOPY-c**	0.332	0.425	−0.023	0.083	0.477	0.586
	**2HOPY-d**	0.342	0.428	0.166	0.233	0.432	0.541
4- HO-pyridine[e]	**4HOPY-a**	0.999	0.937	0.979	0.970	0.972	0.907
	**4HOPY-b/d**	0.358	0.454	0.305	0.394	0.483	0.587
	**4HOPY-c**	0.754	0.782	0.944	0.955	0.832	0.855
2- HO-pyrimidine[e]	**2HOPM-a1/a2**	1.000	0.961	0.990	0.989	0.856	0.776
	**2HOPM-b/d**	0.785	0.744	0.788	0.789	0.730	0.774
	**2HOPM-c**	0.259	0.350	0.087	0.154	0.454	0.558
4- HO-pyrimidine[e]	**4HOPM-a1**	0.995	0.957	0.983	0.977	0.870	0.790
	**4HOPM-a2**	0.996	0.950	0.983	0.977	0.892	0.801
	**4HOPM-b**	0.774	0.782	0.824	0.823	0.731	0.778
	**4HOPM-c**	0.681	0.706	0.937	0.949	0.795	0.816
	**4HOPM-d**	0.313	0.401	0.060	0.153	0.491	0.584

[a] HOMED6 for six-membered ring (six bonds). [b] Calculated for structures optimized at the DFT(B3LYP)/6-311+G(d,p) level. [c] HOMED7 for entire molecule (eight bonds). [d] Data taken from ref. [18]. [e] Data taken from ref. [49].

Table S5. HOMED6[a,b] and HOMED7[b,c] indices for neutral, positively and negatively ionized tautomers-rotamers of aniline and mono-amino aromatic azines.

Compound	Isomer	Neutral		Radical cation		Radical anion	
		HOMED6	HOMED7	HOMED6	HOMED7	HOMED6	HOMED7
Aniline[d]	**a**	0.998	0.948	0.926	0.942	0.994	0.952
	**b1/d2**	0.384	0.486	0.711	0.760	0.490	0.597
	**b2/d1**	0.353	0.459	0.670	0.725	0.470	0.582
	**c**	0.413	0.511	0.680	0.747	0.514	0.595
2-H_2N-pyridine[e]	**2APY-a**	0.994	0.996	0.929	0.944	0.923	0.846
	**2APY-b1**	0.767	0.791	0.913	0.925	0.714	0.772
	**2APY-b2**	0.802	0.818	0.927	0.937	0.786	0.829
	**2APY-c1**	0.356	0.453	0.669	0.721	0.535	0.605
	**2APY-c2**	0.352	0.454	0.722	0.779	0.486	0.595
	**2APY-d1**	0.365	0.453	0.697	0.755	0.500	0.599
	**2APY-d2**	0.324	0.424	0.200	0.266	0.448	0.560
4-H_2N-pyridine[e]	**4APY-a**	0.996	0.969	0.947	0.958	0.983	0.956
	**4APY-b1/d2**	0.334	0.440	0.587	0.659	0.498	0.602
	**4APY-b2/d1**	0.373	0.470	0.683	0.742	0.520	0.618
	**4APY-c**	0.744	0.779	0.936	0.949	0.820	0.849
2-H_2N-pyrimidine[f]	**2APM-a**	0.995	0.986	0.939	0.951	0.870	0.812
	**2APM-b1/d2**	0.743	0.760	0.925	0.917	0.701	0.758
	**2APM-b2/d1**	0.790	0.795	0.945	0.955	0.779	0.819
	**2APM-c**	0.298	0.397	0.173	0.248	0.489	0.596
4-H_2N-pyrimidine[g]	**4APM-a**	0.991	0.981	0.950	0.960	0.904	0.843
	**4APM-b1**	0.737	0.756	0.924	0.936	0.712	0.767
	**4APM-b2**	0.707	0.736	0.938	0.949	0.775	0.818
	**4APM-c1**	0.664	0.701	0.917	0.934	0.777	0.805
	**4APM-c2**	0.663	0.702	0.921	0.936	0.773	0.804
	**4APM-d1**	0.346	0.435	0.122	0.226	0.557	0.640
	**4APM-d2**	0.170	0.297	0.106	0.210	0.470	0.574

[a] HOMED6 for six-membered ring (six bonds). [b] Calculated for structures optimized at the DFT(B3LYP)/6-311+G(d,p) level. [c] HOMED7 for entire molecule (eight bonds). [d] Data from ref. [22]. [e] Data from ref. [58]. [f] Data from ref. [59]. [g] Data from refs [60,61].

Table S6. HOMED6[a,b] and HOMED8[b,c] indices for neutral, positively and negatively ionized tautomers-rotamers of pyrimidine bases.

		Neutral		Radical cation		Radical anion	
**Compound**	**Isomer**	**HOMED6**	**HOMED8**	**HOMED6**	**HOMED8**	**HOMED6**	**HOMED8**
Uracil[d]	**U13**	0.698	0.688	0.571	0.553	0.567	0.659
	**U17a**	0.650	0.649	0.913	0.924	0.773	0.777
	**U17b**	0.741	0.728	0.933	0.942	0.770	0.762
	**U18a**	0.788	0.762	0.789	0.796	0.763	0.701
	**U18b**	0.770	0.738	0.836	0.839	0.768	0.684
	**U35**	0.342	0.402	0.464	0.521	0.336	0.469
	**U37a**	0.769	0.755	0.807	0.806	0.600	0.592
	**U37b**	0.754	0.739	0.785	0.787	nf	nf
	**U38a**	0.771	0.747	0.709	0.720	0.748	0.680
	**U38b**	0.788	0.758	0.765	0.768	0.749	0.681
	**U57a**	0.337	0.429	0.194	0.323	0.500	0.515
	**U57b**	0.384	0.466	0.200	0.331	0.496	0.515
	**U58a**	0.281	0.370	−0.017	0.116	0.416	0.460
	**U58b**	0.217	0.320	0.032	0.155	0.380	0.413
	**U78aa**	0.995	0.926	0.893	0.914	0.859	0.690
	**U78ab**	0.994	0.918	0.875	0.898	0.880	0.691
	**U78ba**	0.996	0.925	0.903	0.924	0.843	0.674
	**U78bb**	0.996	0.920	0.984	0.976	0.864	0.678
Cytosine[e]	**C13a**	0.688	0.690	0.727	0.714	0.582	0.670
	**C13b**	0.709	0.706	0.745	0.730	0.634	0.708
	**C17aa**	0.629	0.636	0.916	0.925	0.760	0.772
	**C17ab**	0.622	0.633	0.915	0.920	0.753	0.766
	**C17ba**	0.671	0.680	0.924	0.933	0.762	0.762
	**C17bb**	0.671	0.683	0.930	0.935	0.762	0.757
	**C18**	0.785	0.791	0.850	0.866	0.756	0.703
	**C35a**	0.351	0.415	nf	nf	0.363	0.494
	**C35b**	0.331	0.402	0.150	0.276	0.381	0.507
	**C37aa**	0.734	0.731	0.902	0.912	0.625	0.611
	**C37ab**	0.777	0.767	0.923	0.931	0.704	0.674
	**C37ba**	0.714	0.752	0.878	0.890	0.798	0.797

	**C37bb**	0.768	0.755	0.902	0.912	nf	nf
	**C38**	0.799	0.794	0.749	0.754	0.727	0.692
	**C57aa**	0.351	0.439	0.647	0.734	0.548	0.550
	**C57ab**	0.296	0.400	nf	nf	0.509	0.523
	**C57ba**	0.395	0.473	0.650	0.737	0.549	0.553
	**C57bb**	0.344	0.438	nf	nf	0.512	0.529
	**C58**	0.267	0.391	−0.017	0.129	0.434	0.501
	**C78a**	0.990	0.943	0.896	0.917	0.853	0.660
	**C78b**	0.992	0.944	0.912	0.929	0.849	0.662
Isocytosine[f]	**iC13a**	0.700	0.695	0.717	0.714	0.571	0.662
	**iC13b**	0.705	0.701	0.715	0.706	0.568	0.660
	**iC17**	0.694	0.710	0.896	0.920	0.792	0.810
	**iC18aa**	0.757	0.744	0.928	0.939	0.726	0.680
	**iC18ab**	0.723	0.706	0.925	0.934	0.726	0.658
	**iC18ba**	0.795	0.771	0.947	0.952	0.785	0.721
	**iC18bb**	0.777	0.747	0.945	0.948	0.788	0.703
	**iC35a**	0.371	0.437	0.428	0.491	0.422	0.542
	**iC35b**	0.355	0.426	nf	nf	0.365	0.501
	**iC37**	0.810	0.795	0.794	0.794	0.682	0.675
	**iC38aa**	0.796	0.773	0.902	0.913	0.787	0.717
	**iC38ab**	0.803	0.777	0.931	0.937	nf	nf
	**iC38ba**	0.733	0.722	0.864	0.883	0.728	0.672
	**iC38bb**	0.753	0.737	0.906	0.917	nf	nf
	**iC57**	0.361	0.476	0.179	0.326	0.504	0.548
	**iC58aa**	0.327	0.414	0.634	0.722	0.469	0.511
	**iC58ab**	0.272	0.362	0.561	0.666	0.432	0.463
	**iC58ba**	0.331	0.418	0.636	0.724	0.459	0.502
	**iC58bb**	0.280	0.369	0.580	0.680	0.423	0.456
	**iC78a**	0.994	0.946	0.919	0.930	0.867	0.715
	**iC78b**	0.991	0.936	0.916	0.924	0.903	0.740

[a] HOMED6 for six-membered ring (six bonds). [b] Calculated for structures optimized at the DFT(B3LYP)/6-311+G(d,p) level (nf – structure not found). [c] HOMED8 for entire molecule (eight bonds). [d] Data from refs [62,63]. [e] Data from ref. [64]. [f] Data from refs [28,57,65].

Table S7. Composition of isomeric mixtures for neutral mono-hydroxy aromatic azines estimated at the G2 level.

Derivative	Amount of isomer (x_i in %)					Ref.
	**a1**	**a2**	**b**	**c**	**d**	
**2HOPY**	73[b]	$2\ 10^{-2}$	27	$1\ 10^{-14}$	$2\ 10^{-15}$	[49]
**4HOPY**	99		$1\ 10^{-11}$	1	$1\ 10^{-11}$	[49]
**2HOPM**	99		1	$5\ 10^{-15}$	1	[49]
**4HOPM**	20	$1\ 10^{-2}$	80	$5\ 10^{-5}$	$9\ 10^{-16}$	[49]

Figure S1. Twenty-one tautomers-rotamers possible for **iC**. Two labile protons indicated in bold red color.

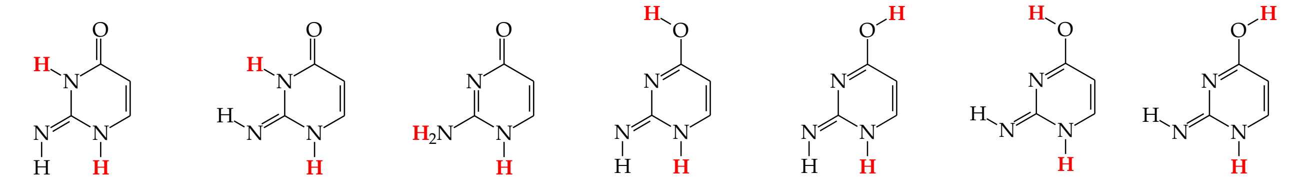

13a **13b** **17** **18aa** **18ab** **18ba** **18bb**

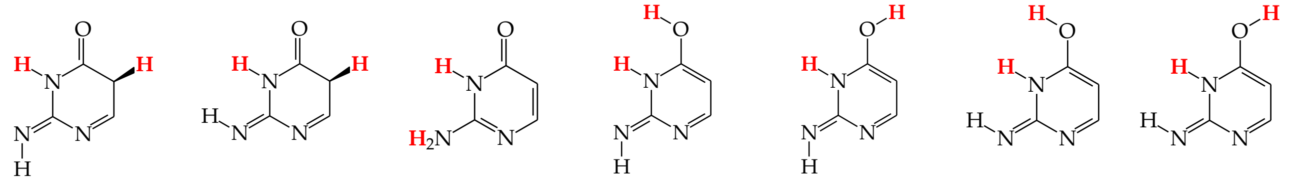

35a **35b** **37** **38aa** **38ab** **38ba** **38bb**

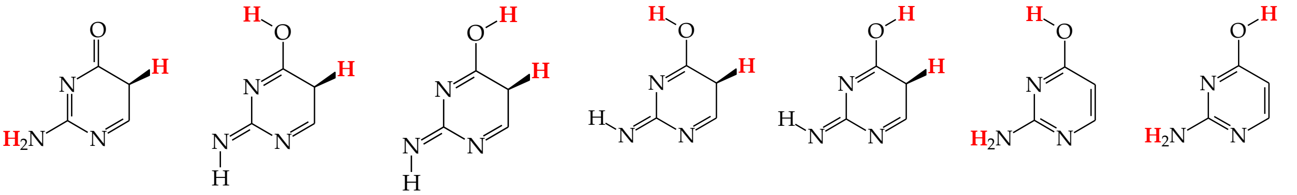

57 **58aa** **58ab** **58ba** **58bb** **78a** **78b**

Figure S2. Plots between ΔGs of all tautomers-rotamers of neutral pyrimidine bases. DFT data taken from refs [62,64,65].

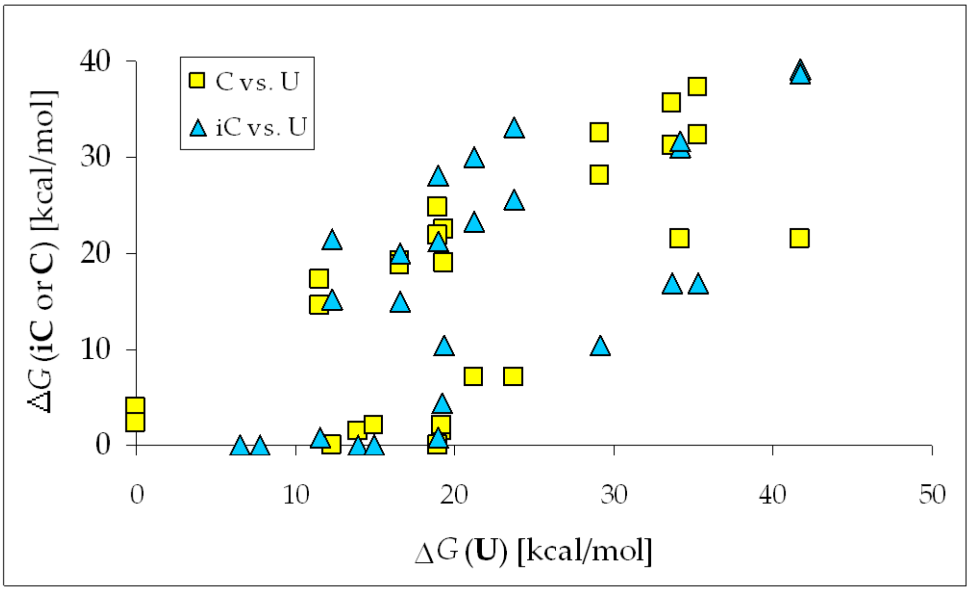

Figure S3. Plots between HOMED6s and ΔGs of all tautomers-rotamers of mono-hydroxy aromatic azines (including phenol) and uracil containing one and two exo OH, respectively (a), and also for all isomers of cytosine and isocytosine containing one exo OH and one exo NH_2 (b). DFT data taken from refs [18,44,62,64,65].

(a) Mono-hydroxy azines (including phenol) and uracil

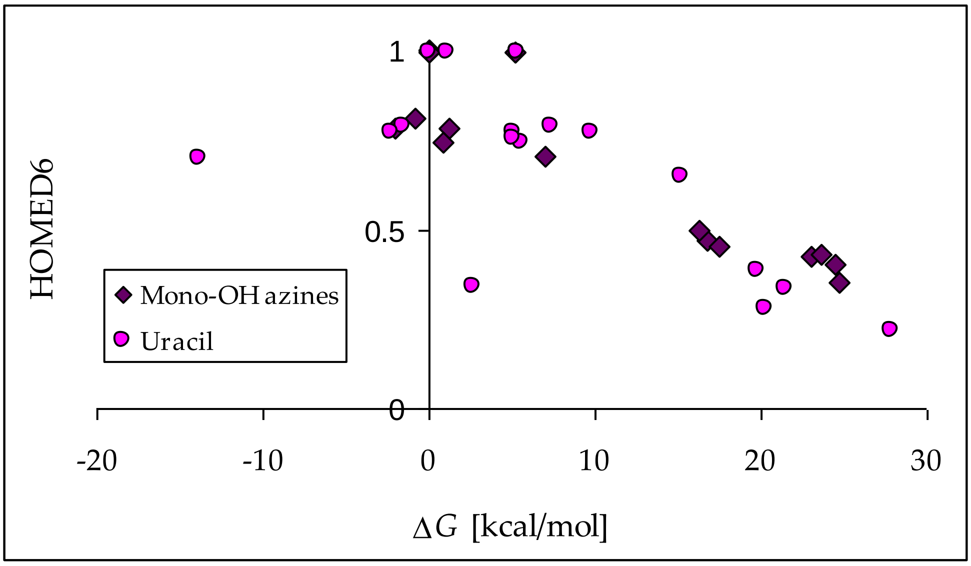

(b) Cytosine and isocytosine

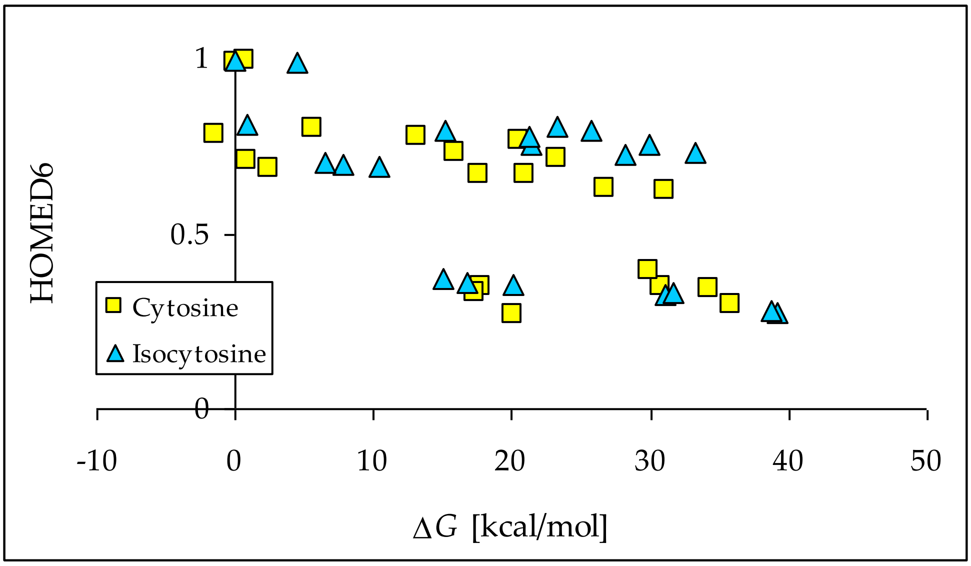

Figure S4. Plots between HOMED8s and Δ*G*s of all tautomers-rotamers of neutral pyrimidine bases. DFT data taken from refs [62,64,65].

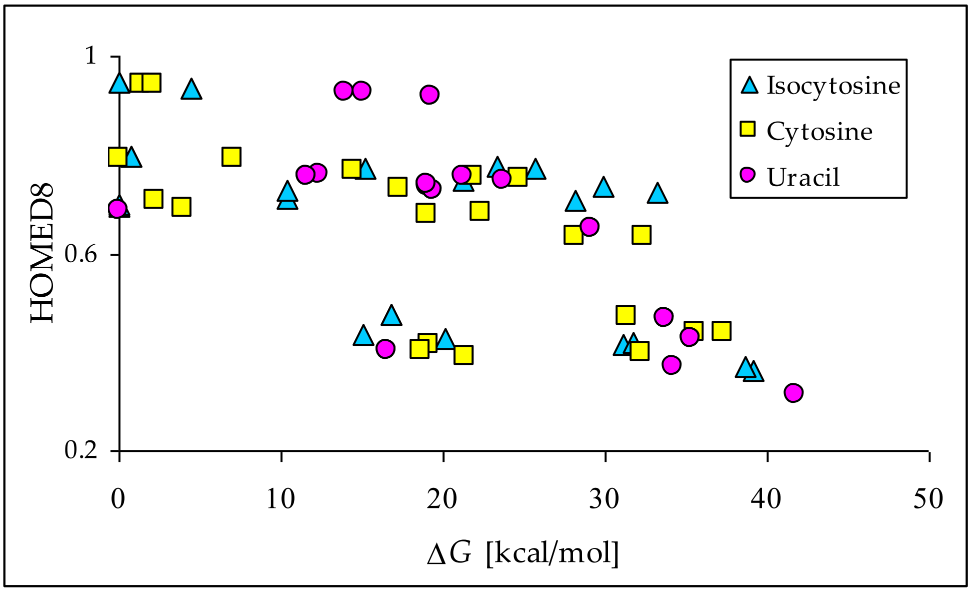

Figure S5. Lack of linear relations between the reformulated HOMA and HOMED indices estimated for all tautomers-rotamers of neutral pyrimidine bases in the six-membered ring (a) and in the entire molecule (b). DFT-calculated geometric parameters taken from refs [65,68].

(a) Six-membered ring

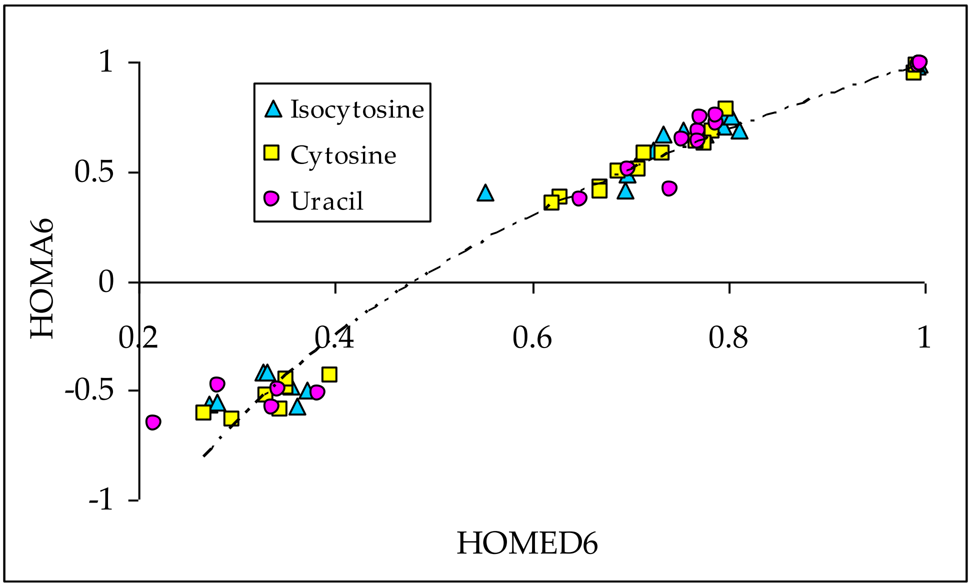

(b) Entire molecule

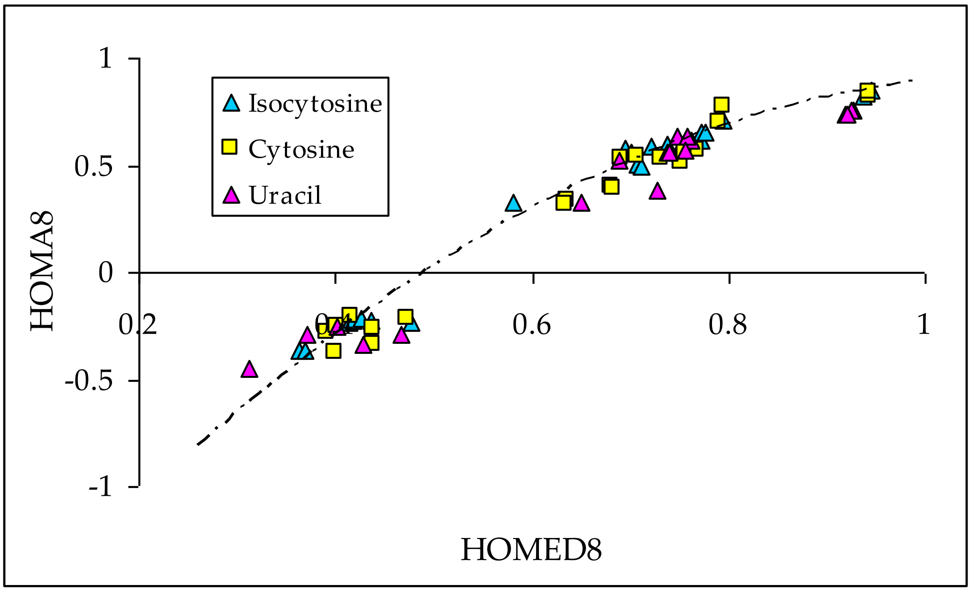

Figure S6. Plots between ΔGs of neutral and ionized tautomers-rotamers of uracil (a), cytosine (b), and isocytosine (c). DFT data taken from refs [28,57,62,63,64,65].

(a) Uracil

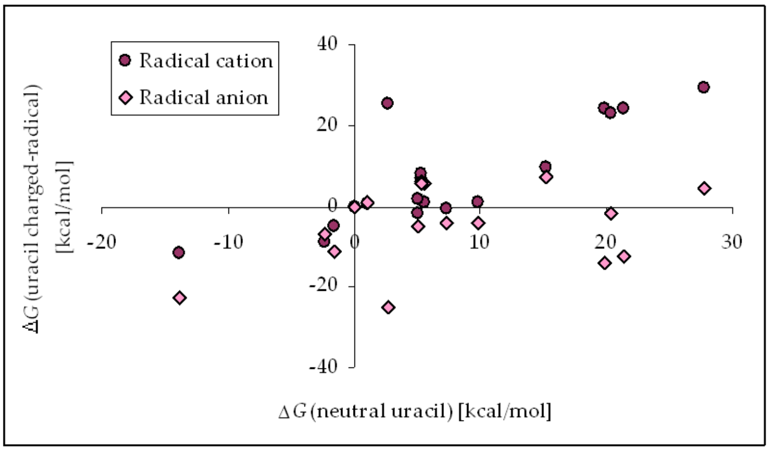

(b) Cytosine

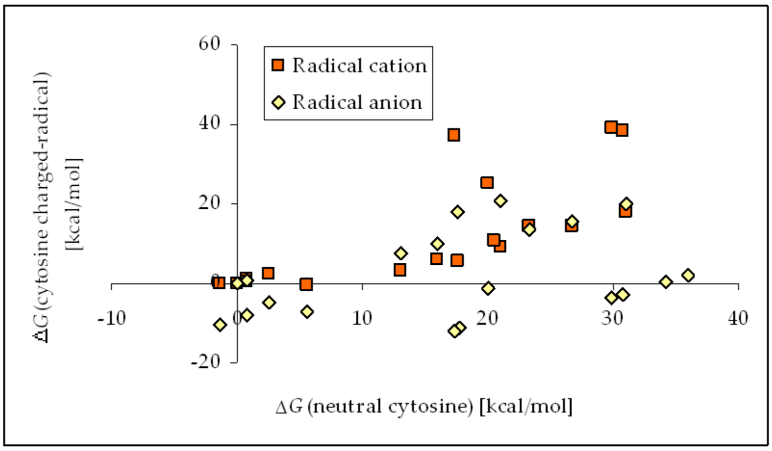

(c) Isocytosine

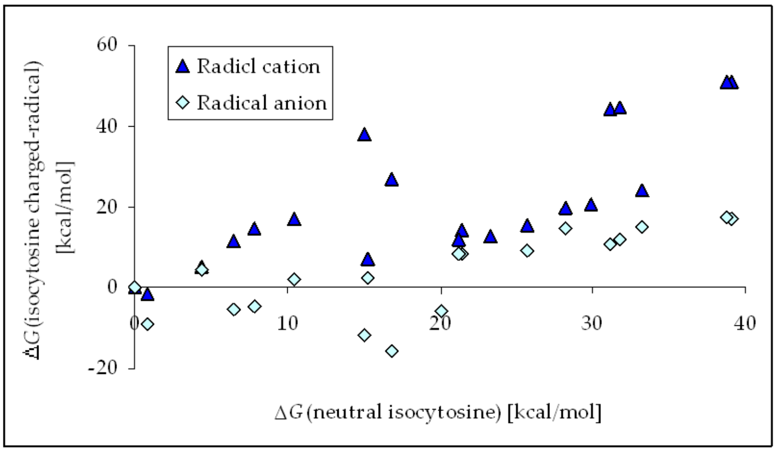

Figure S7. Plots between HOMED8s of neutral and ionized tautomers-rotamers of uracil (a), cytosine (b), and isocytosine (c). DFT data taken from refs [28,57,62,63,64,65].

(a) Uracil

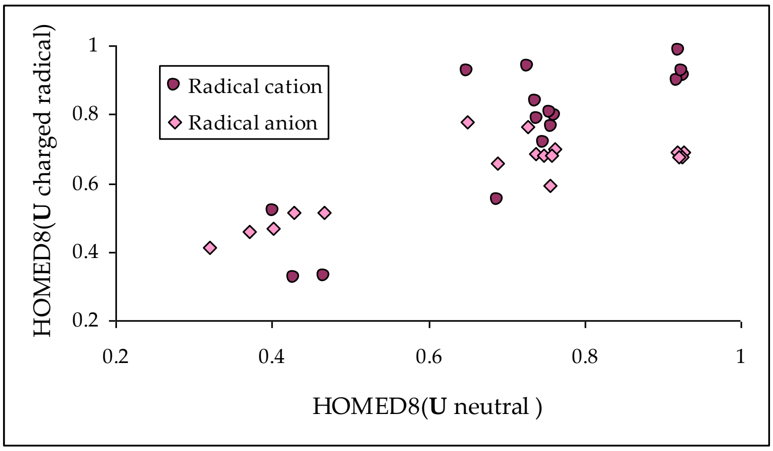

(b) Cytosine

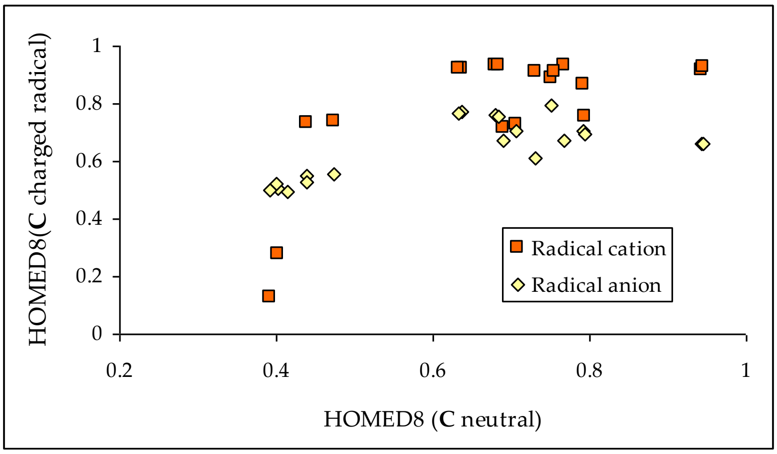

(c) Isocytosine

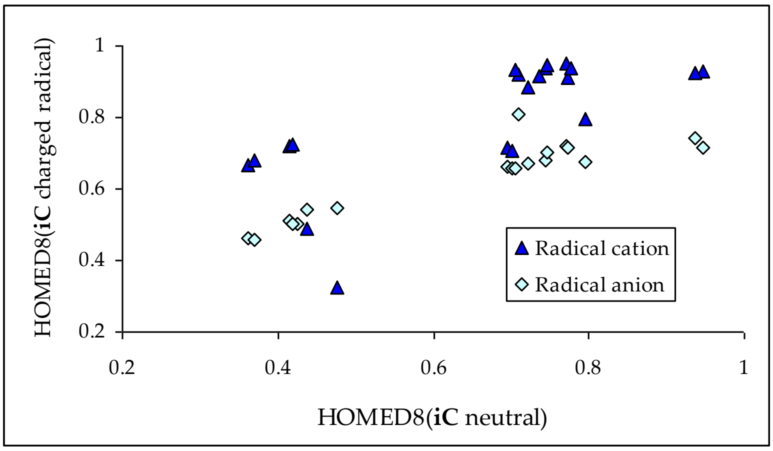

List of abbreviations applied in the main text

**2APM**	2-aminopyrimidine
**4APM**	4-aminopyrimidine
**5APM**	5-aminopyrimidine
**2APY**	2-aminopyridine
**3APY**	3-aminopyridine
**4APY**	4-aminopyridine
**2HOPM**	2-hydroxypyrimidine
**4HOPM**	4-hydroxypyrimidine
**5HOPM**	5-hydroxypyrimidine
**2HOPY**	2-hydroxypyridine
**3HOPY**	3-hydroxypyridine
**4HOPY**	4-hydroxypyridine
**A**	adenine
α	normalization constant
AM1	Austin Model 1
B3LYP	Becke-style three-parameter hybrid exchange functional with non-local correlation functional of Lee, Yang, and Parr
**C**	cytosine
CASSCF	complete active space self-consistent field
CCSD(T)	coupled cluster method including single-, double- and triple-excitation
CID	collision-induced dissociation
DFT	density functional theory
E	energy
EA	electron affinity
ESR	electron spin resonance
FT-MW	Fourier transform microwave
**G**	guanine
G	Gibbs energy
G2	Gaussian-2 theory
G2(MP2)	MP2 variant of Gaussian-2 theory
G4	Gaussian-4 theory
G*n*	Gaussian-*n* theory
**HX**	hypoxanthine
H	enthalpy
HF	Hartree-Fock self-consistent field
HOMA	harmonic oscillator model of aromaticity
HOMED	harmonic oscillator model of electron delocalization
**HX**	hypoxanthine
**iC**	isocytosine
ICR	ion cyclotron resonance
IE	ionization energy
IR	infrared
IRMPD	infrared multiple-photon dissociation
IUPAC	International Union of Pure and Applied Chemistry

K	equilibrium constant
LA-MB-FT-MW	laser-ablation molecular-beam Fourier-transform microwave
LFER	linear free-energy relationship
**M**	molecule
MNDO	modified neglect of diatomic overlap
MP2	second-order Møller-Plesset (perturbation theory)
MP*n*	*n*-order Møller-Plesset (perturbation theory)
MS	mass spectrometry
MW	microwave
n	number of bonds
NMR	nuclear magnetic resonance
PM3	parametric method 3
PES	photoelectron spectroscopy
QCISD(T)	quadratic configuration interaction method including single-, double- and triple-excitation
R	molar gas constant
REMPI	resonantly enhanced multiphoton ionization
R_d	double bond-length
R_i	bond-length in investigated compound
R_o	optimum bond-length
R_s	single bond-length
RNA	ribonucleic acid
S	entropy
**T**	thymine
T	temperature
TD-DFT	time-dependent density functional theory
**U**	uracil
**UA**	uric acid
UV	ultraviolet
UVPD	UV-vis photodissociation
**X**	xanthine
ZEKE	zero kinetic energy
